# Late-Stage Serine Modification Enables Noncanonical Peptide Synthesis

**DOI:** 10.1021/jacs.5c11065

**Published:** 2025-08-25

**Authors:** Zhenyan Guo, Tianning Diao

**Affiliations:** Department of Chemistry, 5894 New York University, 100 Washington Square East, New York, New York 10003, United States

## Abstract

Noncanonical amino acids play a critical role in enhancing drug efficacy, specificity, pharmacokinetics, and other key therapeutic properties. However, their incorporation into peptides or small molecules often presents significant synthetic challenges. Late-stage modification of natural residues, after the primary structural framework of a molecule is established, offers an efficient strategy for generating analogue libraries. Serine, one of the most abundant natural amino acids, remains underutilized due to the incompatibility of existing deoxygenative methods with complex peptides. Currently, no method is available for the late-stage modification of serine residues in peptides through carbon–carbon bond formation. Here, we address this gap by developing a site-selective, late-stage deoxygenative functionalization of serine residues. Inspired by automated DNA synthesis, we employ a phosphoramidite reagent in combination with a photocatalytic system to achieve efficient serine deoxygenative activation and radical addition to diverse acceptors, enabling the transformation of serine into various noncanonical residues such as homoglutamine, homoglutamic acid, 5-hydroxynorvaline, phosphonates, and alanine-3-*d*_1_. The method proved compatible with complex peptides such as enkephalin, bradykinin, and α-MSH both on solid support and in solution. The broad substrate scope, operational robustness, and high chemoselectivity of this approach position it as a versatile platform for peptide diversification and the advancement of medicinal chemistry.

## Graphical abstract

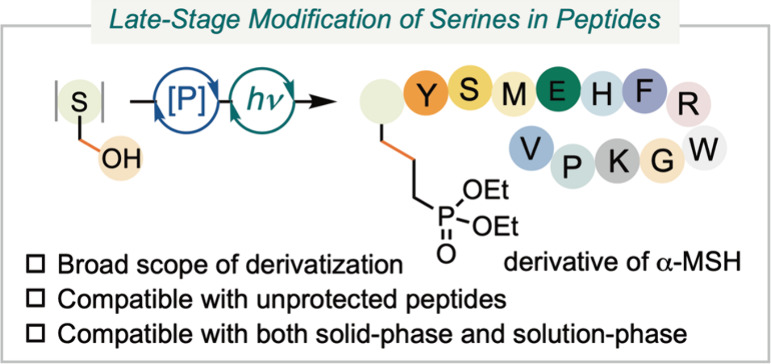

## Introduction

Noncanonical amino acids have been widely incorporated into peptides and small-molecule drugs to enhance target binding affinity and specificity, as well as to optimize key properties such as solubility, physiological stability, and cellular permeability.^1,2^ For example, varying the side chains at a specific amino acid position in a P2Y_12_ antagonist generated analogues with a wide range of efficacy; notably, the phosphate-containing analogue exhibited high binding affinity and potent inhibition of ADP-induced platelet aggregation in human platelet-rich plasma (PRP) (Scheme 1A).^3^ While numerous emerging methods aim to synthesize noncanonical amino acids,^4,5^ modification of peptides or small molecules, after their core structural framework is established, offers an efficient strategy for generating analogue libraries. This expanded access to chemical diversity facilitates drug discovery, enables structure–activity relationship (SAR) studies, and advances chemical biology.^6,7^

**Scheme 1:**
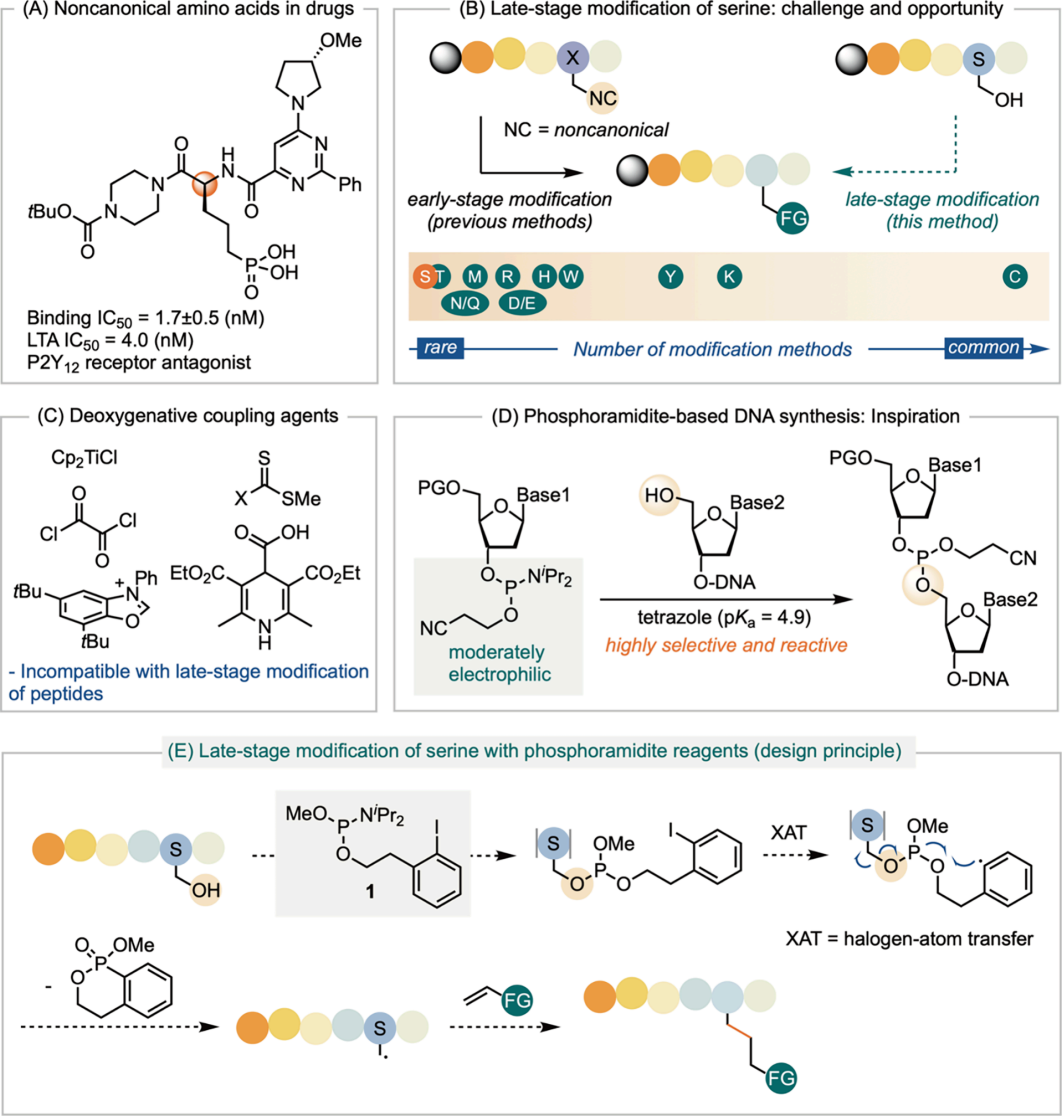
Challenge and Opportunities in Late-Stage Peptide Modification via Selective Conversion of Serine (S), and the Design of Phosphoramidite Reagents for Deoxygenative Coupling of Serine

A primary challenge in peptide diversification is achieving chemoselectivity and site-specificity amid various functional groups present in peptides. A common strategy involves early stage modification by incorporating noncanonical amino acids with functional handles during solid-phase peptide synthesis (SPPS), followed by downstream derivatization such as cross-coupling (Scheme 1B).^8–13^ However, the need for exogenous functionalities prevents the applicability of this approach to native biomolecules. In contrast, late-stage modification of native amino acid residues–particularly through carbon–carbon bond formation–offers greater flexibility for installing functional groups.^14–21^ Despite its potential, late-stage modification of native peptides remains a significant challenge.

Serine, one of the most abundant natural amino acids, represents a compelling yet underutilized site for bioconjugation and derivatization. Serine is frequently found in solvent-exposed, flexible loop or linker regions of peptides and proteins.^22^ Thus, serine modification can preserve peptide conformation while enabling functional modulation. Existing methods for selective serine modification are exceedingly rare.^23^ Recent efforts have primarily focused on phosphorylation, which, while biologically relevant, offer limited utility for the synthesis of noncanonical residues in drug discovery applications.^24^ Currently, no method is available for the late-stage modification of serine residues in peptides through carbon–carbon bond formation. This challenge likely stems from the lack of chemoselectivity in previously developed deoxygenative coupling reagents targeting alcohols, which are often incompatible with the structural complexity and functional group diversity of peptides (Scheme 1C*).* ^25–34^

Phosphoramidites are mildly electrophilic reagents that enable alcohol protection with high selectivity, efficiency, and broad functional group compatibility (Scheme 1D). This reactivity has been instrumental in the development of automated DNA synthesis, where phosphoramidite reagents selectively phosphorylate the C-3 hydroxyl group of oligonucleotides under mildly acidic conditions.^35^ A phosphite bearing a pendant iodoarene has been reported to undergo radical cyclization, resulting in deoxygenative radical generation; however, the reliance on PCl_3_ restricts the general applicability of this approach.^36^ A related phosphoramidite reagent has demonstrated site-selective alcohol reduction when used in conjugation with molecular recognition catalysts.^37^ Nevertheless, both classes of reagents have been applied exclusively to alcohol reduction, and their potential for broader functionalization or cross-coupling and late-stage functionalization of biomolecules remains largely unexplored.

Herein, we draw inspiration from phosphoramidite-enabled DNA synthesis, leveraging the high selectivity of these reagents toward alcohol activation to develop a general strategy for the late-stage deoxygenative functionalization of serine in peptides, achieving chemoselectivity amid diverse functional groups (Scheme 1E). We hypothesize that phosphoramidite **1**, bearing a tethered aryl iodide, can readily activate to generate a radical intermediate upon halogen-atom abstraction. Cyclization of the resulting aryl radical onto the phosphite promotes β-scission of the serine C–O bond, forming an alanine carbon radical. This radical then undergoes Giese addition to a variety of acceptors, transforming serine into noncanonical amino acids. This approach provides a general and broadly compatible platform for late-stage serine modification in peptides.

## Results and Discussion

We tested our hypothesis by preparing phosphoramidite **1**,^37^ which phosphitylated serine **2** in the presence of 5-methyl-tetrazole to afford phosphite **3** in 92% isolated yield (Scheme 2A).^38^ A brief round of catalyst development led to the identification of photoredox conditions employing photocatalyst **6**,^39^ IDM-BH_3_ 7 (IDM = dimethyl imidazolylidene),^40^ and HCO_2_K. Under these conditions, Giese addition of **3** to vinyl nitrile afforded product **4** in 87% yield, along with 12% of dehydroalanine (Dha) **5** as a byproduct (Scheme 2A, entry 1).^40^ Given the low UV absorption of **4**, we isolated product **18** using benzyl acrylate as the acceptor to assess potential epimerization under photoredox conditions. Comparison of isolated product **18** with a racemic sample, obtained from radical addition to Dha **5,** ^41^ by chiral HPLC confirmed that no epimerization occurred at the α-carbon. When the modification of **2** with **1** was followed by a one-pot Giese addition to vinyl nitrile, without isolating **3**, product **4** was obtained in 70% overall yield.

**Scheme 2:**
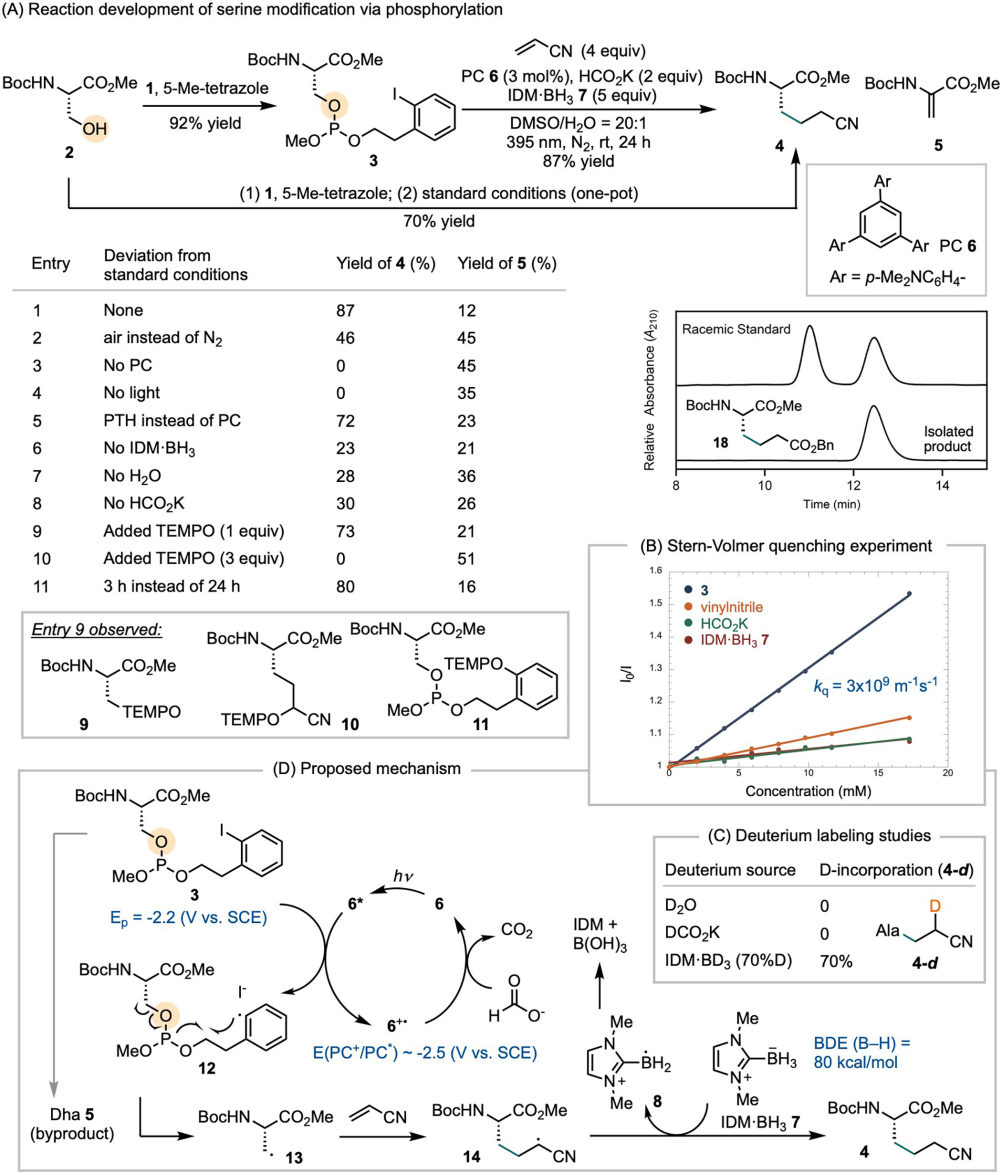
Reaction Development and the Proposed Mechanism

Control experiments involving the omission or variation of individual components in the catalytic system helped elucidate the role of each variable (Scheme 2A). Exposure of the reaction to air resulted in a significant decrease in the yield of **4**, suggesting that O_2_ is detrimental, likely due to quenching of radical intermediates (entry 2). Omitting either the photocatalyst or light completely inhibited product formation (entries 3–4). Photocatalyst **6** could be replaced by 10-phenylphenothiazine (PTH),^42^ albeit with a slightly lower yield (entry 5). Excluding other components, including IDM-BH_3_ 7, H_2_O, or HCO_2_K, also led to decreased yields (entries 6–8). Water is essential for dissolving HCO_2_K. Addition of one equivalent of TEMPO resulted in a 73% yield of **4**, along with a mixture of **9**, **10**, and **11** observed in HRMS, which likely arose from trapping of radical intermediates by TEMPO (entry 9). The product distribution varied with the amount of TEMPO: increasing the TEMPO loading to three equivalents led to an increased formation of **11** and complete inhibition of **4**, reflecting competition between radical trapping by TEMPO and the desired reaction pathway (entry 10). Finally, shortening the reaction time to 3 h maintained a comparable yield of **4** (entry 11).

We then conducted Stern–Volmer quenching experiments by measuring the decrease in photon emission intensity of photocatalyst **6** as a function of the concentrations of possible quenchers: phosphite **3**, IDM-BH_3_ 7, HCO_2_K, and vinyl nitrile (Scheme 2B). While all components exhibited some degree of quenching, phosphite **3** was the most efficient quencher of the photoexcited state of **6**, with a quenching constant (*k*_q_) of 3 × 10^9^ M^–1^s^–1^. Intermittent light-irradiation experiments revealed that the reaction rate significantly decreased in the absence of light and resumed upon reillumination (Figure S21), supporting a photocatalytic mechanism. A slight conversion was observed during dark periods, which may be attributed to a minor chain side pathway, wherein IDM-BH_2_^•+^ 8 undergoes halogen-atom abstraction with **3**, propagating to form product.

Deuterium labeling studies further revealed that the use of D_2_O or DCO_2_K did not result in deuterium incorporation into product **4** (Scheme 2C). In contrast, using IDM-BD_3_ led to stoichiometric deuterium incorporation to the α-position of product **4**. These results suggest that IDM-BH_3_ 7 serves as the primary hydrogen atom donor. Additionally, no H/D scrambling occurs between IDM-BD_3_ and other potential H-atom donors.

The optimization and mechanistic data are consistent with the mechanism proposed in Scheme 2D. Photoexcitation of photocatalyst (PC) **6** generates a strongly reducing excited state 6* (E_1/2_ ∼ −2.5 V vs SCE),^39^ which undergoes single-electron transfer to **3**, producing the oxidized PC **6** ^**·+**^ and intermediate **12**. Radical cyclization of **12**, followed by β-scission, affords the alanine radical **13**, which adds to vinyl nitrile to form intermediate **14**. This radical intermediate is then reduced by IDM-BH_3_ 7, consistent with the deuterium labeling studies that identifies IDM-BH_3_ 7 as the primary hydrogen-atom donor. The oxidized PC **6** ^**·+**^ is subsequently reduced by formate, regenerating the ground state PC **6**.^43^ In the presence of TEMPO, the formation of trapping products **9**, **10**, and **11** suggests that the transformations from **12** to **4** proceed at comparable rates, and that the product distribution is sensitive to the concentration of radical trapping agents in solution.

Upon optimizing the conditions for serine modification, we explored the method’s compatibility with a broad range of radical acceptors (Scheme 3). In addition to vinyl nitrile, the radical generated from serine **3** successfully added to vinyl phosphate to generate **15**, acrylamides to form **16** and **17**, and acryl esters to generate **18**-**20**. Notably, phosphate ester **15** was previously synthesized in six steps from methionine^44^ and homoglutamine **17** is of significant interest in chemical biology studies.^45^ Ester **20** can be readily derivatized to introduce functional handles, enabling the synthesis of fluorescent probe **21** via “Click” chemistry. Alternatively, deoxygenative coupling allows for direct derivatization of serine using an acrylate ester prefunctionalized with D-biotin, affording **22**. The reaction conditions also tolerate unprotected acrylic acid, yielding homoglutamic acid **23**. Furthermore, the radical generated through deoxygenative derivatization of serine engaged a wider array of acceptors, including vinyl pyridine (**24**), dehydroalanine (**25**), α,α-disubstituted alkenes (**26** and **27**), and electron-rich acceptors such as vinyl silanes (**28**) and vinyl boranes (**29**). Oxidation of **29** afforded 5-hydroxynorvaline **30**, a rare amino acid. Additional acceptors include [1.1.1]­propellane, yielding **31**; *N,N*-diphenyl methacrylamide, which underwent addition and arylation to give **32**; and phenylacetylene, affording **33**. Moreover, the methodology extends beyond serine. Galactose also underwent successful functionalization under these conditions, providing **34**.

**Scheme 3:**
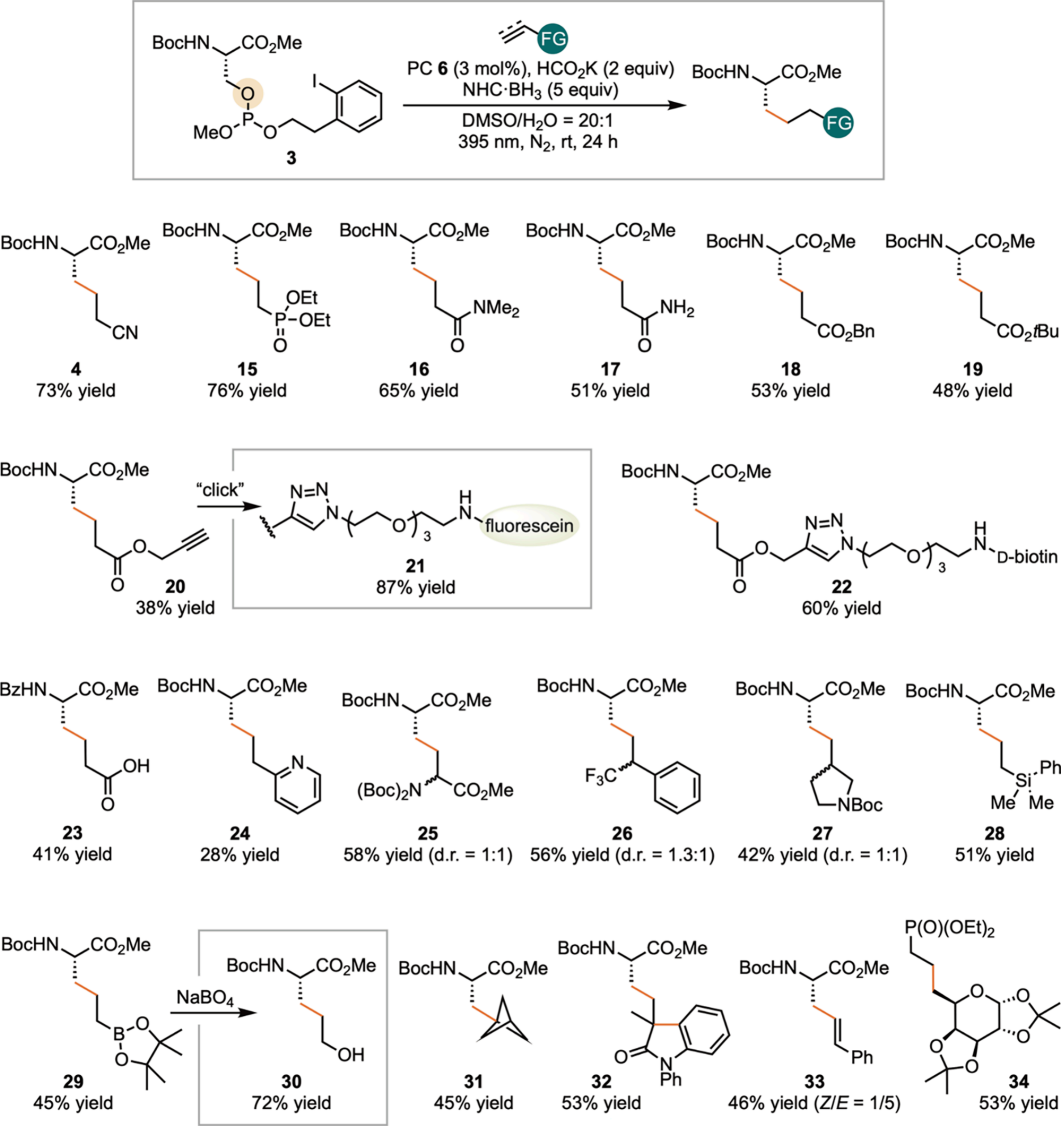
Scope of Coupling Partners for the Conversion of Serine into Noncanonical Amino Acids^a^

a Isolated yields, Reaction conditions: **3** (0.19 mmol), radical acceptor (4.0 equiv), PC (3 mol %), HCO_2_K (2.0 equiv), NHC·BH_3_ (5.0 equiv) in DMSO/H_2_O = 20:1 (0.025 M), at room temperature for 24 h.

To evaluate the selectivity of phosphoramidite reagent **1** for serine over other nucleophilic residues, we conducted competition experiments with threonine (Thr), tyrosine (Tyr), lysine (Lys), and cysteine (Cys) (Scheme 4). Ser was phosphorylated four times more readily than Thr, leading to selective modification in the subsequent Giese addition step. Although Tyr and Lys were phosphorylated with comparable efficiency to Ser, their phosphorylated intermediates did not undergo Giese addition and were instead hydrolyzed, allowing recovery of the unmodified Tyr and Lys residues. Cys did not undergo phosphorylation, and its presence did not interfere with Ser modification in the second step. These results highlight the overall selectivity of the phosphoramidite **1**-based modification strategy. The yields of Ser modification in the second step were not optimized in these competition experiments, as excess reagent **1** from the phosphorylation step was carried over and consumed a portion of the reductant during the second step.

**Scheme 4:**
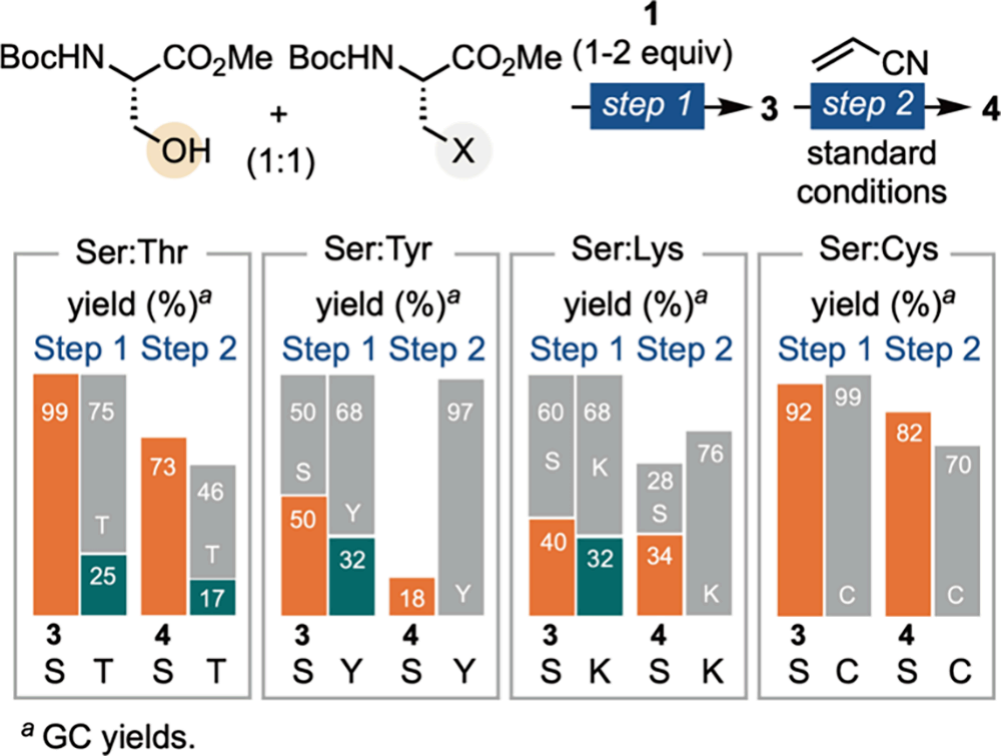
Selectivity for Serine over Other Nucleophilic Residues

We next applied phosphoramidite reagent **1** and the deoxygenative coupling protocol to the late-stage modification of peptides. We first synthesized solid-supported peptide **35** using standard solid-phase peptide synthesis (SPPS) protocols (Scheme 5). Instead of the conventional *tert*-butyl protected serine, we incorporated TBS-protected serine residue (TBS = *tert*-butyldimethylsilyl). Following peptide elongation, we selectively removed the TBS protecting group using TBAF (TBAF = tetrabutylammonium fluoride). Treating peptide **35** with reagent **1** was followed by photoredox deoxygenative coupling with vinyl phosphite **36** under standard conditions.

**Scheme 5:**
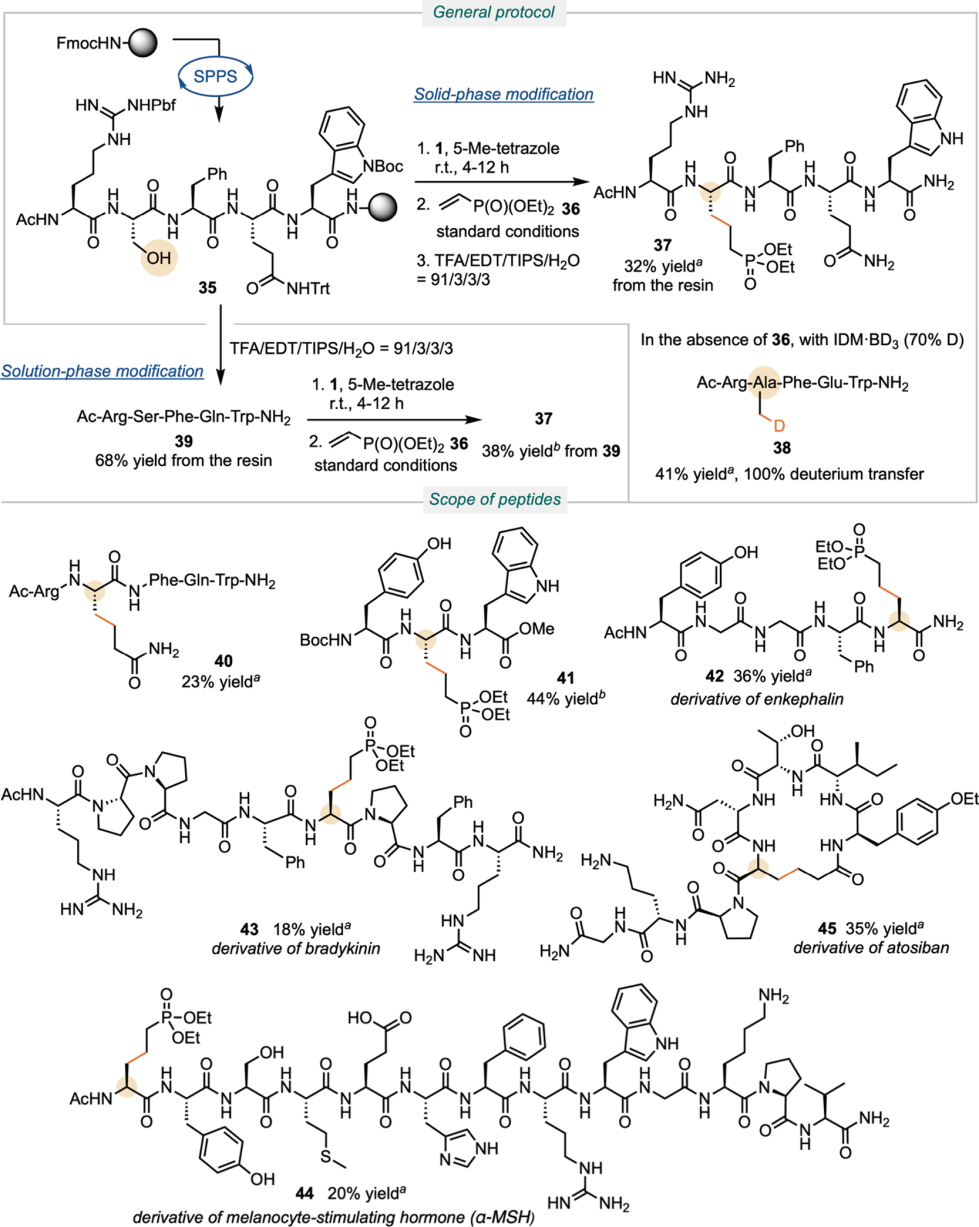
Late-Stage Serine Modification in Peptides on Solid Support and in Solution

a Isolated yields after prep-HPLC purification, calculated based on the initial loading of Rink amide resin. Modification performed on solid support.
b Modification performed in solution.

Subsequent cleavage from the solid support afforded peptide **37**, in which the serine residue was converted into a diethyl phosphate. The overall isolated yield was 32% over 16 steps, starting from Rink amide resin. The major byproducts were the reduction of serine to alanine (Ala) and the formation of serine phosphate, derived from ejection of a methyl radical instead of an Ala radical from intermediate **12**. When the radical acceptor **36** was omitted and IDM-BD_3_ was applied, we observed quantitative deuterium incorporation, yielding peptide **38**, in which the serine residue was converted to Ala-3-*d*_1_. This strategy offers a useful approach for generating deuterated peptides, which may offer improved pharmacokinetic properties and/or toxicity profiles due to isotope effects.^46^ Additionally, this method could enable radiolabeling applications for drug metabolism and pharmacokinetic studies.

Alternatively, we cleaved and purified peptide **35** to afford peptide **39** with all residues unprotected. Applying the same deoxygenative coupling protocol in solution yielded peptide **37** in 38% isolated yield, starting from **39**. The phosphorylation step exhibited approximately 50% conversion, potentially due to the influence of the peptide’s hydrophilic side chains on the local pH. The photoredox Giese addition proceeded efficiently, achieving full conversion. Although this comparison indicates that solid-phase synthesis provides a higher overall yield than the solution-phase modification, the successful functionalization of unprotected peptide **39** demonstrates that late-stage deoxygenative modification of serine is feasible in the presence of other unprotected polar residues, including Arg, Gln, and Trp. This opens opportunities for applying the method to existing peptides and proteins.

To further demonstrate the compatibility of the method, we derivatized peptide **35** using acrylamide as the radical acceptor, converting the serine residue into homoglutamine to afford peptide **40** (Scheme 5). Among nucleophilic residues, Tyr can undergo phosphorylation as a competing pathway. Treatment of unprotected Boc-Tyr-Ser-Trp-OMe with phosphoramidite **1** in solution led to phosphorylation at both Tyr and Ser during the first step, as observed by LC-MS. However, upon proceeding to the second step, the desired Ser-modified product **41** was cleanly obtained. Because deoxygenative coupling does not occur on Tyr, the Tyr-phosphite intermediate hydrolyzed , restoring the native Tyr residue, consistent with the competition experiments (Scheme 4).

The exquisite selectivity provided the foundation for extending the approach to a series of biologically relevant peptides, converting serine residues into diethyl phosphates to afford peptides **42**-**44**. Among these examples, enkephalin is an endogenous opioid produced in the brain.^47^ Bradykinin plays a key role in inflammation and is a known drug target for the treatment of hereditary angioedema.^48^ α-Melanocyte-stimulating hormone (α-MSH) is an endogenous agonist of the melanocortin receptor 4 (MC4R) with anti-inflammatory and antipyretic activities.^49^ Moreover, we synthesized an atosiban analogue **45** by introducing an acrylate at the *N-*terminus during SPPS and applying the serine modification protocol to induce intramolecular cyclization. Atosiban is a medication used to delay imminent premature labor in selected pregnant women.^50^ These examples cover a wide range of amino acid side chains, demonstrating the compatibility of our protocol with both hydrophilic (Arg, His, Lys, Glu, Thr, Asn) and hydrophobic (Gly, Val, Ile, Met, Phe, Pro, Tyr, Trp, Cys) residues.

## Conclusions

In summary, we have developed a phosphorylation-enabled, deoxygenative Giese addition protocol that allows for late-stage modification of serine residues in peptides. The reaction proceeds via cyclization of an aryl radical onto a phosphite group, followed by β-scission to deoxygenate serine and generate an alanine radical. This radical intermediate can be efficiently trapped by a variety of acceptors, including vinyl nitrile, vinyl phosphates, and acrylates, enabling the transformation of serine into various noncanonical residues such as homoglutamine, homoglutamic acid, 5-hydroxynorvaline, phosphonates, and Ala-3-*d*_1_. Notably, this strategy proved compatible with complex peptides bearing a wide variety of hydrophilic and hydrophobic residues, including enkephalin, bradykinin, and α-MSH. The broad scope establishes this approach as a robust and versatile tool for generating peptide libraries with single-residue variants, thereby facilitating SAR studies and advancing medicinal chemistry. Moreover, the orthogonal reactivity of serine relative to other amino acid side chains opens new avenues for peptide multifunctionalization, with potential applications in biochemistry and cell biology.

## Supplementary Material



## Notes

Full experimental procedures, characterization data, and NMR spectra (PDF)
