# Supplementary material for: Late-Stage Serine Modification Enables Noncanonical Peptide Synthesis
Source: J Am Chem Soc. 2025 Aug 25;147(36):33127–35. doi: 10.1021/jacs.5c11065 (PMC12426918; doi:10.1021/jacs.5c11065)
Supplement: Supplementary file 1 [file ja5c11065_si_001.pdf]

Supporting Information

Late-Stage Serine Modification Enables Noncanonical Peptide Synthesis

Zhenyan Guo[1] and Tianning Diao[1]*

[1]*Department of Chemistry, New York University, 100 Washington Square East, New York, NY 10003, United States*

*Email: diao@nyu.edu

Table of Contents

1. General
2. Synthesis of Catalysts and Substrates
3. Experimental Procedures
4. Reaction Optimization and Control Experiments
5. Experimental Data for Deoxygenative Giese Reaction of Serine
6. Stern-Volmer Quenching Studies
7. Cyclic Voltammetry Measurement
8. Radical Trapping Experiments with TEMPO
9. Deuterium Labeling Experiments
10. Light on and off experiments
11. Confirmation of the Retention Stereochemistry of **18**
12. NMR Spectra for New Compounds
13. References

1. General

All air- and moisture-sensitive reactions were conducted under a nitrogen atmosphere using either a glovebox or standard Schlenk technique. Solvents were dried and deoxygenated by a Grubbs' type solvent purification system or by performing three freeze-pump-thaw cycles. Deuterated solvents were purchased from commercial suppliers. All other reagents were obtained from commercial sources and used without further purification unless otherwise specified.

All NMR spectra (^{1}H, ^{13}C, ^{31}P, ^{19}F, ^{11}B) were recorded on Bruker Avance 400 MHz, 500 MHz, 600 MHz or 800 MHz spectrometers. Chemical shifts of the ^{1}H resonances are reported in ppm relative to tetramethylsilane (TMS), with the residual solvent resonance ($CDCl_3$, δ = 7.26 ppm, MeOD, δ = 3.31 ppm) as the internal reference. Spectra are reported as the following: chemical shift (δ ppm), multiplicity (s = singlet, bs = broad singlet, d = doublet, t = triplet, q = quartet, m = multiplet), coupling constant (Hz), and integration. Chemical shifts of the ^{13}C were reported in ppm relative to TMS with the solvent resonance used as the internal reference ($CDCl_3$, δ = 77.2 ppm, MeOD, δ = 49.00 ppm).

GC data was obtained using a Shimadzu GC-2010 Plus with a Restek Rxi-5ms column (L 15 m, ID 0.25 mm, DF 0.25 µm). High resolution mass spectra (HRMS) were collected on Agilent 6224 TOF LC/MS. Diastereomers were separated and analyzed by Agilent 1260 Infinity HPLC equipped with CHIRALPAK ODH columns. LC-MS was performed on a Thermo Scientific TSQ Fortis Plus Triple Quadrupole mass spectrometer coupled to a Zorbax RRHD Eclipse Plus C18 column (95 Å, 1.8 µm, 3.0 x 100 mm). Reactions were monitored by thin-layer chromatography (TLC) on Merck silica gel 60 F254 plates and compounds were visualized by UV light (254 nm) or by staining with Permanganate or Ninhydrin. Flash column chromatography was performed using SiliaFlash® Irregular Silica Gel, F60 40 - 63 µm, 60 Å, purchased from Silicycle®.

2. Synthesis of Catalysts and Substrates

1,3-dimethylimidazol-2-ylidene borane (8)[1]

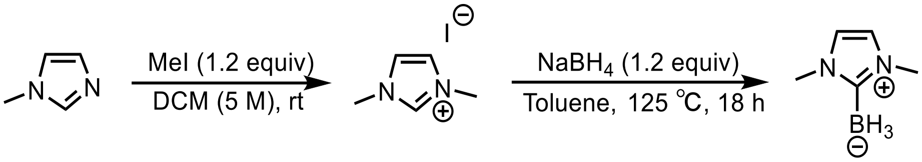

Iodomethane (8.5 g, 60.0 mmol) was added dropwise over 10 min to a solution of the 1-methylimidazole (4.1 g, 50.0 mmol, 5 M) in dichloromethane (DCM). The reaction mixture was stirred at room temperature for 1 h, then concentrated under vacuum to give the product (11.2 g, 100% yield). The imidazolium salt was confirmed by a ^{1}H NMR spectrum, and then the sample was used directly in the next step without further purification.

Sodium borohydride (2.3 g, 60.0 mmol) was added to a suspension of crude imidazolium salt (50.0 mmol) in toluene (50 mL). The reaction was allowed to stir at 125 °C for 18 h. The hot reaction solvent was carefully decanted from the insoluble residue, which was then washed with hot toluene (2 × 50 mL). The combined organic solvents were concentrated and purified by flash chromatography (2: 1, hexanes to ethyl acetate) to give the product as fine white crystals (3.2 g, 57% yield).

^{1}H NMR (400 MHz, $CDCl_3$) δ 6.79 (s, 2H), 3.74 (s, 6H), 1.01 (dd, J = 172.8, 86.3 Hz, 3H).

^{11}B NMR (128 MHz, $CDCl_3$) δ -37.51 (q, J = 86.4 Hz).

[(*N,N*)Me2]3TAPB (6)[2]

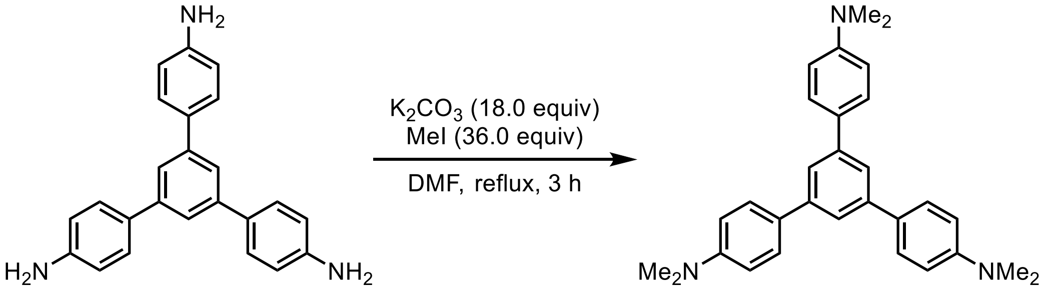

TAPB (351.0 mg, 1.0 mmol) was dissolved in anhydrous DMF (10 mL) and added to a suspension of K_2CO_3 (2.5 g, 18.0 mmol) in anhydrous DMF (10 mL). Methyl iodide (2.4 mL, 36.0 mmol) was added dropwise, and the reaction mixture was heated to reflux for 3 h. After cooling to room temperature, the reaction mixture was extracted with diethyl ether. The combined organic layer was dried with Na_2SO_4 and concentrated under vacuum. The crude product was purified by flash column chromatography (1: 1, hexanes to ethyl acetate) to give the product (4.5 g, 72% yield).

^{1}H NMR (400 MHz, $CDCl_3$) δ 7.77 – 7.49 (m, 9H), 6.84 (d, J = 8.3 Hz, 6H), 3.01 (s, 18H).

2-(2-Iodophenyl)ethanol (S1)[3]

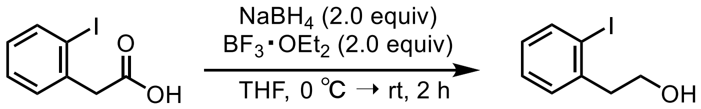

To a stirred solution of 2-iodophenylacetic acid (5.2 g, 20.0 mmol) in THF (40 mL) was added $NaBH_4$ (1.5 g, 40.0 mmol) in portions at 0 °C, followed by slow addition of $BF_3 \cdot Et_2O$ (5.0 mL, 40.0 mmol). The reaction mixture was stirred from 0 °C to room temperature over 2 h. Methanol (15 mL) was then added dropwise, followed by 1M HCl (15 mL). The reaction mixture was diluted with ethyl acetate (30 mL), and the organic phase was separated and dried over Na_2SO_4. The solvent was evaporated under vacuum, and the crude product was purified by flash column chromatography (2: 1, hexanes to ethyl acetate) to give the product as a colorless oil (4.5 g, 90% yield).

^{1}H NMR (400 MHz, $CDCl_3$) δ 7.86 (dd, J = 7.9, 1.2 Hz, 1H), 7.40 – 7.21 (m, 2H), 6.95 (ddd, J = 8.0, 6.8, 2.3 Hz, 1H), 3.89 (t, J = 6.7 Hz, 2H), 3.05 (t, J = 6.8 Hz, 2H), 1.44 (s, 1H).

Di-isopropyl ammonium tetrazolide (S2)

To a stirred solution of tetrazole (0.3 g, 4 mmol) in anhydrous acetonitrile (5 mL) was added diisopropylamine (0.8 g, 8.0 mmol). A white precipitate was formed immediately and was collected by filtration. The solid was washed with acetonitrile and dried under vacuum to yield the product as a white crystalline solid.

2-iodophenethyl methyl diisopropylphosphoramidite (1)[4-6]

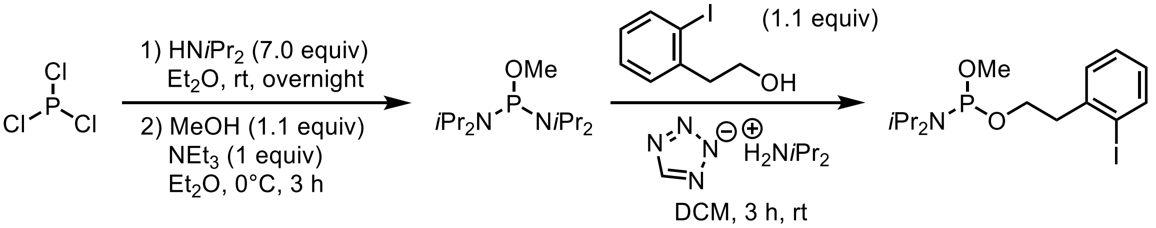

Diisopropylamine (22 mL, 157.0 mmol) was added to a flame-dried 500 mL round-bottom flask and diluted with anhydrous Et_2O (20 mL). The solution was degassed under a nitrogen atmosphere in an ice bath for 10 mins. A solution of phosphorus trichloride (3.1 g, 23.0 mmol) in anhydrous Et_2O (10 mL) was added to the reaction dropwise. After complete addition, the ice bath was removed, and the reaction was allowed to warm to room temperature. Complete conversion of the phosphorus trichloride (δ 201 ppm in $CDCl_3$) to product (δ 141 ppm in $CDCl_3$) was monitored by ^{31}P NMR. The reaction mixture was filtered on celite and Na_2SO_4 to a round-bottom flask to remove diisopropylamine hydrochloride, and the precipitate was washed with anhydrous Et_2O. The combined filtrates were directly used in the next step without further purification.

To the crude filtrate was added dropwise a mixture of triethylamine (3.2 mL, 23.0 mmol) and anhydrous MeOH (1.1 mL, 25.0 mmol) in anhydrous Et_2O (10 mL) at 0 °C under nitrogen. The mixture was stirred at 0 °C for 3 h. Complete conversion of both starting materials was monitored by ^{1}H NMR and ^{31}P NMR (δ 131 ppm in $CDCl_3$). The mixture was filtered through Celite and Na_2SO_4, the solvent was removed under vacuum to afford the desired product as a colorless oil (5.1 g, 85% yield), which was used without further purification.

To a flame-dried 100 mL round bottom flask equipped with a stir bar were added 20 mL anhydrous DCM, methyl tetraisopropyl phosphorodiamidite (2.4 g, 8.9 mmol), and diisopropyl ammonium tetrazolide (690 mg, 4.0 mmol). This was followed by the addition of 2-(2-iodophenyl) ethanol (2.0 g, 8.1 mmol). The reaction mixture was stirred at room temperature under nitrogen atmosphere. After 3 h, the reaction was monitored by ^{31}P NMR (δ 147 ppm in $CDCl_3$) to confirm complete consumption of the starting material, filtered with the cotton, and concentrated under vacuum. The resulting residue was purified by flash column chromatography with 90: 5: 5, hexanes to ethyl acetate to triethylamine to give the product as a viscous oil (3.1 g, 94% yield).

^{1}H NMR (400 MHz, $CDCl_3$) δ 7.80 (dd, J = 7.9, 1.2 Hz, 1H), 7.31 – 7.20 (m, 2H), 6.89 (ddd, J = 7.9, 6.7, 2.3 Hz, 1H), 3.90 – 3.72 (m, 2H), 3.57 (dp, J = 9.9, 6.8 Hz, 2H), 3.40 (d, J = 13.1 Hz, 3H), 3.07 (t, J = 7.2 Hz, 2H), 1.16 (dd, J = 9.8, 6.8 Hz, 12H).

^{13}C NMR (101 MHz, $CDCl_3$) δ 141.37, 139.43, 130.53, 128.16 (d, J = 5.3 Hz), 100.72, 62.66 (d, J = 18.4 Hz), 50.58 (d, J = 17.7 Hz), 42.76 (d, J = 12.3 Hz), 42.52 (d, J = 7.2 Hz), 24.78, 24.71, 24.64.

^{31}P NMR (162 MHz, $CDCl_3$) δ 147.61.

HRMS (ESI-TOF) m/z: $[M+H]^+$ calcd. for $C_{15}H_{26}INO_3P$, 410.0740; found, 410.0689.

Methyl *N*-(tert-butoxycarbonyl)-O-((2-iodophenethoxy)(methoxy)phosphaneyl)-L-serinate (3)

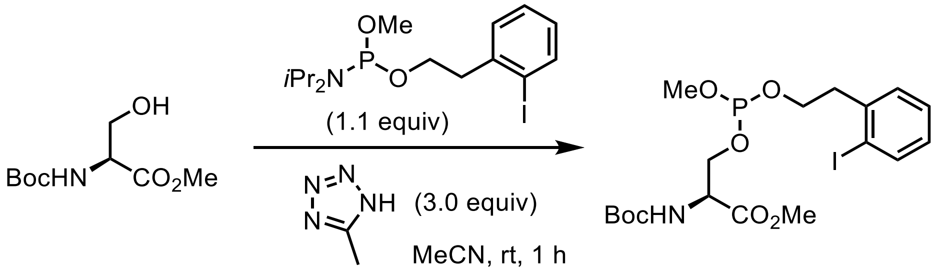

To a solution of the Boc-Ser-OMe (2.2 g, 10.0 mmol) in anhydrous acetonitrile at room temperature was added 5-methyltetrazole (2.5 g, 3.0 equiv), and the mixture was stirred for 30 min under nitrogen atmosphere. 2-iodophenethyl methyl diisopropylphosphoramidite (4.3 g, 1.1 equiv) was added and stirred until the reaction was complete in 1 h. The reactions were monitored by TLC using hexanes/ ethyl acetate and concentrated under vacuum. The residue was purified by

flash column chromatography using with 90: 10: 5, hexanes to ethyl acetate to triethylamine to give the product as a viscous oil (5.0 g, 92% yield).

^{1}H NMR (400 MHz, $CDCl_3$) δ 7.82 (dt, *J* = 7.6, 0.9 Hz, 1H), 7.32 – 7.23 (m, 5H), 6.98 – 6.81 (m, 1H), 5.42 (d, *J* = 8.6 Hz, 1H), 4.44 (dt, *J* = 8.7, 3.0 Hz, 1H), 4.24 – 4.09 (m, 1H), 4.06 – 3.91 (m, 3H), 3.76 (d, *J* = 0.6 Hz, 3H), 3.45 (dd, *J* = 10.7, 4.8 Hz, 3H), 3.06 (td, *J* = 7.1, 3.1 Hz, 2H), 1.45 (d, *J* = 2.3 Hz, 10H).

^{13}C NMR (101 MHz, $CDCl_3$) δ 170.71, 155.46, 140.70, 139.72, 130.68, 130.66, 128.59, 128.47, 100.76, 80.23, 52.72, 42.32, 42.28, 31.09, 28.49.

^{31}P NMR (162 MHz, $CDCl_3$) δ 139.42, 139.39.

HRMS (ESI-TOF) m/z: $[M+Na]^+$ calcd. for $C_{18}H_{27}INNaO_7P$, 550.0425; found, 550.0462.

3. Experimental Procedures

3.1 General procedure for deoxygenative Giese reaction

An oven-dried 20 mL vial equipped with a magnetic stir bar was charged with Phosphite-Ser **3** (100.2 mg, 0.19 mmol), PC **6** (3 mol%), NHC-BH_3 (5.0 equiv), and HCO_2K (2.0 equiv). After the vial was vacuumed and refilled with nitrogen three times, separately degassed 20:1 DMSO/H_2O (0.025 M), and radical acceptor (4.0 equiv) were added via syringe. The vial was sealed with parafilm and stirred at 1800 rpm and irradiated under 395 nm LED modules at 100% intensity with the maxed fan in a PennPhD Photoreactor M2 for 24 h. The reaction was quenched with saturated NaCl solution (30 mL) and extracted with ethyl acetate (3 × 30 mL). The combined organic layers were washed with brine, dried over Na_2SO_4, filtered, and concentrated under vacuum. The crude residue was purified by flash column chromatography to give the product.

3.2 General procedure for peptide synthesis

General protocol for solid phase peptide synthesis (SPPS).

Method A: **Peptide Elongation.** Rink Amide resin (500 mg, 0.25 mmol) was swollen in DMF for 30 min and then washed with DCM (3 × 6 mL) and DMF (3 × 6 mL). The Fmoc protecting group was removed by treatment with 20% piperidine in DMF (6 mL) for 30 min at room temperature. Fmoc-protected amino acids (5.0 equiv) were pre-activated with HATU (5.0 equiv) and DIPEA (5.0 equiv) in DMF (6 mL) and coupled to the resin for 30 min. Coupling and deprotection cycles were repeated for each amino acid. After each step, the resin was washed with DCM (3 × 6 mL), and DMF (3 × 6 mL).

Method B: N-Terminal Acetylation. After the final Fmoc deprotection, the resin was treated with a solution of pyridine (5.0 equiv) and acetic anhydride (5.0 equiv) in DMF (6 mL) and stirred at room temperature for 30 min. The resin was then washed with DCM (3 × 6 mL) and DMF (3 × 6 mL).

Method C: TBS deprotection. The resin was swollen in THF (6 mL) for 30 min. Tetrabutylammonium fluoride (TBAF, 1.0 M in THF, 5.0 equiv) was added, and the mixture was stirred for 90 min. The resin was washed with THF (2 × 6 mL), DCM (2 × 6 mL), and DMF (2 × 6 mL).

Method D: Cleavage from resin/side-chain deprotection. A solution of TFA/water/EDT/TIPS 91:3:3:3 by volume (6 mL) was added to the dry resin. The reaction mixture was shaken at room temperature for 2-3 h. The resin was filtered and washed with DCM (4 × 6 mL) and MeOH (4 × 6 mL). The combined filtrate was concentrated under vacuum to reduce the total volume. The solution was then precipitated with cold, anhydrous Et_2O. The crude peptide was purified by

preparative reserved-phase HPLC (Luna® C18 column (250×21.2mm, 10 μm) flow rate = 10.0 mL/min, gradient 5-90% acetonitrile in water containing 0.1% TFA over 30 mins). Fractions containing the product were combined, flash frozen in liquid nitrogen, and lyophilized to give the product as a white powder.

3.3 General procedure for the solution phase peptide modification

Purified peptide (10.0 mg) and 5-methyltetrazole (30.0 equiv) were dissolved in anhydrous DMF (0.2 mL) and degassed under an argon atmosphere for 10 mins. 2-iodophenethyl methyl diisopropylphosphoramidite (10.0 equiv) was added via syringe, and the reaction mixture was stirred at room temperature for 2 h. Monitoring the reaction with LCMS, and then concentrating under vacuum to minimize the total volume. The crude product was precipitated with cold, anhydrous diethyl ether and stored at -20 °C overnight. The resulting precipitate was centrifuged and directly used for the next steps.

An oven-dried 4 mL vial equipped with a magnetic stir bar was charged with Phosphite-peptide (1.0 equiv), PC **6** (6 mol%), NHC-BH_3 (10.0 equiv), and HCO_2K (4.0 equiv). After the vial was vacuumed and refilled with nitrogen gas three times, separately degassed 20:1 DMSO/H_2O (0.01 M), and radical acceptor (8.0 equiv) was added via syringe. The vial was sealed with parafilm and stirred at 1800 rpm and irradiated under 395 nm LED modules at 100% intensity with the maxed fan in a PennPhD Photoreactor M2 for 20 h. Monitoring the reaction with HPLC, DMSO was removed by nitrogen stream, the reaction was diluted with MeOH and purified with prep-HPLC.

3.4 General procedure for solid phase peptide modification

After TBS deprotection, the resin was suspended in DMF followed by the addition of 5-methyltetrazole (10.0 equiv). The mixture was stirred under a nitrogen atmosphere for 10 min. 2-iodophenethyl methyl diisopropylphosphoramidite (5.0 equiv) was then added and the reaction mixture was stirred at room temperature for 4 h. The resin was washed with DCM (2 × 2 mL) and DMSO (2 × 2 mL) and then resuspended in DMSO (0.03 M). To the suspension, PC **6** (6 mol%), NHC-BH_3 (10.0 equiv), HCO_2K (4.0 equiv), and radical acceptor (8.0 equiv) and H_2O were added via syringe under nitrogen. After sealing with parafilm, the vial was stirred at 600 rpm and irradiated under 395 nm LED modules at 100% intensity with the maxed fan in a PennPhD Photoreactor M2 for 20 h. After the reaction, the resin was washed with DCM (3 × 4 mL) and DMF (3 × 4 mL), then peptides were cleaved from resin and side-chain deprotected using **Method D**. The crude product was analyzed by LC-MS and further purified via prep-HPLC.

4. Reaction Optimization and Control Experiments

All the optimization and control experiments were performed on a 0.04 mmol scale with the same experimental procedure **3.1**.

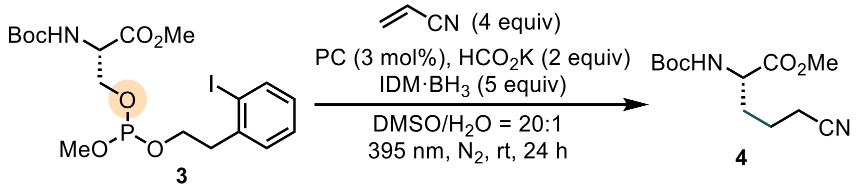

Table S1. Evaluation of different photocatalysts

Photocatalysts	Yield (%)
PTH	72
$Ir(ppy)_3$	55
4CzIPN	0
$[Mes\text{-}Acr]^+ClO_4^-$	0
PC **2**	45

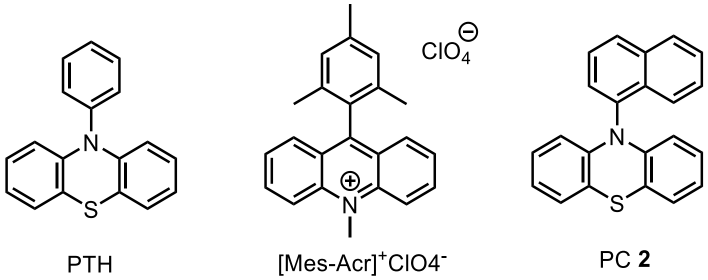

Figure S1. Different photocatalyst screening.

Table S2. Evaluation of different H donors

H donors	Yield (%)
$DMAP\text{-}BH_3$	15
$DiMe\text{-}Tri\text{-}BH_3$	38
$Dipp\text{-}Imd\text{-}BH_3$	30
TTMSS	22
γ-Terpinene	21
1,4-Cyclohexadiene	59

Table S3. Evaluation of different solvents

Solvents	Yield (%)
DMF	68
HMPA	45
DMA	67
Acetonitrile	40

Table S4. Evaluation of different reductants

Reductants	Yield (%)
HCO_2NBu_4	60
$HCO_2Li\cdot H_2O$	71
HCO_2Na	68
$HCO_2Cs\cdot H_2O$	67
HCO_2NH_4	65
NBu_3	71
Et_3SiH	50

Table S5. Control experiments

Deviation from standard	Yield (%)
None	87
Under air	46
No NHC-BH_3	23
No PC	0
No HCO_2K	30
No H_2O	28
No light	0
450 nm	5

Competition experiments between Serine and nucleophilic amino acids

General Procedure

Serine amino acid (20.0 mg, 91.2 µmol, 1.0 equiv), nucleophilic amino acid **X** (91.2 µmol, 1.0 equiv), 1,3,5-trimethoxybenzene (15.3 mg, 91.2 µmol, 1.0 equiv) and 5-methyltetrazole (46.0 mg, 547 µmol) were dissolved in acetonitrile (1 ml, 0.1 M) in a 4 mL vial. 2-iodophenethyl methyl diisopropylphosphoramidite **1** (71.7 mg, 182 µmol) was added, the reaction mixture was stirred for 60 min under a nitrogen atmosphere. (For Lys, 5-methyltetrazole (76.7 mg, 912 µmol) and 2-iodophenethyl methyl diisopropylphosphoramidite (26.9 mg, 91.2 µmol) were used). The crude reaction mixture was analyzed by ^{31}P NMR and GC to calculate the yield, and the solvent was removed under vacuum and directly used to the next steps.

PC **6** (3mol%), NHC-BH_3 (5.0 equiv), and HCO_2K (2.0 equiv) were added into the crude reaction mixture. After the vial was vacuumed and refilled with nitrogen three times, separately degassed 20:1 DMSO/H_2O (0.025 M), and acrylonitrile (4.0 equiv) were added via syringe. The vial was sealed with parafilm and stirred at 1800 rpm and irradiated under 395 nm LED modules at 100% intensity with the maxed fan in a PennPhD Photoreactor M2 for 18 h. The crude reaction mixture was analyzed by GC to calculate the yield.

Table S6. Competition experiments

Entry	Serine	X (1 equiv)	**1** (equiv)	Tetrazole (equiv)	Photoredox Condition (equiv)
1	1 equiv	Thr	2	6	×2
2	1 equiv	Tyr	2	6	×2
3	1 equiv	Lys	1	3	×1
4	1 equiv	Cys	2	6	×2

5. Experimental Data for Deoxygenative Giese Reaction of Serine

Methyl (S)-2-((tert-butoxycarbonyl)amino)-5-cyanopentanoate (4)[7]

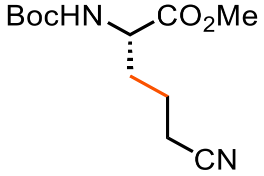

Following the general procedure **3.1**, phosphite-Ser **3** (0.19 mmol, 1.0 equiv), acrylonitrile (40.3 mg, 4.0 equiv), PC (2.5 mg, 3 mol%), NHC-BH_3 (106.0 mg, 5.0 equiv), and HCO_2K (32.0 mg, 2.0 equiv) were used to afford the product as a colorless oil **4** (36 mg, 0.14 mmol, 73% yield).

^{1}H NMR (400 MHz, $CDCl_3$) δ 5.07 (d, J = 8.2 Hz, 1H), 4.34 (s, 1H), 3.77 (s, 3H), 2.41 (t, J = 6.7 Hz, 2H), 2.07 – 1.92 (m, 1H), 1.84 – 1.66 (m, 3H), 1.45 (s, 9H).

Methyl (S)-2-((tert-butoxycarbonyl)amino)-5-(diethoxyphosphoryl)pentanoate (15)

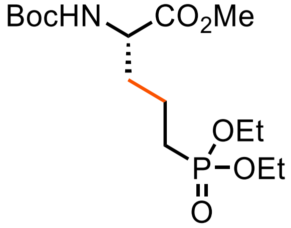

Following the general procedure **3.1**, phosphite-Ser **3** (0.19 mmol, 1.0 equiv), diethyl vinylphosphonate (125 mg, 4.0 equiv), PC (2.5 mg, 3 mol%), NHC-BH_3 (106.0 mg, 5.0 equiv), and HCO_2K (32.0 mg, 2.0 equiv) were used to afford the product as a colorless oil **15** (53.0 mg, 0.14 mmol, 76% yield).

^{1}H NMR (400 MHz, $CDCl_3$) δ 5.08 (d, J = 8.5 Hz, 1H), 4.31 (q, J = 6.6 Hz, 1H), 4.17 – 3.96 (m, 4H), 3.74 (s, 3H), 1.98 – 1.61 (m, 6H), 1.44 (d, J = 1.0 Hz, 9H), 1.32 (t, J = 7.1 Hz, 6H).

^{13}C NMR (101 MHz, $CDCl_3$) δ 172.98, 155.35, 79.95, 61.58, 61.56, 61.52, 61.50, 52.34, 33.48, 33.31, 28.29, 18.66, 18.62, 16.48, 16.42.

^{31}P NMR (162 MHz, $CDCl_3$) δ 31.27.

HRMS (ESI-TOF) m/z: $[M+K]^+$ calcd. for $C_{15}H_{30}KNO_7P$, 406.1391; found, 406.1367.

Methyl (S)-2-((tert-butoxycarbonyl)amino)-6-(dimethylamino)-6-oxohexanoate (16)

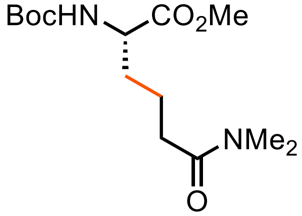

Following the general procedure **3.1**, phosphite-Ser **3** (0.19 mmol, 1.0 equiv), N,N-dimethylacrylamide (75.3 mg, 4.0 equiv), PC (2.5 mg, 3 mol%), NHC-BH_3 (106.0 mg, 5.0 equiv), and HCO_2K (32.0 mg, 2.0 equiv) were used to afford the product as a colorless oil **16** (37.0 mg, 0.12 mmol, 65% yield).

^{1}H NMR (600 MHz, $CDCl_3$) δ 5.14 (d, J = 7.9 Hz, 1H), 4.41 – 4.18 (m, 1H), 3.87 – 3.63 (m, 3H), 2.96 (dd, J = 30.0, 3.9 Hz, 6H), 2.48 – 2.17 (m, 2H), 1.91 – 1.80 (m, 1H), 1.79 – 1.62 (m, 3H), 1.51 – 1.31 (s, 9H).

^{13}C NMR (151 MHz, $CDCl_3$) δ 173.35, 172.40, 155.61, 79.97, 53.35, 52.40, 37.31, 35.53, 32.59, 32.32, 28.47, 20.91.

HRMS (ESI-TOF) m/z: $[M+H]^+$ calcd. for $C_{14}H_{28}N_2O_5$, 303.1914; found, 303.1915.

Methyl (S)-6-amino-2-((tert-butoxycarbonyl)amino)-6-oxohexanoate (17)

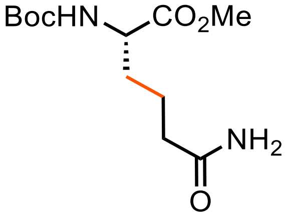

Following the general procedure **3.1**, phosphite-Ser **3** (0.19 mmol, 1.0 equiv), acrylamide (54 mg, 4.0 equiv), PC (2.5 mg, 3 mol%), NHC-BH_3 (106.0 mg, 5.0 equiv), and HCO_2K (32.0 mg, 2.0 equiv) were used to afford the product as a colorless oil **17** (27.0 mg, 97 μmol, 51% yield).

^{1}H NMR (400 MHz, $CDCl_3$) δ 5.55 (d, J = 65.5 Hz, 2H), 5.15 (d, J = 8.4 Hz, 1H), 4.31 (d, J = 7.8 Hz, 1H), 3.74 (s, 3H), 2.28 (dh, J = 28.2, 6.8, 5.8 Hz, 2H), 1.84 (d, J = 9.7 Hz, 1H), 1.70 (h, J = 7.9 Hz, 3H), 1.44 (s, 9H).

^{13}C NMR (101 MHz, $CDCl_3$) δ 174.82, 173.08, 155.58, 80.06, 52.74, 52.41, 32.25, 29.71, 28.33, 21.31.

HRMS (ESI-TOF) m/z: $[M+H]^+$ calcd. for $C_{12}H_{23}N_2O_5$, 275.1601; found, 275.1602.

6-benzyl 1-methyl (S)-2-((tert-butoxycarbonyl)amino)hexanedioate (18)

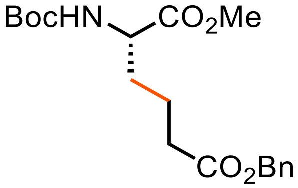

Following the general procedure **3.1**, phosphite-Ser **3** (0.19 mmol, 1.0 equiv), benzyl acrylate (123 mg, 4.0 equiv), PC (2.5 mg, 3 mol%), NHC-BH_3 (106.0 mg, 5.0 equiv), and HCO_2K (32.0 mg, 2.0 equiv) were used to afford the product as a colorless oil **15** (37.0 mg, 0.10 mmol, 53% yield).

^{1}H NMR (400 MHz, $CDCl_3$) δ 7.41 – 7.29 (m, 5H), 5.11 (s, 2H), 5.02 (d, J = 8.4 Hz, 1H), 4.39 – 4.22 (m, 1H), 3.73 (s, 3H), 2.39 (td, J = 7.2, 3.0 Hz, 2H), 1.84 (tt, J = 10.5, 3.9 Hz, 1H), 1.69 (qt, J = 11.7, 5.5 Hz, 3H), 1.44 (s, 9H).
^{13}C NMR (101 MHz, $CDCl_3$) δ 173.06, 172.87, 155.37, 135.92, 128.59, 128.28, 128.24, 79.99, 66.30, 53.10, 52.34, 33.60, 32.08, 28.32, 20.75.
HRMS (ESI-TOF) m/z: $[M+Na]^+$ calcd. for $C_{19}H_{27}NaNO_6$, 388.1731; found, 388.1735.

6-(*tert*-butyl) 1-methyl (*S*)-2-((*tert*-butoxycarbonyl)amino)hexanedioate (19)

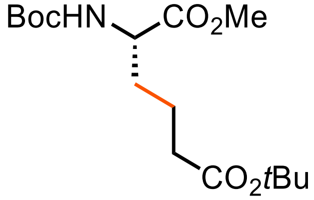

Following the general procedure **3.1**, phosphite-Ser **3** (0.19 mmol, 1.0 equiv), tert-butyl acrylate (125 mg, 4.0 equiv), PC (2.5 mg, 3 mol%), NHC-BH_3 (106.0 mg, 5.0 equiv), and HCO_2K (32.0 mg, 2.0 equiv) were used to afford the product as a colorless oil **15** (30.0 mg, 91 μmol, 48% yield).
^{1}H NMR (400 MHz, $CDCl_3$) δ 5.04 (d, J = 8.4 Hz, 1H), 4.43 – 4.17 (m, 1H), 3.74 (s, 3H), 2.24 (td, J = 7.3, 1.8 Hz, 2H), 1.82 (tdd, J = 11.0, 7.9, 4.3 Hz, 1H), 1.71 – 1.62 (m, 3H), 1.44 (d, J = 1.7 Hz, 18H).
^{13}C NMR (101 MHz, $CDCl_3$) δ 173.29, 172.57, 155.50, 80.50, 80.05, 53.31, 52.44, 34.99, 32.15, 28.46, 28.24, 21.02.
HRMS (ESI-TOF) m/z: $[M+Na]^+$ calcd. for $C_{16}H_{29}NaNO_6$, 354.1887; found, 354.1817.

1-methyl 6-(prop-2-yn-1-yl) (S)-2-((tert-butoxycarbonyl)amino)hexanedioate (20)

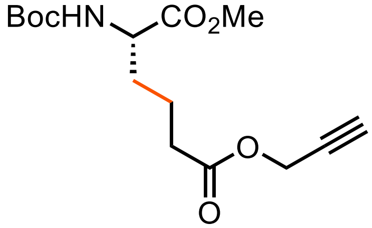

Following the general procedure **3.1**, phosphite-Ser **3** (0.19 mmol, 1.0 equiv), prop-2-yn-1-yl acrylate (83.7 mg, 4.0 equiv), PC (2.5 mg, 3 mol%), NHC-BH_3 (106.0 mg, 5.0 equiv), and HCO_2K (32.0 mg, 2.0 equiv) were used to afford the product as a colorless oil **15** (23.0 mg, 72 μmol, 38% yield).
^{1}H NMR (400 MHz, $CDCl_3$) δ 5.04 (d, J = 8.4 Hz, 1H), 4.67 (d, J = 2.5 Hz, 2H), 4.36 – 4.23 (m, 1H), 3.74 (s, 3H), 2.47 (t, J = 2.5 Hz, 1H), 2.39 (td, J = 7.2, 3.0 Hz, 2H), 1.91 – 1.80 (m, 1H), 1.76 – 1.62 (m, 3H), 1.44 (s, 9H).

^{13}C NMR (101 MHz, $CDCl_3$) δ 173.01, 172.19, 155.35, 79.99, 77.62, 77.42, 74.89, 53.06, 52.36, 51.93, 33.27, 32.00, 28.31, 20.61.
HRMS (ESI-TOF) m/z: $[M+Na]^+$ calcd. for $C_{15}H_{23}NaNO_6$, 336.1418; found, 336.1405.

6-((1-(1-(3',6'-dihydroxy-3-oxo-3H-spiro[isobenzofuran-1,9'-xanthen]-6-yl)-1-oxo-5,8,11-trioxa-2-azatridecan-13-yl)-1H-1,2,3-triazol-4-yl)methyl) 1-methyl (S)-2-((tert-butoxy carbonyl)amino)hexanedioate (21)

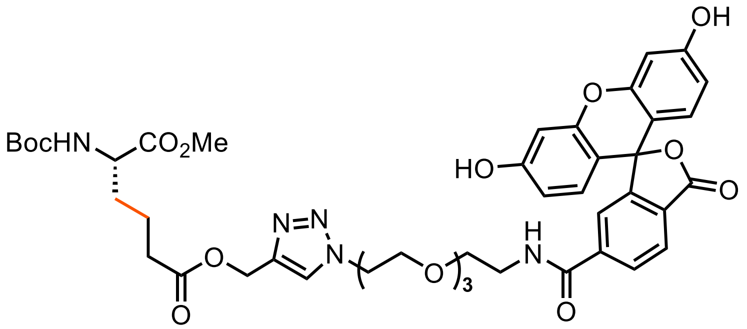

Compound **20** (20.0 mg, 64 μmol) was dissolved in DMF in a flamed-dried 20 mL vial equipped with a magnetic stir bar. CuI (1.2 mg, 0.1 equiv), Tris(benzyltriazolylmethyl)amine (TBTA) (1.7 mg, 0.05 equiv) and Fluorescein-$(PEG)_3$-N_3 (36.8 mg, 64 μmol) was added to the reacion mixture. After stirring for 1 hour, the reaction mixture was concentrated in vacuo and purified by flash column chromatography to afford **21** as a yellow oil in 87% yield.
^{1}H NMR (400 MHz, MeOD) δ 8.12 (d, J = 3.4 Hz, 3H), 7.98 (s, 1H), 7.64 (s, 1H), 6.70 (d, J = 2.4 Hz, 2H), 6.62 – 6.49 (m, 4H), 5.13 (s, 2H), 4.55 – 4.38 (m, 2H), 4.11 – 4.00 (m, 1H), 3.83 – 3.74 (m, 2H), 3.68 (s, 3H), 3.61 – 3.43 (m, 12H), 2.33 (t, J = 6.8 Hz, 2H), 1.82 – 1.50 (m, 3H), 1.41 (s, 9H).
^{13}C NMR (101 MHz, MeOD) δ 174.89, 174.59, 170.72, 168.39, 168.31, 165.03, 161.28, 158.10, 154.35, 153.92, 143.71, 142.28, 142.23, 130.51, 130.27, 126.57, 126.26, 124.00, 113.80, 110.81, 103.64, 80.81, 71.31, 71.25, 71.21, 71.19, 71.00, 70.19, 70.09, 58.16, 54.78, 54.68, 52.79, 51.33, 41.02, 34.06, 31.77, 31.73, 28.67, 22.19.
HRMS (ESI-TOF) m/z: $[M+Na]^+$ calcd. for $C_{44}H_{51}NaN_5O_{15}$, 912.3274; found, 912.3272.

1-methyl 6-((1-(13-oxo-17-((3aS,4S,6aR)-2-oxohexahydro-1H-thieno[3,4-d]imidazol-4-yl)-3,6,9-trioxa-12-azaheptadecyl)-1H-1,2,3-triazol-4-yl)methyl) (S)-2-((tert-butoxycarbonyl) amino)hexanedioate (22)

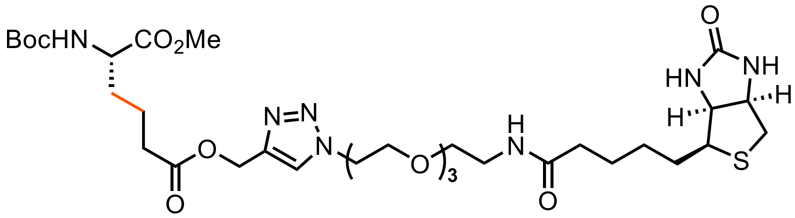

Following the general procedure **3.1**, phosphite-Ser **3** (45.5 μmol, 1.0 equiv), (1-(13-oxo-17-((3aS,4S,6aR)-2-oxohexahydro-1H-thieno[3,4-d]imidazol-4-yl)-3,6,9-trioxa-12-azaheptadecyl)-1H-1,2,3-triazol-4-yl)methyl acrylate (101 mg, 4.0 equiv), PC (0.6 mg, 3 mol%), NHC-BH_3 (25.5 mg, 5.0 equiv), and HCO_2K (32.0 mg, 2.0 equiv) were used to afford the product as a colorless oil **15** (21 mg, 27 μmol, 60% yield).

^{1}H NMR (400 MHz, MeOD) δ 8.19 (s, 1H), 8.07 (s, 1H), 7.98 (q, *J* = 8.3, 7.0 Hz, 1H), 5.21 (d, *J* = 1.6 Hz, 2H), 4.50 (dd, *J* = 7.9, 5.0 Hz, 1H), 4.32 (dd, *J* = 8.0, 4.5 Hz, 1H), 4.13 – 4.03 (m, 1H), 3.91 (t, *J* = 4.9 Hz, 2H), 3.71 (s, 3H), 3.66 – 3.59 (m, 8H), 3.54 (t, *J* = 5.4 Hz, 2H), 3.37 (q, *J* = 5.5 Hz, 2H), 3.21 (dt, *J* = 9.6, 5.1 Hz, 1H), 2.93 (ddd, *J* = 12.7, 4.9, 1.6 Hz, 1H), 2.77 – 2.65 (m, 1H), 2.39 (t, *J* = 6.9 Hz, 2H), 2.22 (t, *J* = 7.3 Hz, 2H), 1.68 (ddt, *J* = 24.4, 17.0, 8.2 Hz, 4H), 1.45 (s, 9H).

^{13}C NMR (101 MHz, MeOD) δ 174.72, 173.33, 172.98, 164.70, 156.74, 142.48, 128.66, 79.21, 70.15, 70.09, 70.02, 69.86, 69.19, 68.90, 61.97, 60.23, 56.80, 55.59, 53.36, 51.24, 50.05, 39.65, 38.94, 35.34, 32.75, 30.46, 28.36, 28.10, 27.31, 25.44, 20.95.

HRMS (ESI-TOF) m/z: $[M+K]^+$ calcd. for $C_{33}H_{55}KN_7O_{11}S$, 796.3312; found, 796.3257.

(S)-5-benzamido-6-methoxy-6-oxohexanoic acid (23)

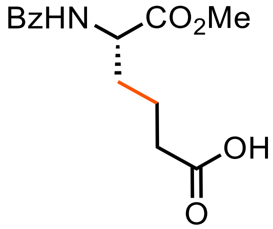

Following the general procedure **3.1**, phosphite-Ser **3** (0.19 mmol, 1.0 equiv), Acrylic acid (54.8 mg, 4.0 equiv), PC (2.5 mg, 3 mol%), NHC-BH_3 (106.0 mg, 5.0 equiv), and HCO_2K (32.0 mg, 2.0 equiv) were used to afford the product as a colorless oil **23** (22.0 mg, 78.0 μmol, 41% yield).

^{1}H NMR (400 MHz, $CDCl_3$) δ 7.89 – 7.73 (m, 2H), 7.56 – 7.49 (m, 1H), 7.48 – 7.41 (m, 2H), 6.81 (d, *J* = 7.7 Hz, 1H), 4.95 – 4.70 (m, 1H), 3.80 (s, 3H), 2.51 – 2.32 (m, 2H), 2.10 – 1.98 (m, 1H), 1.91 – 1.70 (m, 3H).

^{13}C NMR (101 MHz, $CDCl_3$) δ 176.08, 173.00, 167.36, 133.88, 132.03, 128.80, 127.24, 52.80, 52.37, 32.90, 32.00, 20.55.
HRMS (ESI-TOF) m/z: $[M+Na]^+$ calcd. for $C_{14}H_{17}NNaO_5$, 302.0999; found, 302.1003.

Methyl (S)-2-((tert-butoxycarbonyl)amino)-5-(pyridin-2-yl)pentanoate (24)

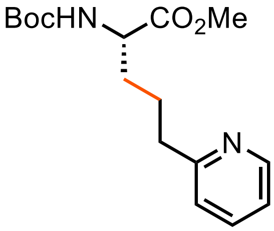

Following the general procedure **3.1**, phosphite-Ser **3** (0.19 mmol, 1.0 equiv), 2-vinylpyridine (80.0 mg, 4.0 equiv), PC (2.5 mg, 3 mol%), NHC-BH_3 (106.0 mg, 5.0 equiv), and HCO_2K (32.0 mg, 2.0 equiv) were used to afford the product as a colorless oil **24** (17.0 mg, 53.0 μmol, 28% yield).
^{1}H NMR (400 MHz, $CDCl_3$) δ 8.53 (d, *J* = 5.0 Hz, 1H), 7.61 (t, *J* = 7.8 Hz, 1H), 7.19 – 7.07 (m, 2H), 5.13 (d, *J* = 7.4 Hz, 1H), 4.33 (q, *J* = 6.9 Hz, 1H), 3.72 (s, 3H), 2.82 (q, *J* = 6.7 Hz, 2H), 1.82 (qd, *J* = 14.1, 13.1, 6.1 Hz, 3H), 1.71 (dt, *J* = 14.6, 7.2 Hz, 1H), 1.44 (s, 9H).
^{13}C NMR (101 MHz, $CDCl_3$) δ 173.41, 161.32, 155.56, 149.17, 136.82, 123.12, 121.40, 79.99, 53.48, 52.41, 37.58, 32.33, 28.51, 28.47, 25.56.
HRMS (ESI-TOF) m/z: $[M]^+$ calcd. for $C_{16}H_{24}N_2O_4$, 308.1731; found, 308.1732.

Dimethyl (5S)-2-(bis(tert-butoxycarbonyl)amino)-5-((tert-butoxycarbonyl)amino) hexane - dioate (25)

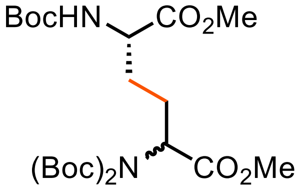

Following the general procedure **3.1**, phosphite-Ser **3** (0.19 mmol, 1.0 equiv), methyl 2-(bis(tert-butoxycarbonyl)amino)acrylate (229 mg, 4.0 equiv), PC (2.5 mg, 3 mol%), NHC-BH_3 (106.0 mg, 5.0 equiv), and HCO_2K (32.0 mg, 2.0 equiv) were used to afford the product as a colorless oil **25** (56.0 mg, 0.11 mmol, 58% yield)(d.r. = 1:1).
^{1}H NMR (400 MHz, $CDCl_3$) δ 5.13 – 4.70 (m, 2H), 4.53 – 4.21 (m, 1H), 3.76 – 3.66 (m, 6H), 2.16 (dtd, *J* = 13.5, 11.4, 5.2 Hz, 1H), 1.99 – 1.69 (m, 3H), 1.49 (d, *J* = 1.2 Hz, 18H), 1.43 (s, 9H).
^{13}C NMR (101 MHz, $CDCl_3$) δ 172.95, 170.92, 155.31, 151.98, 151.92, 83.36, 83.31, 83.18, 83.16, 79.90, 57.67, 57.41, 52.33, 52.29, 52.24, 52.22, 28.30, 27.97, 27.95, 26.04, 25.50.
HRMS (ESI-TOF) m/z: $[M+Na]^+$ calcd. for $C_{23}H_{40}N_2NaO_{10}$, 527.2575; found, 527.2541.

Methyl (2S)-2-((tert-butoxycarbonyl)amino)-6,6,6-trifluoro-5-phenylhexanoate (26)

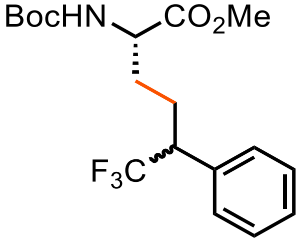

Following the general procedure **3.1**, phosphite-Ser **3** (0.19 mmol, 1.0 equiv), (3,3,3-trifluoroprop-1-en-2-yl)benzene (131 mg, 4.0 equiv), PC (2.5 mg, 3 mol%), NHC-BH_3 (106.0 mg, 5.0 equiv), and HCO_2K (32.0 mg, 2.0 equiv) were used to afford the product as a colorless oil **26** (40.0 mg, 0.11 mmol, 56% yield)(d.r. = 1.3:1).

^{1}H NMR (400 MHz, $CDCl_3$) δ 7.47 – 7.12 (m, 5H), 5.00 (dd, *J* = 16.3, 8.5 Hz, 1H), 4.30 (dt, *J* = 14.0, 5.5 Hz, 1H), 3.69 (d, *J* = 16.9 Hz, 3H), 3.25 (ht, *J* = 9.2, 6.6, 4.3 Hz, 1H), 2.10 – 1.91 (m, 2H), 1.79 – 1.49 (m, 2H), 1.43 (d, *J* = 8.4 Hz, 9H).

^{13}C NMR (101 MHz, $CDCl_3$) δ 172.81, 172.74, 155.40, 155.20, 134.13, 134.02, 128.96, 128.91, 128.82, 128.58, 128.38, 128.09, 127.49, 125.30, 80.17, 80.10, 53.05, 52.66, 52.40, 52.35, 49.81, 49.55, 30.20, 28.27, 24.77, 24.75.

^{19}F NMR (471 MHz, $CDCl_3$) δ -69.79 (d, *J* = 63.5 Hz).

HRMS (ESI-TOF) m/z: $[M+Na]^+$ calcd. for $C_{18}H_{24}F_3NNaO_4$, 399.1583; found, 399.1524.

Tert-butyl 3-((S)-3-((tert-butoxycarbonyl)amino)-4-methoxy-4-oxobutyl)pyrrolidine-1-carboxylate (27)

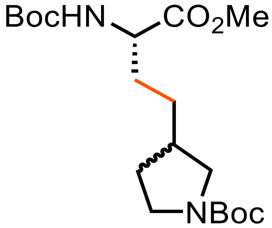

Following the general procedure **3.1**, phosphite-Ser **3** (0.19 mmol, 1.0 equiv), tert-butyl 3-methylenepyrrolidine-1-carboxylate (139 mg, 4.0 equiv), PC (2.5 mg, 3 mol%), NHC-BH_3 (106.0 mg, 5.0 equiv), and HCO_2K (32.0 mg, 2.0 equiv) were used to afford the product as a colorless oil **27** (31.0 mg, 80.0 μmol, 42% yield)(d.r. = 1:1).

^{1}H NMR (400 MHz, $CDCl_3$) δ 5.01 (d, *J* = 5.3 Hz, 1H), 4.31 (d, *J* = 8.9 Hz, 1H), 3.74 (d, *J* = 1.7 Hz, 3H), 3.59 – 3.34 (m, 2H), 3.24 (p, *J* = 10.0 Hz, 1H), 2.93 – 2.67 (m, 1H), 2.15 – 1.58 (m, 4H), 1.45 (d, *J* = 3.3 Hz, 18H), 1.42 – 1.14 (m, 2H).

^{13}C NMR (101 MHz, $CDCl_3$) δ 173.15, 155.36, 154.57, 80.05, 79.09, 53.33, 52.37, 51.53, 51.08, 45.69, 45.35, 38.71, 37.80, 31.46, 28.78, 28.56, 28.37, 28.32, 22.67.

HRMS (ESI-TOF) m/z: $[M+Na]^+$ calcd. for $C_{19}H_{34}NaN_2O_6$, 409.2309; found, 409.2274.

Methyl (S)-2-((tert-butoxycarbonyl)amino)-5-(dimethyl(phenyl)silyl)pentanoate (28)

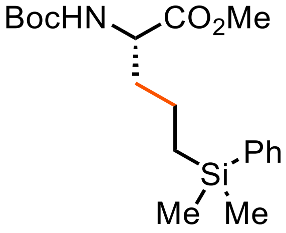

Following the general procedure **3.1**, phosphite-Ser **3** (0.19 mmol, 1.0 equiv), dimethyl(phenyl)(vinyl)silane (123 mg, 4.0 equiv), PC (2.5 mg, 3 mol%), NHC-BH_3 (106.0 mg, 5.0 equiv), and HCO_2K (32.0 mg, 2.0 equiv) were used to afford the product as a colorless oil **28** (35.0 mg, 97 μmol, 51% yield).

^{1}H NMR (400 MHz, $CDCl_3$) δ 7.52 – 7.46 (m, 2H), 7.39 – 7.30 (m, 3H), 4.91 (d, *J* = 8.6 Hz, 1H), 4.29 (q, *J* = 7.6 Hz, 1H), 3.69 (s, 3H), 1.78 (dq, *J* = 11.4, 6.6, 5.8 Hz, 1H), 1.67 – 1.58 (m, 1H), 1.43 (s, 9H), 1.40 – 1.31 (m, 2H), 0.85 – 0.65 (m, 2H), 0.25 (d, *J* = 0.7 Hz, 6H).

^{13}C NMR (101 MHz, $CDCl_3$) δ 173.58, 155.37, 139.08, 133.54, 128.90, 127.78, 79.83, 53.06, 52.15, 36.49, 28.32, 19.85, 15.26, -3.08, -3.13.

HRMS (ESI-TOF) m/z: $[M]^+$ calcd. for $C_{18}H_{31}NO_4Si$, 353.1994; found, 353.2022.

methyl (S)-2-((tert-butoxycarbonyl)amino)-5-(4,4,5,5-tetramethyl-1,3,2-dioxaborolan-2-yl)pentanoate (29)

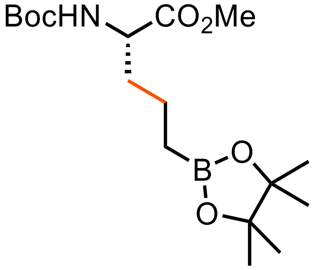

Following the general procedure **3.1**, phosphite-Ser **3** (0.19 mmol, 1.0 equiv), 4,4,5,5-tetramethyl-2-vinyl-1,3,2-dioxaborolane (117 mg, 4.0 equiv), PC (2.5 mg, 3 mol%), NHC-BH_3 (106.0 mg, 5.0 equiv), and HCO_2K (32.0 mg, 2.0 equiv) were used to afford the product as a colorless oil **29** (31.0 mg, 86.0 μmol, 45% yield).

^{1}H NMR (400 MHz, $CDCl_3$) δ 5.04 (d, *J* = 8.3 Hz, 1H), 4.26 (td, *J* = 7.9, 5.1 Hz, 1H), 3.72 (s, 3H), 1.79 (ddt, *J* = 15.5, 10.7, 5.4 Hz, 1H), 1.69 – 1.56 (m, 1H), 1.44 (s, 9H), 1.23 (d, *J* = 2.9 Hz, 12H), 0.78 (td, *J* = 7.7, 3.5 Hz, 2H).

^{13}C NMR (101 MHz, $CDCl_3$) δ 173.48, 155.41, 83.08, 79.70, 53.50, 52.13, 34.89, 28.34, 24.82, 19.77.

HRMS (ESI-TOF) m/z: $[M+Na]^+$ calcd. for $C_{17}H_{32}BNNaO_6$, 380.2218; found, 380.2204.

Methyl (S)-2-((tert-butoxycarbonyl)amino)-5-hydroxypentanoate (30)[8]

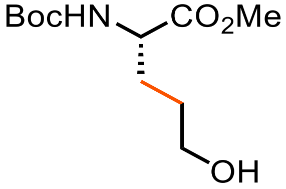

To a solution of **29** (70.0 μmol, 1.0 equiv) in THF:H_2O (2 mL, 1:1(v/v)) was added sodium perborate (54.0 mg, 3.0 equiv). The reaction mixture was stirred for 4 h at room temperature. The reaction was extracted with ethyl acetate three times. The combined organic layers were dried over Na_2SO_4, concentrated in vacuo, and purified by flash column chromatography to afford **30** as a colorless oil in 72% yield.

^{1}H NMR (400 MHz, $CDCl_3$) δ 5.01 (d, *J* = 8.5 Hz, 1H), 4.31 (d, *J* = 8.7 Hz, 1H), 3.74 (d, *J* = 1.3 Hz, 3H), 3.63 (t, *J* = 6.5 Hz, 2H), 1.79 (d, *J* = 8.8 Hz, 1H), 1.68 – 1.55 (m, 3H), 1.45 (d, *J* = 1.3 Hz, 9H).

Methyl (S)-3-(bicyclo[1.1.1]pentan-1-yl)-2-((tert-butoxycarbonyl)amino)propanoate (31)[9]

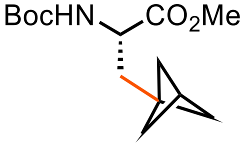

Following the general procedure **3.1**, phosphite-Ser **3** (0.19 mmol, 1.0 equiv), [1.1.1]Propellane in Et_2O (4.0 equiv), PC (2.5 mg, 3 mol%), NHC-BH_3 (106.0 mg, 5.0 equiv), and HCO_2K (32.0 mg, 2.0 equiv) were used to afford the product as a colorless oil **32** (23.0 mg, 86.0 μmol, 45% yield).

^{1}H NMR (400 MHz, $CDCl_3$) δ 4.86 (d, J = 8.7 Hz, 1H), 4.24 (dt, J = 8.2, 4.1 Hz, 1H), 3.66 (s, 3H), 2.38 (s, 1H), 1.91 (dd, J = 14.7, 4.6 Hz, 1H), 1.66 (m, 7H), 1.38 (s, 9H).

methyl (2*S*)-2-((*tert*-butoxycarbonyl)amino)-4-(3-methyl-2-oxo-1-phenylindolin-3-yl) butanoate (32)

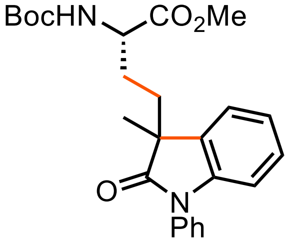

Following the general procedure **3.1**, phosphite-Ser **3** (0.19 mmol, 1.0 equiv), N,N-diphenylmethacrylamide (180 mg, 4.0 equiv), PC (2.5 mg, 3 mol%), NHC-BH_3 (106.0 mg, 5.0 equiv), and HCO_2K (32.0 mg, 2.0 equiv) were used to afford the product as a colorless oil **33** (44.0 mg, 0.10 mmol, 53% yield).

^{1}H NMR (400 MHz, $CDCl_3$) δ 7.52 (t, *J* = 7.6 Hz, 2H), 7.39 (d, *J* = 7.8 Hz, 3H), 7.20 (q, *J* = 7.5 Hz, 2H), 7.10 (t, *J* = 7.5 Hz, 1H), 6.83 (d, *J* = 7.9 Hz, 1H), 4.94 (dm, *J* = 25.6, 8.3, 8.0, 7.3, 0.4 Hz, 1H), 4.21 (q, *J* = 5.6 Hz, 1H), 3.69 (d, *J* = 4.3 Hz, 3H), 2.17 – 1.96 (m, 1H), 1.96 – 1.76 (m, 2H), 1.76 – 1.61 (m, 1H), 1.47 (s, 3H), 1.43 (s, 9H).
^{13}C NMR (101 MHz, $CDCl_3$) δ 179.51, 179.42, 172.83, 155.32, 143.17, 143.09, 134.47, 133.07, 129.60, 128.02, 127.92, 127.90, 126.53, 123.25, 123.15, 122.79, 122.69, 109.47, 79.97, 53.24, 52.38, 52.31, 47.95, 47.86, 44.56, 34.11, 29.72, 28.31, 27.59, 24.35, 24.04, 22.61, 22.39.
HRMS (ESI-TOF) m/z: $[M+K]^+$ calcd. for $C_{20}H_{30}KN_2O_5$, 477.1786; found, 477.1808.

methyl (S,E)-2-((tert-butoxycarbonyl)amino)-5-phenylpent-4-enoate (33)

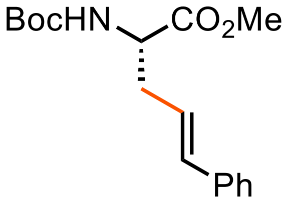

Following the general procedure **3.1**, phosphite-Ser **3** (0.19 mmol, 1.0 equiv), Phenylacetylene (77.5 mg, 4.0 equiv), PC (2.5 mg, 3 mol%), NHC-BH_3 (106.0 mg, 5.0 equiv), and HCO_2K (32.0 mg, 2.0 equiv) were used to afford the product as a colorless oil **33** (27.0 mg, 87.0 μmol, 46% yield)(*Z/E* = 1/5).
^{1}H NMR (400 MHz, $CDCl_3$) δ 7.36 – 7.30 (m, 2H), 7.25 – 7.20 (m, 2H), 6.66 – 6.38 (m, 1H), 6.15 – 5.46 (m, 1H), 5.04 (d, *J* = 8.6 Hz, 1H), 4.42 (q, *J* = 7.1 Hz, 1H), 3.70 (s, 2H), 2.94 – 2.65 (m, 2H), 1.43 (s, 9H).
^{13}C NMR (101 MHz, $CDCl_3$) δ 172.63, 155.25, 136.76, 132.60, 128.67, 128.55, 128.39, 128.30, 127.56, 127.04, 126.28, 125.71, 123.73, 80.00, 53.27, 52.33, 31.41, 28.30.
HRMS (ESI-TOF) m/z: $[M+Na]^+$ calcd. for $C_{17}H_{23}NaNO_4$, 328.1519; found, 328.1522.

diethyl (3-((3a*R*,5*R*,5a*S*,8a*S*,8b*R*)-2,2,7,7-tetramethyltetrahydro-5*H*-bis([1,3]dioxolo)[4,5-*b*:4',5'-*d*]pyran-5-yl)propyl)phosphonate (34)

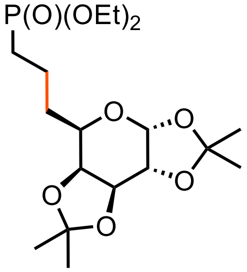

Following the general procedure **3.1**, phosphite-galactose (0.19 mmol, 1.0 equiv), diethyl vinylphosphonate (125 mg, 4.0 equiv), PC (2.5 mg, 3 mol%), NHC-BH_3 (106.0 mg, 5.0 equiv),

and HCO_2K (32.0 mg, 2.0 equiv) were used to afford the product as a colorless oil **34** (41.0 mg, 0.10 mmol, 53% yield).

^{1}H NMR (400 MHz, $CDCl_3$) δ 5.51 (d, *J* = 5.1 Hz, 1H), 4.58 (dd, *J* = 7.9, 2.2 Hz, 1H), 4.28 (dd, *J* = 5.1, 2.3 Hz, 1H), 4.14 – 4.01 (m, 5H), 3.79 – 3.63 (m, 1H), 1.84 – 1.71 (m, 4H), 1.70 – 1.60 (m, 2H), 1.51 (s, 3H), 1.45 (s, 3H), 1.36 – 1.25 (m, 12H).

^{13}C NMR (101 MHz, $CDCl_3$) δ 109.03, 108.29, 96.55, 72.74, 70.92, 70.51, 66.82, 61.48, 61.43, 61.38, 30.91, 30.77, 26.07, 26.00, 25.94, 24.92, 24.82, 24.35, 18.92, 18.88, 16.50, 16.45.

^{31}P NMR (202 MHz, $CDCl_3$) δ 32.03.

HRMS (ESI-TOF) m/z: $[M+K]^+$ calcd. for $C_{18}H_{33}KO_8P$, 447.1545; found, 477.1544.

Ac-Arg-Ser-Phe-Gln-Trp-NH$_2$ (39)

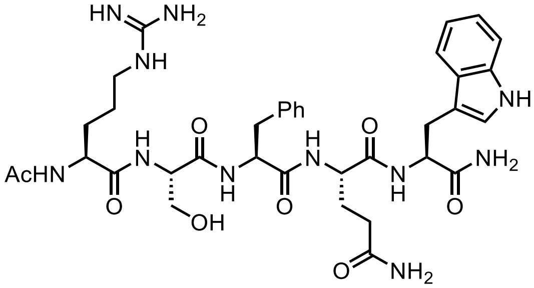

Following general procedure **3.2**, rink amide resin (0.05 mmol) was used to afford the product as a white powder **39** (26 mg, 0.034 mmol, 68% yield).

^{1}H NMR (400 MHz, MeOD) δ 7.63 (dt, J = 7.7, 1.1 Hz, 1H), 7.30 (dt, J = 8.1, 1.0 Hz, 1H), 7.26 – 7.12 (m, 6H), 7.05 (dddd, J = 23.4, 8.1, 7.0, 1.2 Hz, 2H), 4.64 (dd, J = 8.3, 5.6 Hz, 1H), 4.50 (dd, J = 9.1, 4.9 Hz, 1H), 4.33 (dt, J = 7.0, 5.7 Hz, 2H), 4.22 (dd, J = 8.6, 5.6 Hz, 1H), 3.78 (dd, J = 11.0, 5.5 Hz, 1H), 3.69 (dd, J = 11.0, 5.6 Hz, 1H), 3.22 – 3.06 (m, 4H), 2.95 (dd, J = 14.2, 9.2 Hz, 1H), 2.15 (td, J = 7.5, 3.0 Hz, 2H), 1.99 (s, 3H), 1.97 – 1.73 (m, 3H), 1.70 – 1.55 (m, 3H).

^{13}C NMR (101 MHz, MeOD) δ 176.49, 175.17, 172.82, 172.36, 172.29, 171.86, 171.43, 157.18, 136.94, 136.62, 128.91, 128.13, 127.35, 126.44, 123.26, 121.07, 118.49, 118.00, 110.91, 109.59, 61.23, 55.55, 55.44, 53.88, 53.51, 53.01, 40.52, 36.43, 30.99, 28.58, 27.45, 26.92, 24.60, 21.08.

HRMS (ESI-TOF) m/z: $[M+Na]^+$ calcd. for $C_{36}H_{49}NaN_{11}O_8$, 787.3686; found, 787.3455.

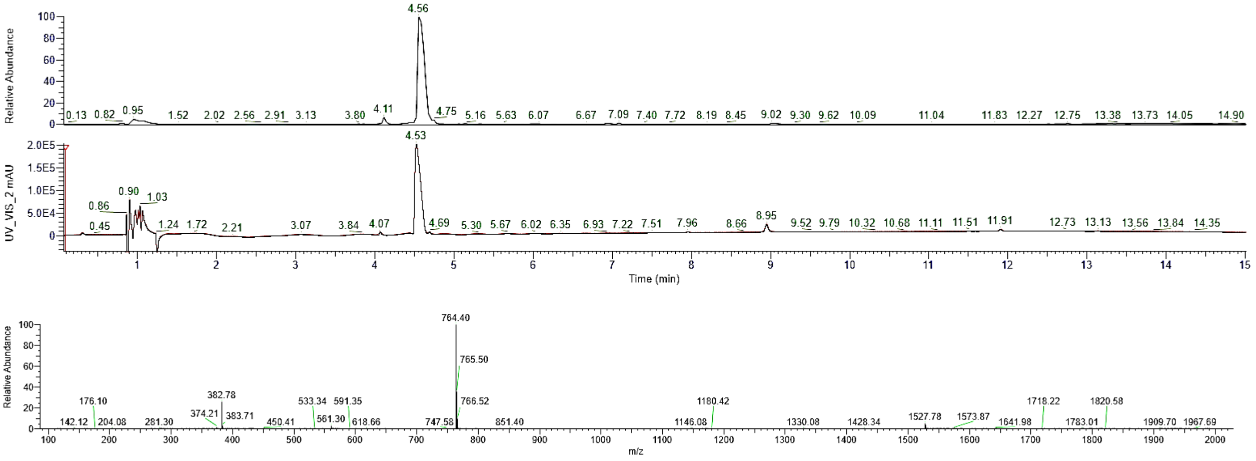

Figure S2. LCMS and ESI-TOF for **39**.

Ac-Arg-Ser-Ala($CH_2CH_2PO(OEt)_2$)-Gln-Trp-NH_2 (37)

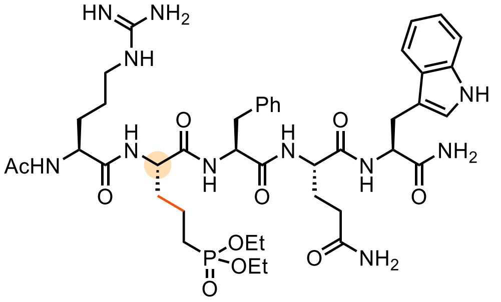

Following general procedure **3.2** and **3.4**, rink amide resin (0.02 mmol) and diethyl vinylphosphonate (8.0 equiv) were used to afford the product as a white powder **37** (5.8 mg, 6.4 μmol, 32% yield).

^{1}H NMR (400 MHz, MeOD) δ 7.62 (d, *J* = 7.8 Hz, 1H), 7.29 (d, *J* = 8.1 Hz, 1H), 7.23 (t, *J* = 7.4 Hz, 2H), 7.20 (d, *J* = 7.7 Hz, 2H), 7.17 (t, *J* = 7.6 Hz, 1H), 7.14 (s, 1H), 7.07 (t, *J* = 7.4 Hz, 1H), 7.02 (t, *J* = 7.4 Hz, 1H), 4.64 (dd, *J* = 8.1, 5.3 Hz, 1H), 4.45 (dd, *J* = 9.5, 5.1 Hz, 1H), 4.30 (p, *J* = 4.8 Hz, 1H), 4.22 (dd, *J* = 8.7, 5.5 Hz, 1H), 4.19 (dd, *J* = 9.4, 5.0 Hz, 1H), 4.09 – 4.02 (m, 4H), 3.20 (q, *J* = 8.7, 8.1 Hz, 2H), 3.17 (t, *J* = 7.4 Hz, 2H), 3.09 (dd, *J* = 14.2, 5.1 Hz, 1H), 2.93 (dd, *J* = 14.1, 9.6 Hz, 1H), 2.18 (t, *J* = 7.6 Hz, 2H), 2.02 – 1.96 (m, 4H), 1.92 (dh, *J* = 14.8, 7.3 Hz, 1H), 1.75 (ddd, *J* = 24.6, 11.7, 6.1 Hz, 3H), 1.66 (dt, *J* = 14.6, 8.4 Hz, 3H), 1.60 (p, *J* = 7.5 Hz, 2H), 1.49 (p, *J* = 6.9 Hz, 2H), 1.32 – 1.28 (m, 6H).

^{13}C NMR (101 MHz, MeOD) δ 173.10, 172.84, 172.39, 157.22, 137.01, 136.62, 129.17, 128.90, 128.14, 127.38, 126.43, 123.20, 121.09, 118.51, 117.99, 110.94, 109.60, 77.88, 77.72, 67.32, 61.89, 61.86, 55.22, 53.88, 53.49, 53.39, 53.23, 40.52, 38.94, 36.50, 31.67, 31.57, 30.97, 30.32, 29.36, 29.35, 29.07, 28.75, 28.56, 27.41, 26.92, 24.70, 23.67, 23.37, 22.61, 22.33, 21.17, 18.58, 15.37, 15.34, 12.97, 10.03, 7.80.

^{31}P NMR (162 MHz, MeOD) δ 32.71.

HRMS (ESI-TOF) m/z: $[M+Na]^+$ calcd. for $C_{42}H_{62}NaN_{11}O_{10}P$, 934.4311; found, 934.433.

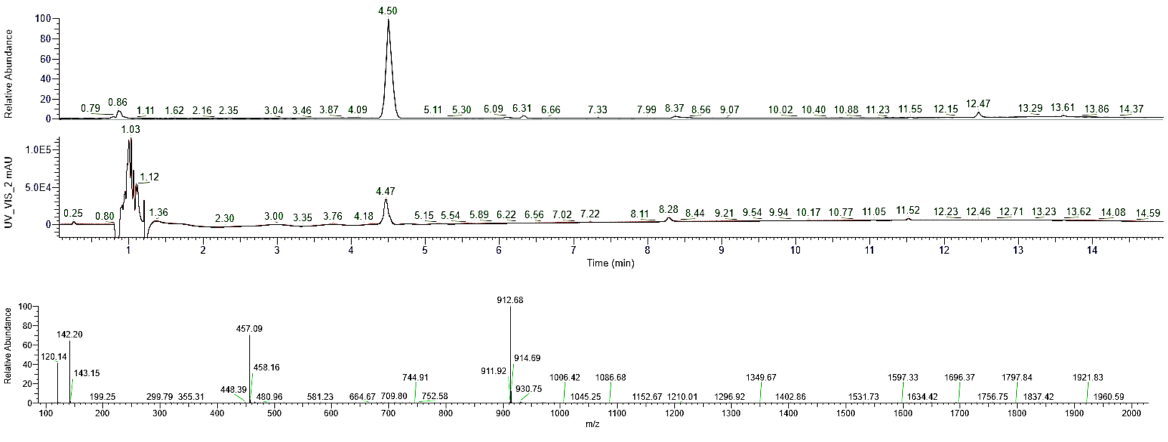

Figure S3. LCMS and ESI-TOF for **37**.

Ac-Arg-(Ala-3-d_1)-Phe-Gln-Trp-NH$_2$ (38)

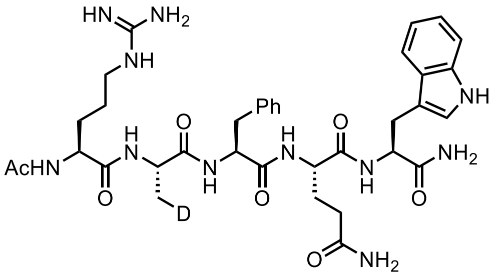

Following general procedure **3.2** and **3.4**, rink amide resin (0.02 mmol) and NHC-BD$_3$ (10.0 equiv) (without radical acceptor) were used to afford the product as a white powder **38** (6.1 mg, 8.2 μmol, 41% yield).

^{1}H NMR (400 MHz, MeOD) δ 7.62 (d, *J* = 7.8 Hz, 1H), 7.30 (d, *J* = 8.1 Hz, 1H), 7.26 – 7.13 (m, 6H), 7.09 – 7.05 (m, 1H), 7.02 (t, *J* = 7.4 Hz, 1H), 4.65 (dd, *J* = 8.3, 5.4 Hz, 1H), 4.42 (dd, *J* = 9.7, 5.0 Hz, 1H), 4.31 – 4.20 (m, 2H), 4.14 (q, *J* = 7.0 Hz, 1H), 3.24 – 3.08 (m, 5H), 3.02 – 2.92 (m, 1H), 2.18 (t, *J* = 7.6 Hz, 2H), 2.02 (s, 3H), 1.95 (tt, *J* = 14.1, 7.2 Hz, 2H), 1.82 – 1.73 (m, 1H), 1.65 (dq, *J* = 20.2, 7.4 Hz, 3H), 1.23 (d, *J* = 7.2 Hz, 2.3H).

^{13}C NMR (101 MHz, MeOD) δ 176.46, 175.26, 174.04, 173.06, 172.45, 171.92, 157.21, 137.11, 136.63, 128.90, 128.10, 127.37, 126.40, 123.24, 121.06, 118.48, 118.00, 110.91, 109.60, 55.28, 53.90, 53.52, 53.34, 40.51, 36.28, 30.98, 28.48, 27.40, 26.89, 24.68, 21.17, 15.94, 7.79.

HRMS (ESI-TOF) m/z: [M+K]$^+$ calcd. for $C_{36}H_{48}DKN_{11}O_7$, 787.3511; found, 787.3518.

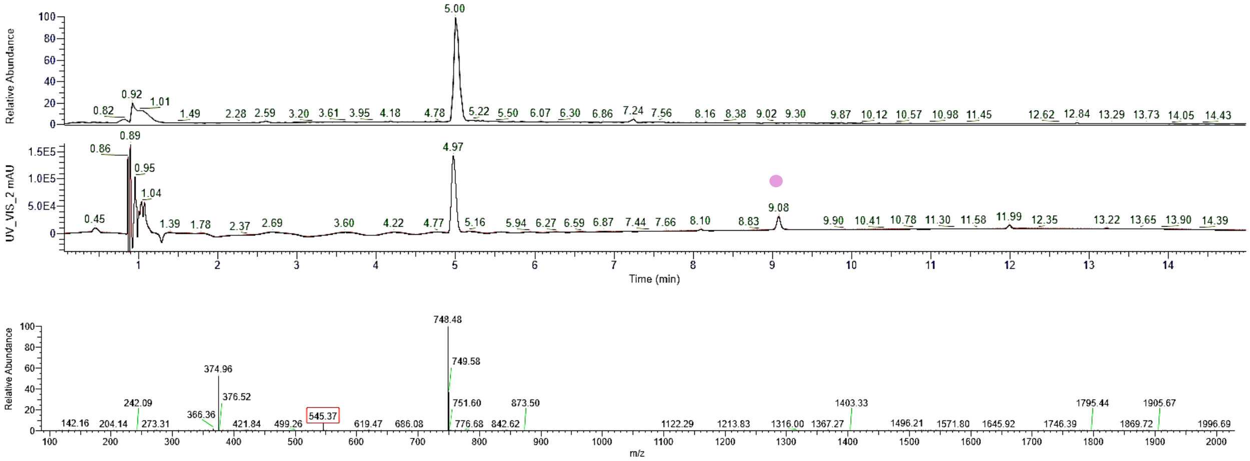

Figure S4. LCMS and ESI-TOF for **38**. ● Small molecular impurity (142) from LCMS column

Ac-Arg-(Homo-Gln)-Phe-Gln-Trp-NH2 (40)

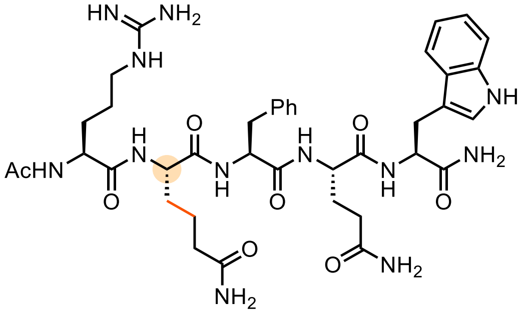

Following general procedure **3.2** and **3.4**, rink amide resin (0.02 mmol) and acrylamide (8.0 equiv) were used to afford the product as a white powder **40** (3.7 mg, 4.6 µmol, 23% yield).

^{1}H NMR (400 MHz, MeOD) δ 7.64 (d, *J* = 7.8 Hz, 1H), 7.31 (d, *J* = 8.0 Hz, 1H), 7.28 – 7.17 (m, 5H), 7.15 (s, 1H), 7.06 (dt, *J* = 22.9, 7.3 Hz, 2H), 4.65 (dd, *J* = 8.3, 5.5 Hz, 1H), 4.48 (dd, *J* = 9.8, 5.0 Hz, 1H), 4.27 (td, *J* = 13.7, 13.2, 5.9 Hz, 2H), 4.15 (dd, *J* = 8.6, 4.8 Hz, 1H), 3.17 (tdd, *J* = 14.2, 10.1, 4.8 Hz, 5H), 2.95 (dd, *J* = 14.2, 9.7 Hz, 1H), 2.18 (dt, *J* = 14.4, 7.1 Hz, 3H), 2.03 (s, 2H), 1.96 (dt, *J* = 14.7, 6.9 Hz, 1H), 1.78 (q, *J* = 6.8, 6.4 Hz, 1H), 1.66 (m, 3H).

^{13}C NMR (101 MHz, MeOD) δ 178.40, 177.83, 176.63, 174.63, 174.61, 174.35, 173.80, 173.26, 158.62, 138.47, 138.04, 130.29, 129.53, 127.83, 124.62, 122.48, 119.90, 119.41, 112.33, 111.01, 56.61, 55.36, 55.28, 54.88, 41.93, 37.85, 35.43, 32.40, 31.57, 29.80, 28.82, 28.33, 26.12, 22.70, 22.58.

HRMS (ESI-TOF) m/z: $[M]^+$ calcd. for $C_{39}H_{54}N_{12}O_8$, 818.4188; found, 818.4207.

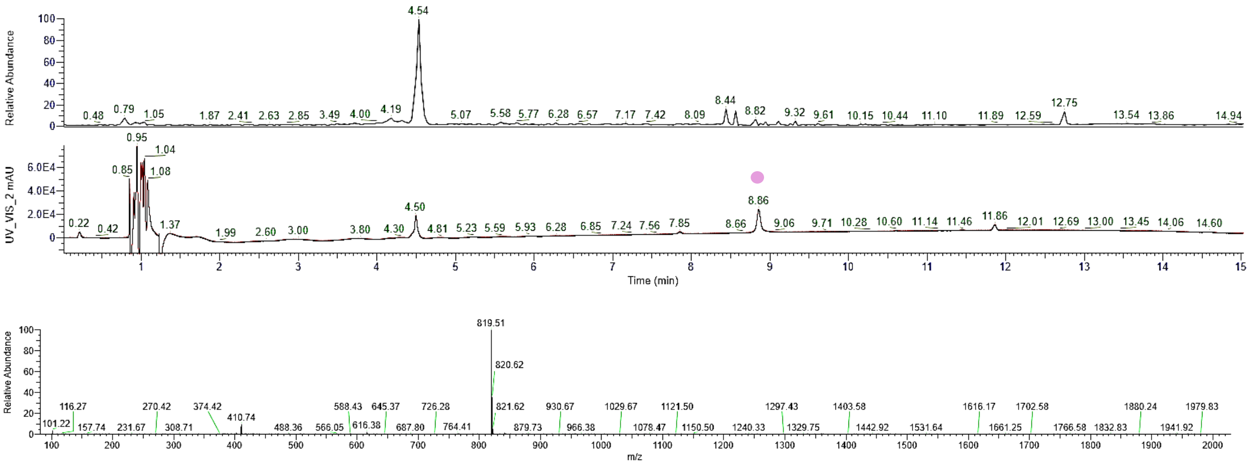

Figure S5. LCMS and ESI-TOF for **40**. ● Small molecular impurity (142) from LCMS column

Boc-Tyr-Ala($CH_2CH_2PO(OEt)_2$)-Trp-OMe (41)

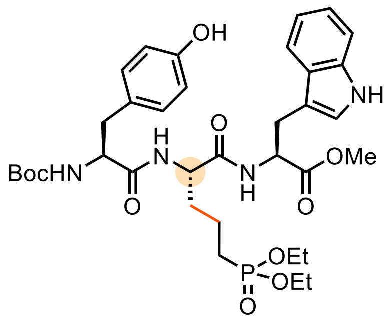

Following general procedure **3.3**, Boc-Tyr-Ser-Trp-OMe (10.0 mg, 18.0 μmol) and diethyl vinylphosphonate (8.0 equiv) were used to afford the product as a white powder **41** (5.5 mg, 7.7 μmol, 44% yield).

^{1}H NMR (400 MHz, MeOD) δ 7.50 (d, J = 7.9 Hz, 1H), 7.31 (d, J = 8.1 Hz, 1H), 7.15 – 7.05 (m, 2H), 7.03 – 6.98 (m, 3H), 6.68 (d, J = 8.3 Hz, 2H), 4.70 (dd, J = 7.4, 5.8 Hz, 1H), 4.59 – 4.52 (m, 1H), 4.39 (dd, J = 8.3, 5.6 Hz, 1H), 4.23 (dd, J = 8.9, 5.4 Hz, 1H), 4.12 – 4.02 (m, 4H), 3.64 (s, 3H), 3.29 – 3.27 (m, 1H), 3.25 – 3.16 (m, 1H), 2.96 (dd, J = 14.1, 5.2 Hz, 1H), 2.69 (dd, J = 14.1, 9.1 Hz, 1H), 1.88 – 1.75 (m, 3H), 1.71 – 1.56 (m, 3H), 1.36 (s, 9H), 1.31 (t, J = 7.1, 1.2 Hz, 6H).

^{13}C NMR (101 MHz, MeOD) δ 174.41, 173.64, 173.44, 157.64, 157.22, 137.99, 131.36, 130.57, 129.19, 128.71, 124.64, 122.43, 119.87, 119.14, 116.19, 112.34, 110.42, 80.69, 63.24, 63.21, 57.57, 54.83, 53.66, 52.70, 40.35, 38.41, 31.72, 30.15, 28.67, 19.66, 16.77, 16.74, 11.43.

^{31}P NMR (162 MHz, MeOD) δ 32.84.

HRMS (ESI-TOF) m/z: $[M+Na]^+$ calcd. for $C_{35}H_{49}N_4NaO_{10}P$, 739.3079; found, 739.3071.

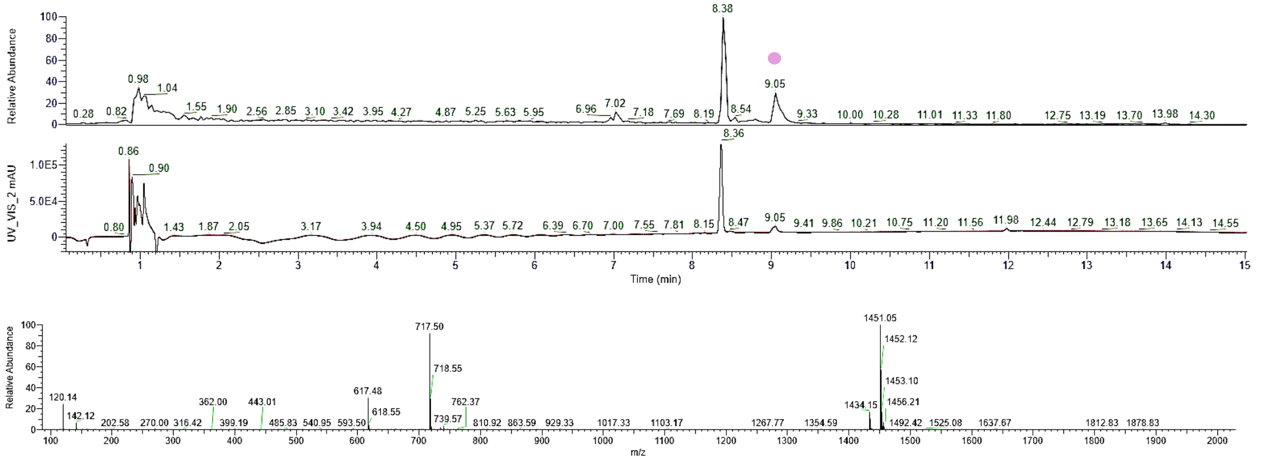

Figure S6. LCMS and ESI-TOF for **41**. ● Small molecular impurity (270) from LCMS column

Ac-Tyr-Gly-Gly-Phe-Ala($CH_2CH_2PO(OEt)_2$)-NH_2 (42)

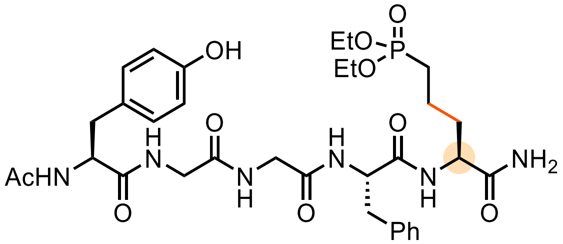

Following general procedure **3.2** and **3.4**, rink amide resin (0.02 mmol) and diethyl vinylphosphonate (8.0 equiv) were used to afford the product as a white powder **42** (5.1 mg, 7.2 μmol, 36% yield).

^{1}H NMR (400 MHz, MeOD) δ 7.29 (d, J = 4.4 Hz, 4H), 7.25 – 7.18 (m, 1H), 7.06 (d, J = 8.1 Hz, 2H), 6.71 (d, J = 8.0 Hz, 2H), 4.60 (dd, J = 9.2, 5.4 Hz, 1H), 4.48 (t, J = 7.4 Hz, 1H), 4.31 (dd, J = 9.1, 4.8 Hz, 1H), 4.09 (ddh, J = 10.4, 7.3, 3.1 Hz, 4H), 3.87 – 3.64 (m, 4H), 3.47 (p, J = 6.6 Hz, 2H), 3.24 – 3.17 (m, 2H), 3.04 (dt, J = 14.1, 8.7 Hz, 2H), 2.86 (dd, J = 13.8, 8.4 Hz, 1H), 1.95 (s, 3H), 1.88 – 1.37 (m, 6H), 1.32 (t, J = 6.6 Hz, 6H).

^{13}C NMR (126 MHz, MeOD) δ 174.85, 173.53, 171.83, 157.41, 138.46, 131.26, 130.32, 129.59, 128.93, 127.89, 116.27, 63.26, 63.21, 57.09, 56.63, 54.05, 43.98, 43.67, 38.31, 37.83, 25.86, 24.74, 22.53, 20.07, 19.32, 16.78, 16.73, 9.20.

^{31}P NMR (202 MHz, MeOD) δ 32.76.

HRMS (ESI-TOF) m/z: $[M+H]^+$ calcd. for $C_{33}H_{48}N_6O_{10}P$, 719.3164; found, 719.3174.

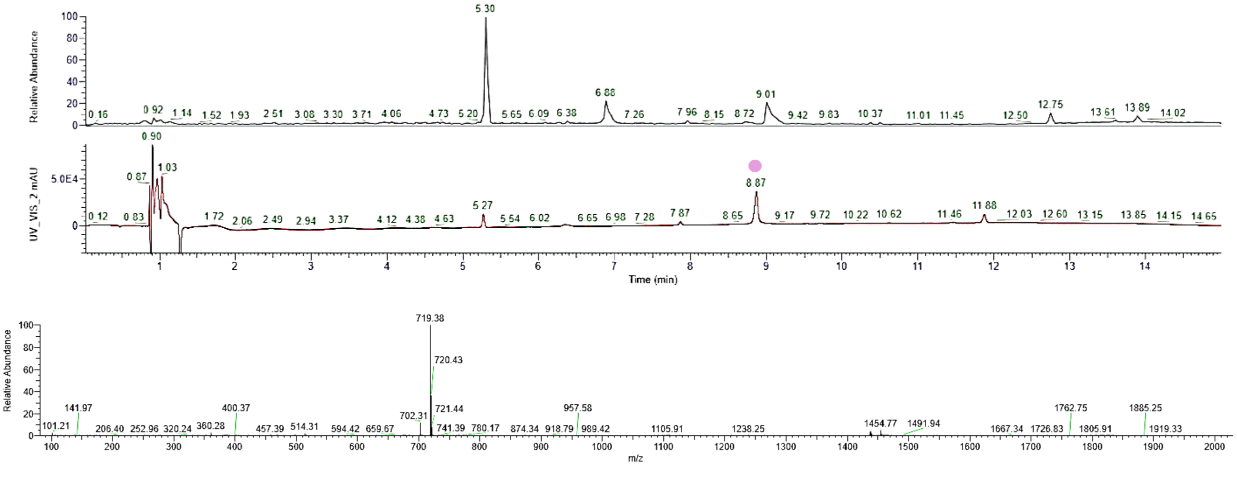

Figure S7. LCMS and ESI-TOF for **42**. ● Small molecular impurity (142) from LCMS column

Bradykinin Ac-Arg-Pro-Pro-Gly-Phe-Ala($CH_2CH_2PO(OEt)_2$)-Pro-Phe-Arg-NH_2 (43)

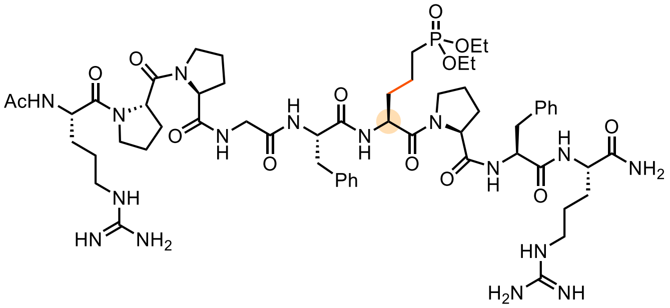

Following general procedure **3.2** and **3.4**, rink amide resin (0.02 mmol) and diethyl vinylphosphonate (8.0 equiv) were used to afford the product as a white powder **43** (4.4 mg, 3.6 μmol, 18% yield).

^{1}H NMR (800 MHz, MeOD) δ 7.32 – 7.25 (m, 6H), 7.24 – 7.17 (m, 4H), 4.68 (dd, *J* = 8.4, 5.3 Hz, 1H), 4.64 – 4.60 (m, 4H), 4.52 (m, 1H), 4.37 (dd, *J* = 8.3, 5.5 Hz, 1H), 4.30 (td, *J* = 6.5, 5.7, 3.1 Hz, 3H), 4.10 (m, 4H), 3.94 – 3.91 (m, 1H), 3.88 (dt, *J* = 10.6, 5.9 Hz, 1H), 3.84 (dt, *J* = 9.5, 7.0 Hz, 1H), 3.76 (d, *J* = 16.9 Hz, 1H), 3.67 (dt, *J* = 9.8, 6.7 Hz, 1H), 3.60 – 3.48 (m, 3H), 3.20 (t, *J* = 7.0 Hz, 1H), 3.19 – 3.14 (m, 4H), 3.10 (dd, *J* = 13.9, 8.6 Hz, 1H), 3.00 – 2.95 (m, 1H), 2.30 (ddd, *J* = 11.5, 8.3, 5.3 Hz, 1H), 2.22 (dq, *J* = 12.5, 7.4 Hz, 1H), 2.13 – 2.06 (m, 2H), 2.04 – 1.99 (m, 2H), 1.99 – 1.94 (m, 5H), 1.89 – 1.84 (m, 4H), 1.81 (m, 2H), 1.75 (dt, *J* = 11.9, 5.9 Hz, 2H), 1.68 (m, 4H), 1.61 (m, 3H), 1.51 – 1.42 (m, 3H), 1.38 – 1.35 (m, 3H). 1.33 (t, J = 7.1, Hz, 6H).

^{13}C NMR (201 MHz, MeOD) δ 175.03, 173.54, 173.17, 172.83, 172.06, 171.39, 170.32, 169.35, 167.15, 158.62, 138.41, 135.58, 130.57, 130.38, 130.27, 129.64, 129.58, 127.98, 127.94, 69.11, 68.71, 63.33, 62.06, 61.96, 59.89, 56.67, 56.25, 56.05, 53.81, 52.54, 51.99, 49.39, 43.45, 42.13, 41.92, 40.35, 40.19, 39.15, 38.51, 37.90, 33.08, 31.72, 31.63, 30.75, 30.53, 30.48, 30.34, 30.15, 30.07, 29.56, 29.39, 25.70, 25.07, 24.96, 24.04, 24.02, 23.75, 22.24, 14.44, 14.37, 11.43, 11.41.

^{31}P NMR (202 MHz, MeOD) δ 32.78.

HRMS (ESI-TOF) m/z: $[M+Na]^+$ calcd. for $C_{58}H_{89}NaN_{16}O_{13}P$, 1271.6425; found, 1271.6462.

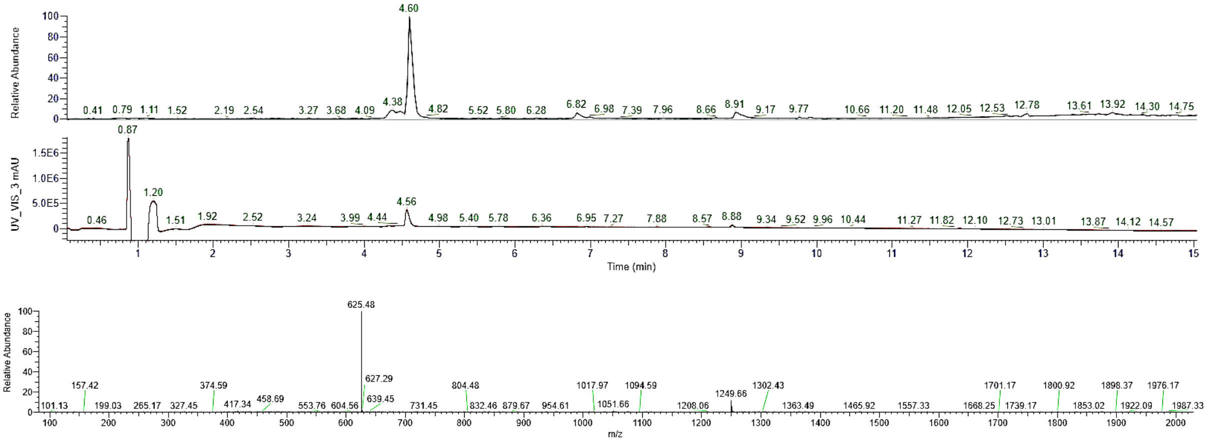

Figure S8. LCMS and ESI-TOF for **43**.

α-MSH Ac-Ala($CH_2CH_2PO(OEt)_2$)-Tyr-Ser-Met-Glu-His-Phe-Arg-Trp-Gly-Lys-Pro-Val-NH_2 (44)

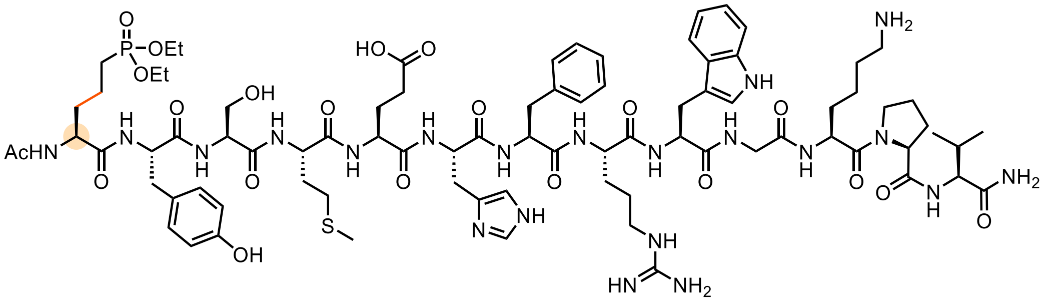

Following general procedure **3.2** and **3.4**, rink amide resin (0.01 mmol) and diethyl vinylphosphonate (8.0 equiv) were used to afford the product as a white powder **44** (3.6 mg, 2.0 μmol, 20% yield).

^{1}H NMR (800 MHz, MeOD) δ 7.85 – 7.80 (m, 2H), 7.59 (dd, J = 11.0, 7.9 Hz, 2H), 7.55 (d, J = 7.6 Hz, 1H), 7.35 (d, J = 8.3 Hz, 1H), 7.31 – 7.28 (m, 3H), 7.22 (q, J = 7.3, 5.8 Hz, 1H), 7.16 (d, J = 8.8 Hz, 2H), 7.10 (q, J = 8.0 Hz, 2H), 6.72 (d, J = 8.0 Hz, 1H), 4.73 (dd, J = 8.7, 5.4 Hz, 1H), 4.69 (dd, J = 8.7, 5.2 Hz, 1H), 4.66 – 4.63 (m, 1H), 4.56 (dd, J = 9.8, 4.8 Hz, 1H), 4.50 (dt, J = 8.5, 4.3 Hz, 4H), 4.40 (q, J = 5.1, 4.5 Hz, 1H), 4.36 (t, J = 6.2 Hz, 1H), 4.31 (dd, J = 5.7, 4.0 Hz, 2H), 4.23 (d, J = 8.9 Hz, 2H), 4.18 (d, J = 3.8 Hz, 1H), 4.16 (t, J = 6.3 Hz, 2H), 4.10 (qd, J = 7.2, 4.0 Hz, 3H), 4.06 (t, J = 6.8 Hz, 1H), 4.03 – 4.00 (m, 1H), 3.95 (d, J = 6.1 Hz, 1H), 3.84 – 3.80 (m, 4H), 3.70 – 3.65 (m, 6H), 3.15 – 3.05 (m, 7H), 2.95 (dd, J = 17.7, 7.1 Hz, 5H), 2.68 – 2.67 (m, 1H), 2.61 (d, J = 6.9 Hz, 1H), 2.59 – 2.55 (m, 1H), 2.50 – 2.47 (m, 1H), 2.39 (s, 1H), 2.23 – 2.15 (m, 4H), 2.08 (s, 3H), 2.06 (s, 3H), 1.99 (dd, J = 9.4, 4.5 Hz, 3H), 1.87 (d, J = 7.0 Hz, 3H), 1.77 (dt, J = 12.3, 6.1 Hz, 4H), 1.65 – 1.61 (m, 2H), 1.51 (dt, J = 13.8, 6.9 Hz, 4H), 1.46 (dp, J = 10.7, 4.4, 3.7 Hz, 2H), 1.41 – 1.37 (m, 6H), 1.33 (t, J = 7.4 Hz, 6H), δ 0.98 – 0.95 (m, 6H).

^{31}P NMR (202 MHz, MeOD) δ 32.65.

HRMS (ESI-TOF) m/z: $[M]^+$ calcd. for $C_{83}H_{122}N_{21}O_{21}PS$, 1811.8582; found, 1811.8512.

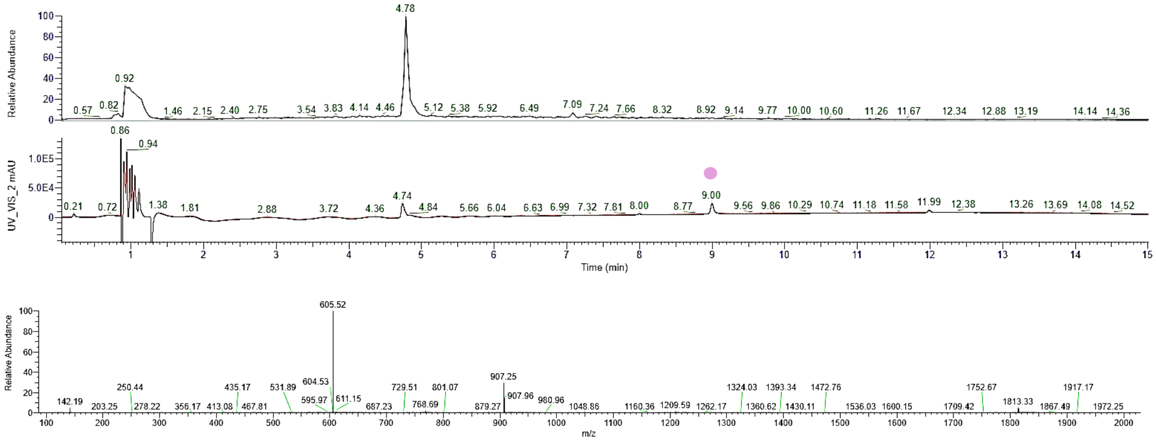

Figure S9. LCMS and ESI-TOF for **44**. ● Small molecular impurity (142) from LCMS column

Atosiban-3 (45)[10]

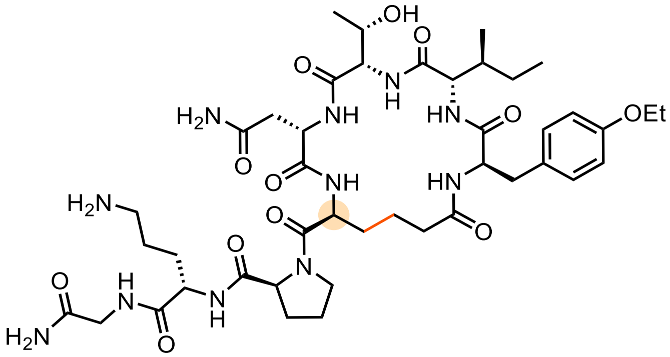

Following general procedure **3.2** and **3.4**, rink amide resin (0.02 mmol) were used to afford the product as a white powder **45** (6.5 mg, 7.0 µmol, 35% yield).

^{1}H NMR (400 MHz, MeOD) δ 7.16 (d, *J* = 8.3 Hz, 2H), 6.85 (d, *J* = 8.4 Hz, 2H), 5.14 (s, 1H), 4.64 (t, *J* = 6.5 Hz, 1H), 4.60 (q, *J* = 4.8, 3.4 Hz, 1H), 4.54 (dd, *J* = 10.5, 5.8 Hz, 1H), 4.44 (d, *J* = 3.6 Hz, 1H), 4.39 (t, *J* = 7.0 Hz, 1H), 4.30 (d, *J* = 5.6 Hz, 1H), 4.14 (d, *J* = 3.9 Hz, 1H), 4.00 (q, *J* = 7.0 Hz, 2H), 3.90 (d, *J* = 2.7 Hz, 1H), 3.80 (d, *J* = 10.0 Hz, 1H), 3.63 (dd, *J* = 17.4, 8.7 Hz, 1H), 3.22 (q, *J* = 7.3 Hz, 1H), 3.05 – 2.98 (m, 2H), 2.97 – 2.90 (m, 1H), 2.87 (dd, *J* = 15.5, 6.3 Hz, 1H), 2.70 (dd, *J* = 15.8, 6.4 Hz, 1H), 2.46 – 2.29 (m, 2H), 2.19 – 2.09 (m, 1H), 2.07 – 1.74 (m, 10H), 1.71 – 1.57 (m, 1H), 1.55 – 1.43 (m, 5H), 1.37 (t, *J* = 6.9 Hz, 3H), 1.33 (d, *J* = 7.2 Hz, 2H), 1.27 (d, *J* = 6.4 Hz, 3H), 1.01 – 0.95 (m, 3H), 0.82 (t, *J* = 7.3 Hz, 3H).

^{13}C NMR (101 MHz, MeOD) δ 178.00, 175.67, 174.94, 174.92, 174.31, 173.87, 171.89, 171.42, 167.15, 159.62, 131.43, 130.57, 115.77, 68.71, 68.50, 64.49, 62.35, 60.36, 60.13, 57.92, 52.71, 52.37, 52.25, 42.89, 40.35, 40.25, 36.94, 36.78, 36.18, 35.57, 33.08, 31.72, 31.05, 30.75, 30.47, 30.15, 29.55, 26.51, 25.08, 24.95, 24.02, 23.95, 23.74, 23.02, 20.15, 16.14, 15.17, 14.36, 12.44, 11.42, 9.20.

HRMS (ESI-TOF) m/z: $[M+H]^+$ calcd. for $C_{43}H_{68}N_{11}O_{12}$, 930.5043; found, 930.5070.

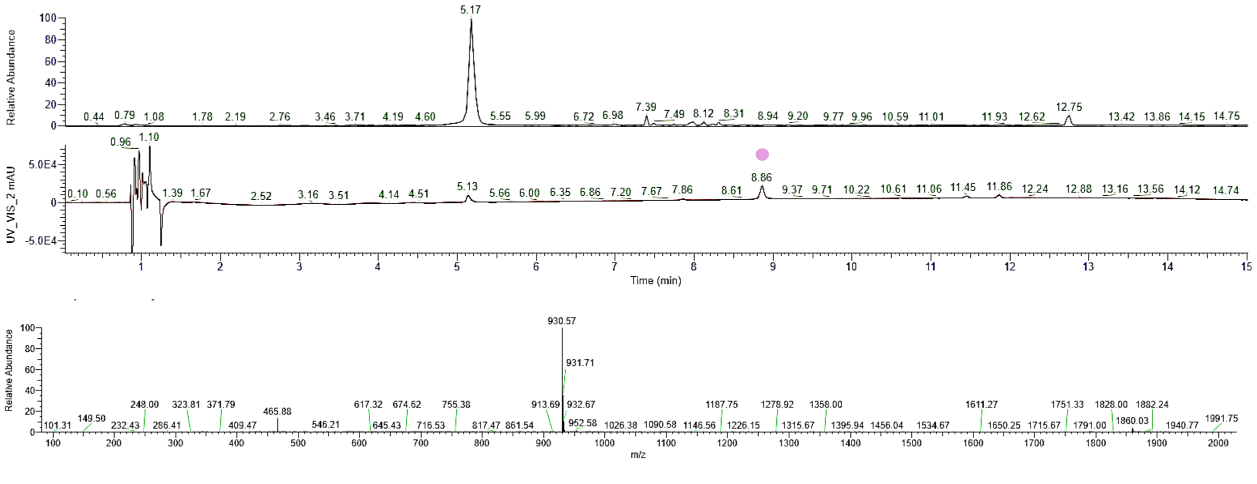

Figure S10. LCMS and ESI-TOF for **45**. ● Small molecular impurity (142) from LCMS column

6. Stern-Volmer Quenching Studies

Fluorescence quenching experiments were performed on a Horiba PTI QM400 spectrofluorometer using a 10.0 mm quartz cuvette. The photocatalyst **6** was prepared as a 0.01 mM solution in degassed DMSO. The solution was irradiated at λ_{ex} = 360 nm, and the emission was observed at λ_{em} = 437 nm. The excited state lifetime τ_0 in DMSO is reported in the literature.[11]

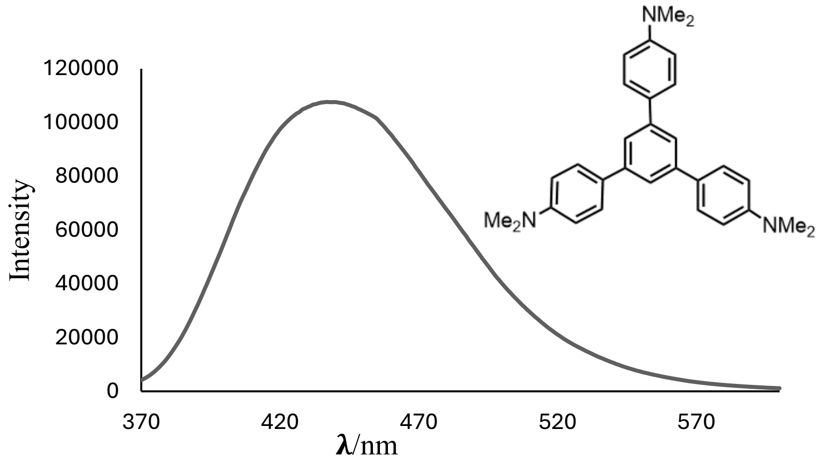

Fig S11. Fluorescence emission spectrum of PC **6** (excited at 360 nm). The $\lambda_{max,em}$ was 437 nm.

The quenching experiments were conducted using a 0.01 mM solution of PC **6** with varying concentrations of Phosphite-Ser **3**, NHC-BH_3, acrylonitrile in DMSO, and HCO_2K in a DMSO:H_2O (3:1) mixture. The samples were prepared in 4 mL quartz cuvettes sealed with PTFE stoppers. Following one minute of nitrogen degassing, the emission intensity was measured at room temperature.

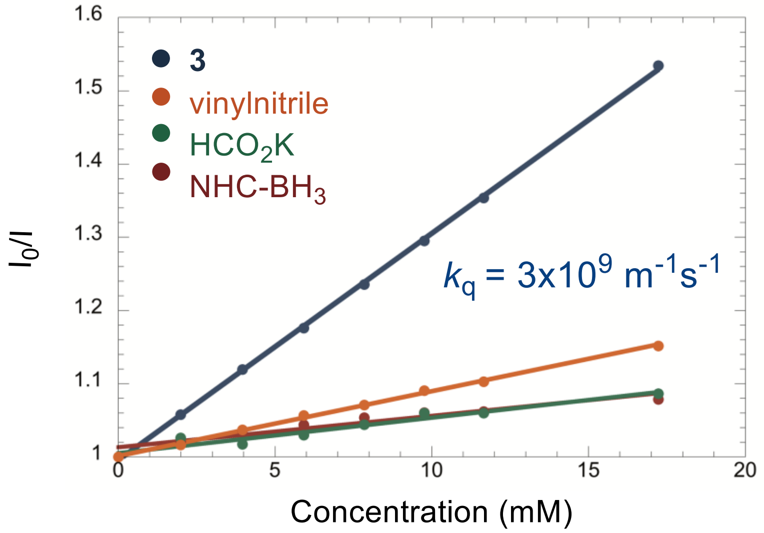

Figure S12. Stern-Volmer quenching studies for Phosphite-Ser **3**, NHC-BH_3, acrylonitrile and HCO_2K.

7. Cyclic Voltammetry Measurement

The electrochemical experiment was performed using a standard three-electrode setup inside a nitrogen-filled glovebox. A 3 mm diameter glassy carbon electrode (0.071 cm^2, MF-2012 from BASi) was used as the working electrode, with a platinum wire (CHI115 from CH Instruments, Inc.) as the counter electrode, and a 0.5 mm diameter silver wire (MF-2017 from BASi) as the reference electrode. Ferrocene/ferrocenium (Fc/Fc^+) was added as an internal standard for potential calibration. Tetrabutylammonium hexafluorophosphate ($n\text{-}Bu_4NPF_6$) was dried under high vacuum at 90 °C overnight prior to use. Cyclic voltammograms (CVs) were recorded following IUPAC conventions, where negative currents indicate reduction processes. Due to irreversibility for the first reduction event, the half-peak potential (Ep/2) was used as an approximation of E1/2. This value was used for later comparisons of redox potentials.

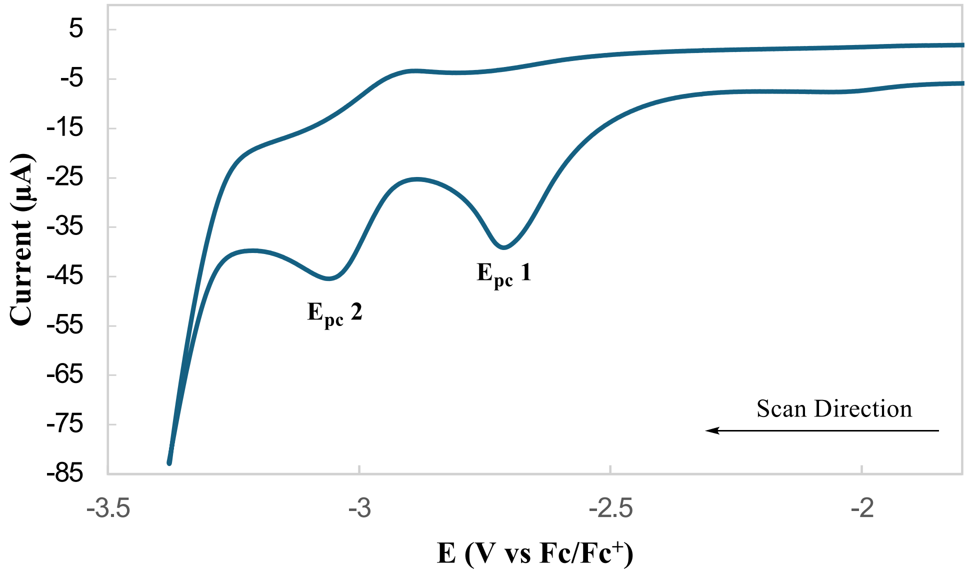

Figure S13. Cyclic Voltammogram of Phosphite-Ser **3**. Ep/2 1 = -2.57 V (vs Fc/Fc+); Epc 1 = -2.71 V (vs Fc/Fc+); Epc 2 = -3.06 V (vs Fc/Fc+). solvent = DMSO; temperature = 295 K; scan rate = 500 mv/s; [Ser-Phosphite] = 1.0 mM; [$TBAPF_6$] = 100 mM.

8. Radical Trapping Experiments with TEMPO

Reactions were performed following the general procedure with Phosphite-Ser **3** (21.1 mg, 0.04 mmol), PC (0.5 mg, 3 mol%), NHC-BH_3 (22.4 mg, 5.0 equiv), HCO_2K (6.7 mg, 2.0 equiv), and TEMPO (6.2 mg, 1.0 equiv) in DMSO/H_2O = 20:1 (1.5 mL). The crude residue was analyzed by HRMS.

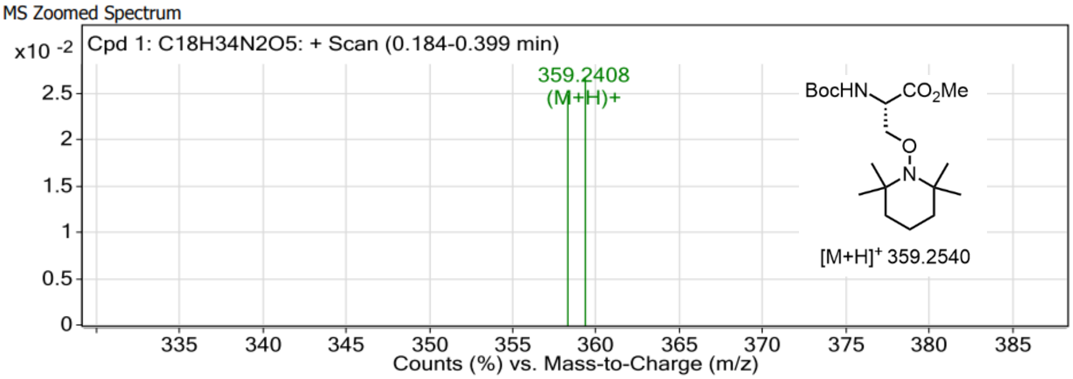

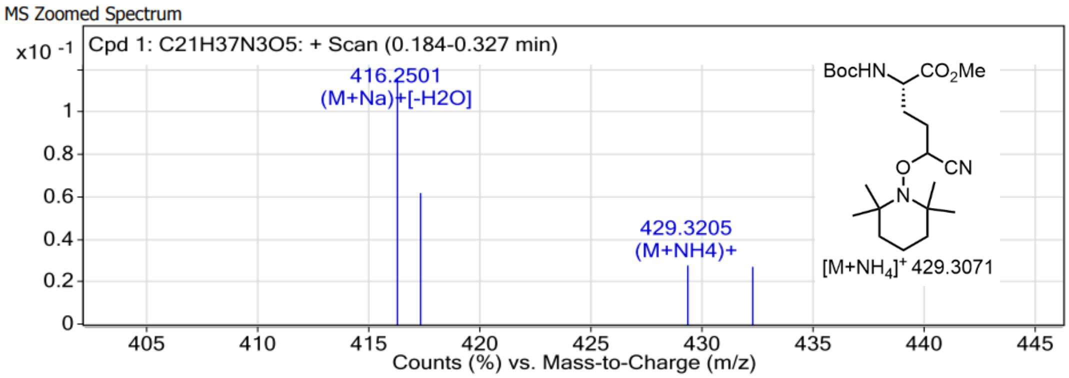

Figure S14. Radical Trapping Experiment with 1.0 equiv TEMPO.

Reactions were performed following the general procedure with Phosphite-Ser **3** (21.1 mg, 0.04 mmol), PC (0.5 mg, 3 mol%), NHC-BH_3 (22.4 mg, 5.0 equiv), HCO_2K (6.7 mg, 2.0 equiv), and TEMPO (6.2 mg, 3.0 equiv) in DMSO/H_2O = 20:1 (1.5 mL). The crude residue was analyst by HRMS.

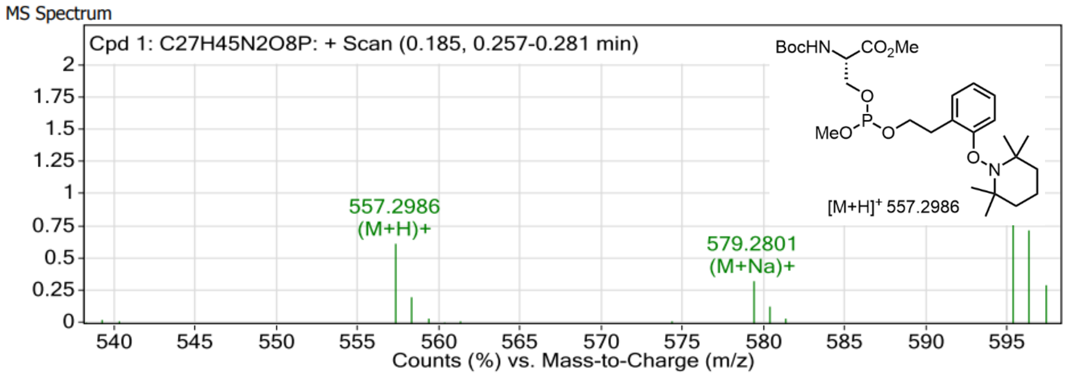

Figure S15. Radical Trapping Experiment with 3.0 equiv TEMPO.

9. Deuterium labeling experiments

Synthesis of NHC-BD_3

NHC-BD_3 was prepared following the same procedures as for NHC-BH_3, substituting $NaBD_4$ (1.2 equiv) for $NaBH_4$. Purification was performed by flash chromatography, ^1H NMR spectrum indicated 70% deuterium incorporation.

^{1}H NMR (400 MHz, $CDCl_3$) δ 6.79 (s, 1H), 3.74 (s, 3H), 1.41 – 0.55 (m, 1H).

Synthesis of DCO_2K [12]

DCO_2D (0.25 mL, 6.5 mmol) was added K_2CO_3 (690 mg, 5.0 mmol), and the mixture was stirred at 100 °C under an argon atmosphere for 3 h. The mixture was concentrated with vacuum to afford DCO_2K as a white solid (400 mg, 94% yield).

Deuterium labeling experiments were performed following general procedures. The product from each experiment was purified by flash chromatography to NMR analysis. Approximately 70% deuterium incorporation was observed in reactions employing NHC–BD_3, while no deuterium incorporation was detected in experiments using DCO_2K or D_2O.

Methyl (2S)-2-((tert-butoxycarbonyl)amino)-5-cyanopentanoate-5-d (S3)

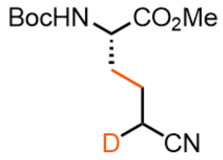

^{1}H NMR (600 MHz, $CDCl_3$) δ 5.08 (s, 1H), 4.34 (s, 1H), 3.92 – 3.58 (m, 3H), 2.43 – 2.35 (m, **1.3H**), 2.05 – 1.78 (m, 1H), 1.76 (dd, J = 12.9, 7.0 Hz, 3H), 1.45 (t, J = 3.8 Hz, 9H).

^{13}C NMR (151 MHz, $CDCl_3$) δ 172.57, 155.38, 119.06, 80.27, 52.57, 52.53, 31.98, 28.29, 21.60, 21.51, 16.79, 16.70, 16.57, 16.43.

HRMS (ESI-TOF) m/z: $[M+Na]^+$ calcd. for $C_{12}H_{19}DNaN_2O_4$, 280.1378; found, 280.1374.

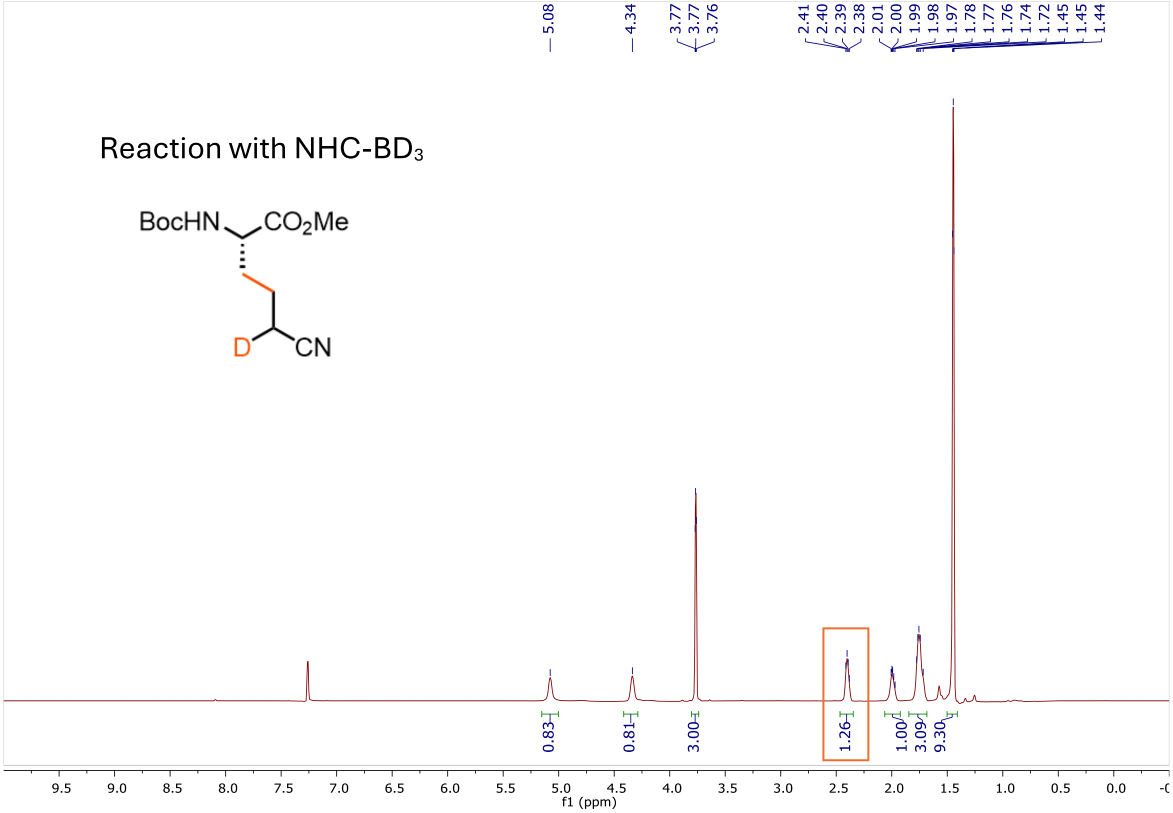

Figure S16. ^{1}H NMR (600 MHz, $CDCl_3$) of **S3**

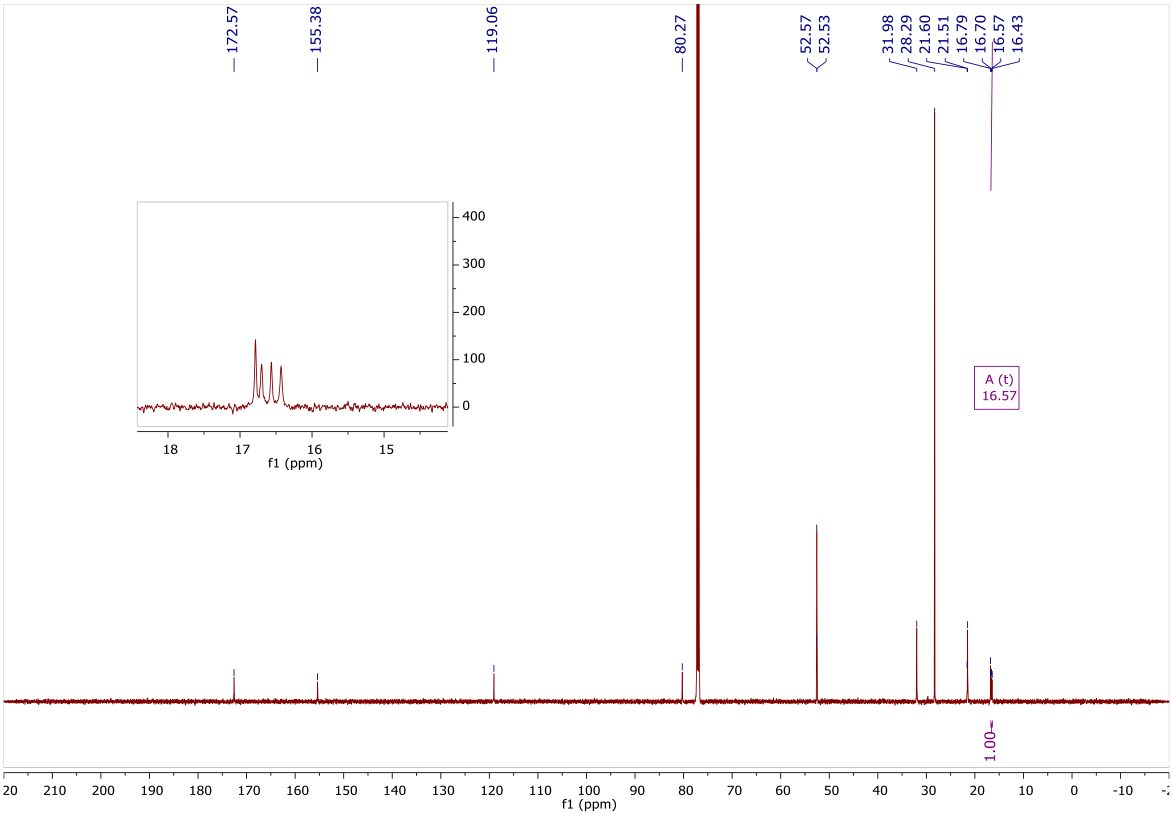

Figure S17. ^{13}C NMR (151 MHz, $CDCl_3$) of **S3**

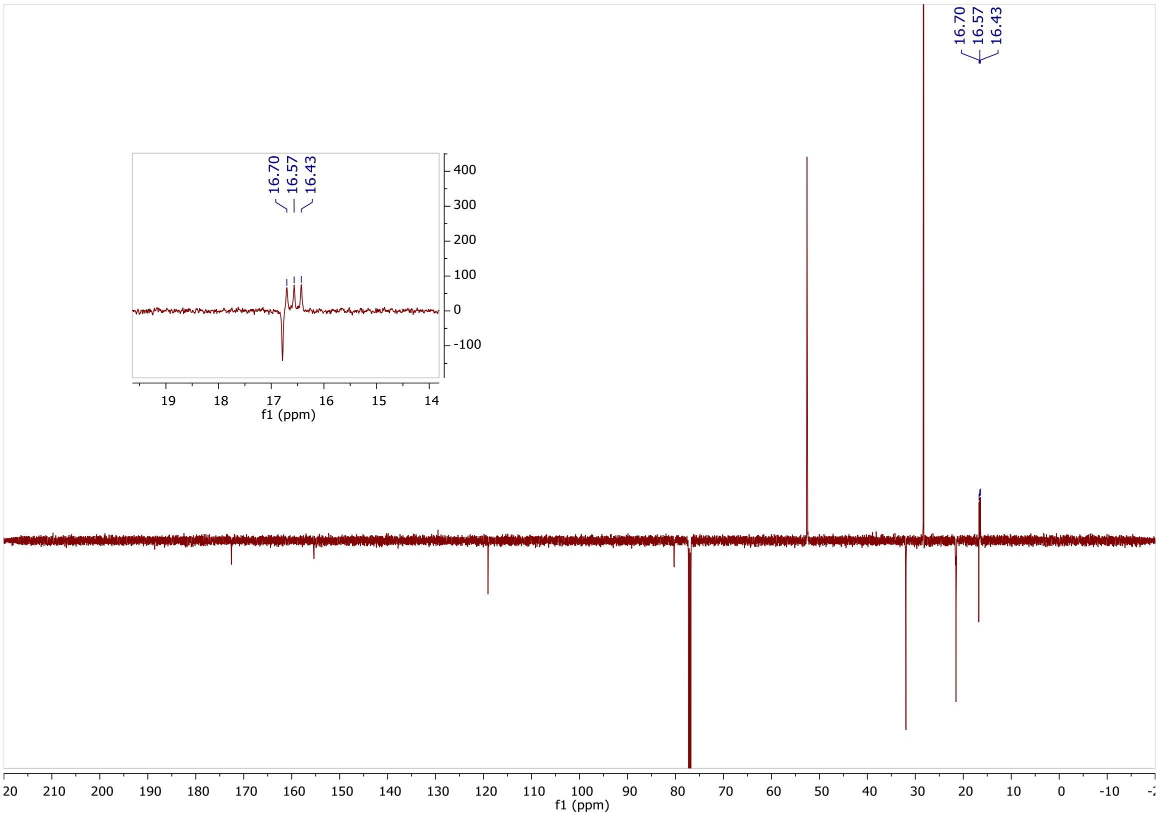

Figure S18. **^{13}C APT NMR** (101 MHz, $CDCl_3$) of **S3**

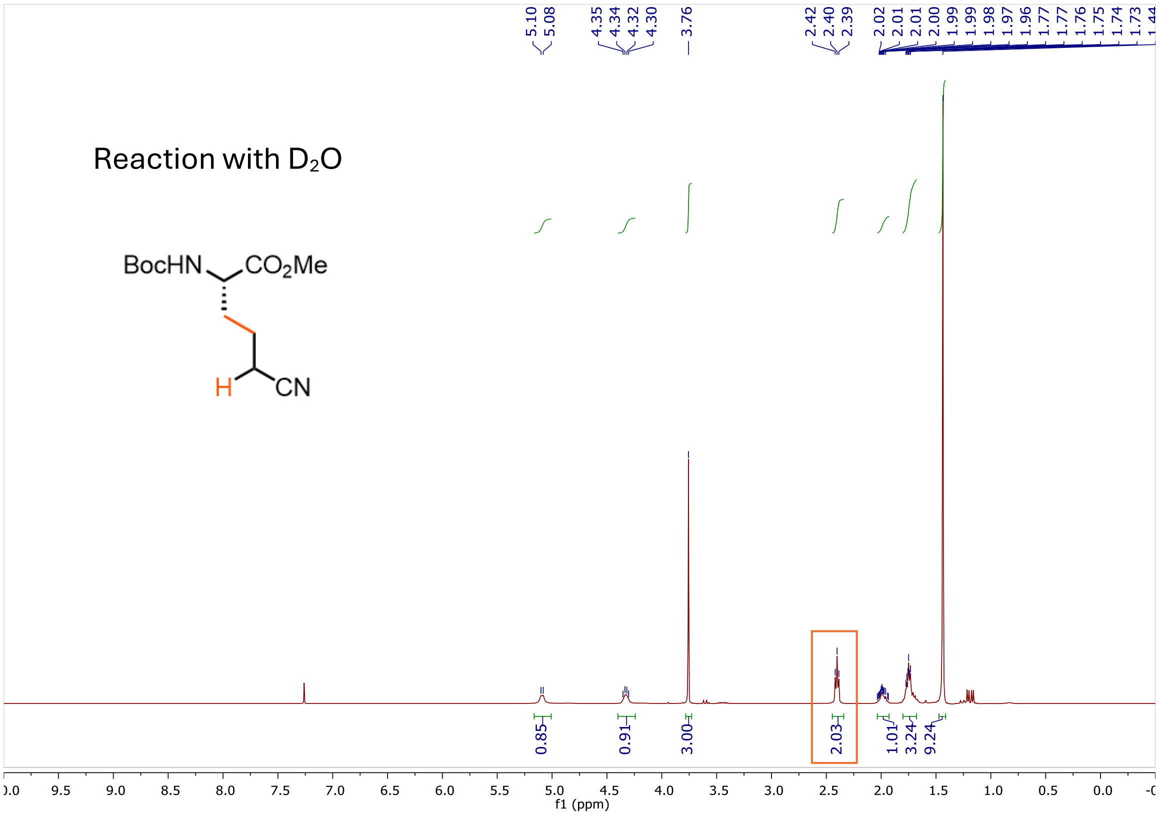

Figure S19. **^{1}H NMR** (400 MHz, $CDCl_3$) of **4**

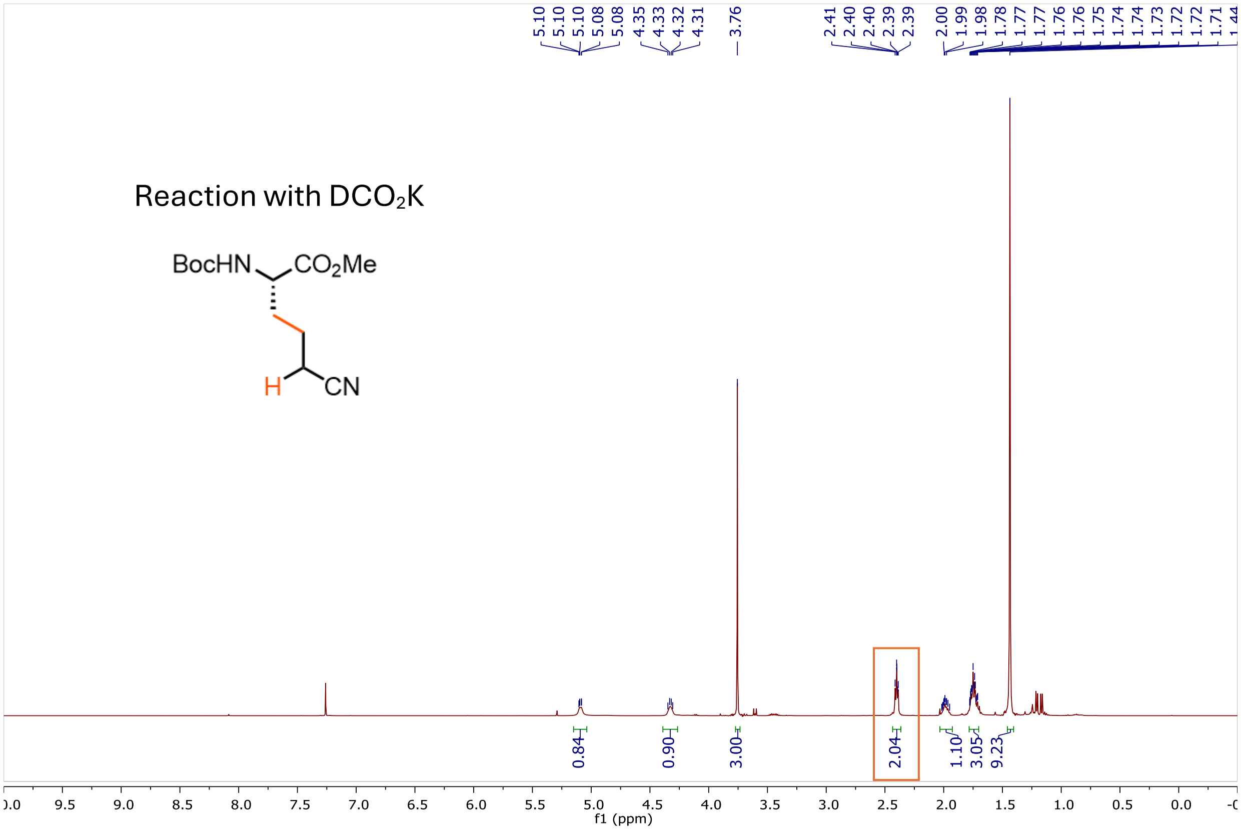

Figure S20. ^{1}H NMR (400 MHz, $CDCl_3$) of **4**

10. Light On-Off Experiments

Reactions were performed following the general procedure with Phosphite-Ser (21.1 mg, 0.04 mmol), PC (0.5 mg, 3 mol%), NHC-BH_3 (22.4 mg, 5.0 equiv), HCO_2K (6.7 mg, 2.0 equiv), and mesitylene (5.6 µl, 1.0 equiv) as internal standard and DMSO/H_2O = 20:1 (1.5 mL). The crude residue was analyst by GC-FID.

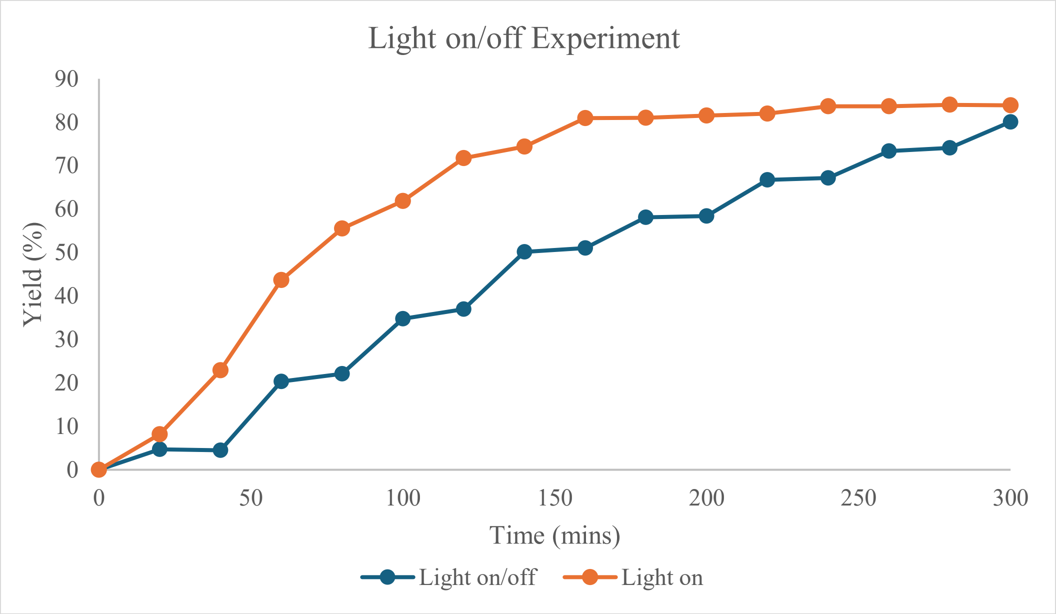

Figure S21. Light on-off experiments

11. Confirmation of the Retention Stereochemistry of 18

The retention of chirality of **18** was obtained by the general procedure **3.1**, and race mixture was obtained by the following methods.

6-benzyl 1-methyl 2-((tert-butoxycarbonyl)amino)hexanedioate (S4)[13]

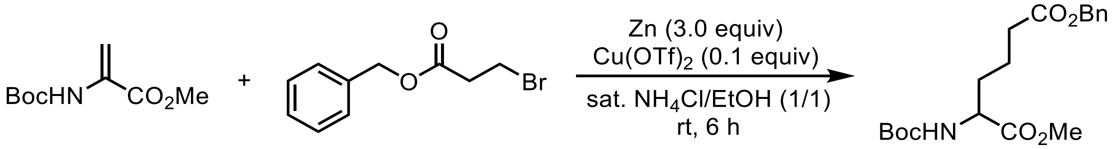

To a stirred solution of Dha (1.7 mmol) in a 1:1 (v/v) mixture of saturated NH_4Cl and EtOH (15 mL) were added $Cu(OTf)_2$ (0.1 equiv), Zn (3.0 equiv) and alkyl bromide (3.0 equiv) at room temperature. After 3 h, additional Zn (1.0 equiv) and alkyl bromide (1.0 equiv) were added. After a further 2 h, another portion of Zn (1.0 equiv) and alkyl bromide (1.0 equiv) was added. The reaction mixture was stirred for an additional 1 h. The resulting mixture was extracted with ethyl acetate. The combined organic layers were dried over Na_2SO_4, filtered, and concentrated with vacuum. The residue was purified by column chromatography (1: 9 to 1: 4 ethyl acetate: hexanes) to give product **S4** with 51% yield.

^{1}H NMR (400 MHz, $CDCl_3$) δ 7.42 – 7.29 (m, 5H), 5.11 (s, 2H), 5.02 (d, *J* = 8.5 Hz, 1H), 4.42 – 4.21 (m, 1H), 3.73 (s, 3H), 2.39 (td, *J* = 7.2, 3.1 Hz, 2H), 1.83 (dd, *J* = 12.6, 6.0 Hz, 1H), 1.76 – 1.63 (m, 3H), 1.44 (s, 9H).

^{13}C NMR (101 MHz, $CDCl_3$) δ 173.07, 172.87, 155.37, 135.92, 128.59, 128.28, 128.24, 79.98, 66.30, 53.10, 52.34, 33.60, 32.09, 28.32, 20.75.

HRMS (ESI-TOF) m/z: $[M+Na]^+$ calcd. for $C_{19}H_{27}NaNO_6$, 388.1731; found, 388.1713.

Chiral HPLC analysis conditions: column: CHIRALPAL OD-H, eluent: hexane/2-propanol = 19/1 (v/v), flow rate: 1.0 ml/min, room temperature.

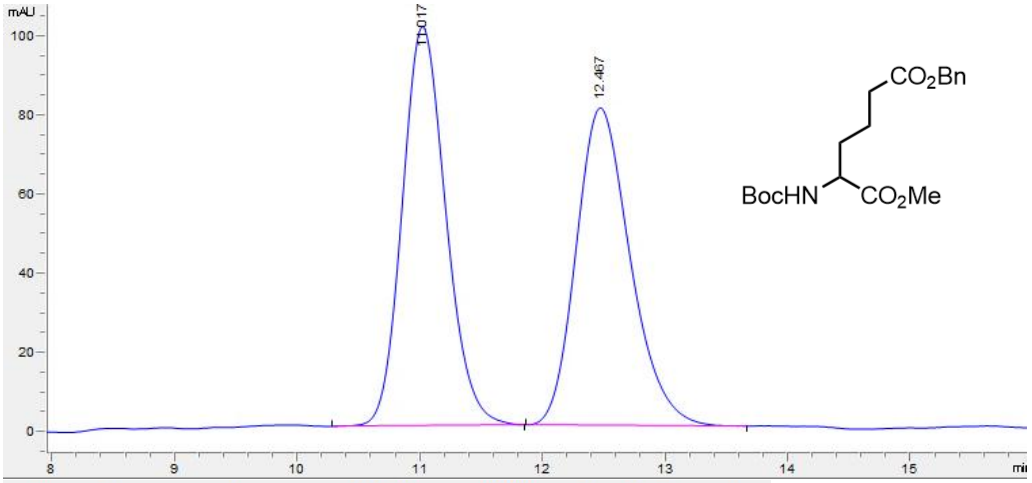

#	Time	Area	Height	Width	Area%	Symmetry
1	12.259	4477.7	171.3	0.4049	50.192	0.807
2	13.885	4443.5	146.7	0.4593	49.808	0.8

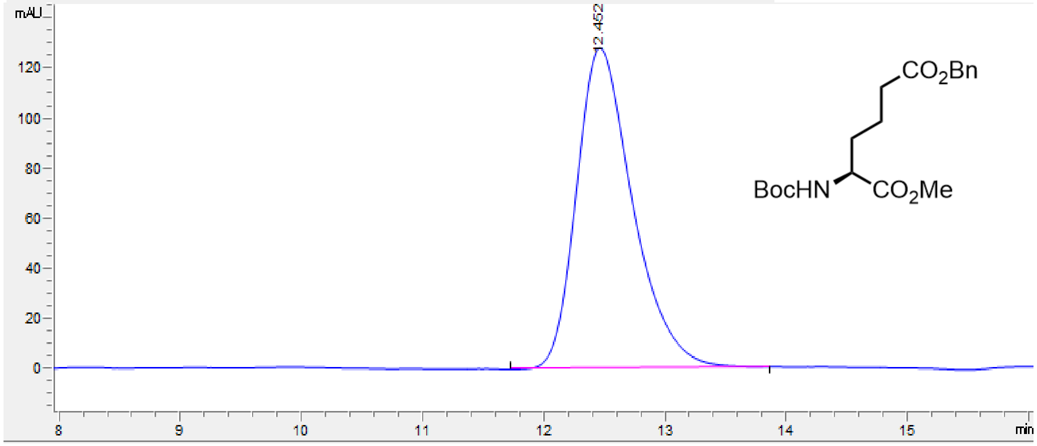

Figure S22. Comparison of **18** and **S4**

12. NMR spectra for New Compounds

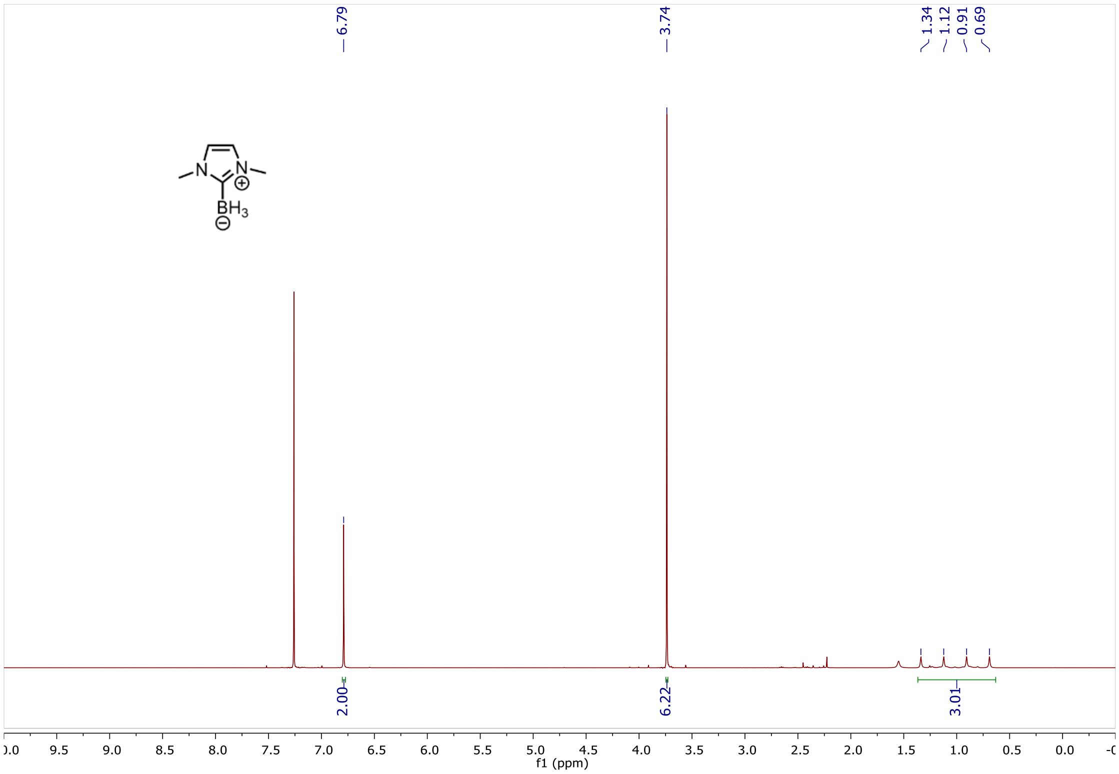

Figure S23. ^{1}H NMR (400 MHz, $CDCl_3$) of **8**

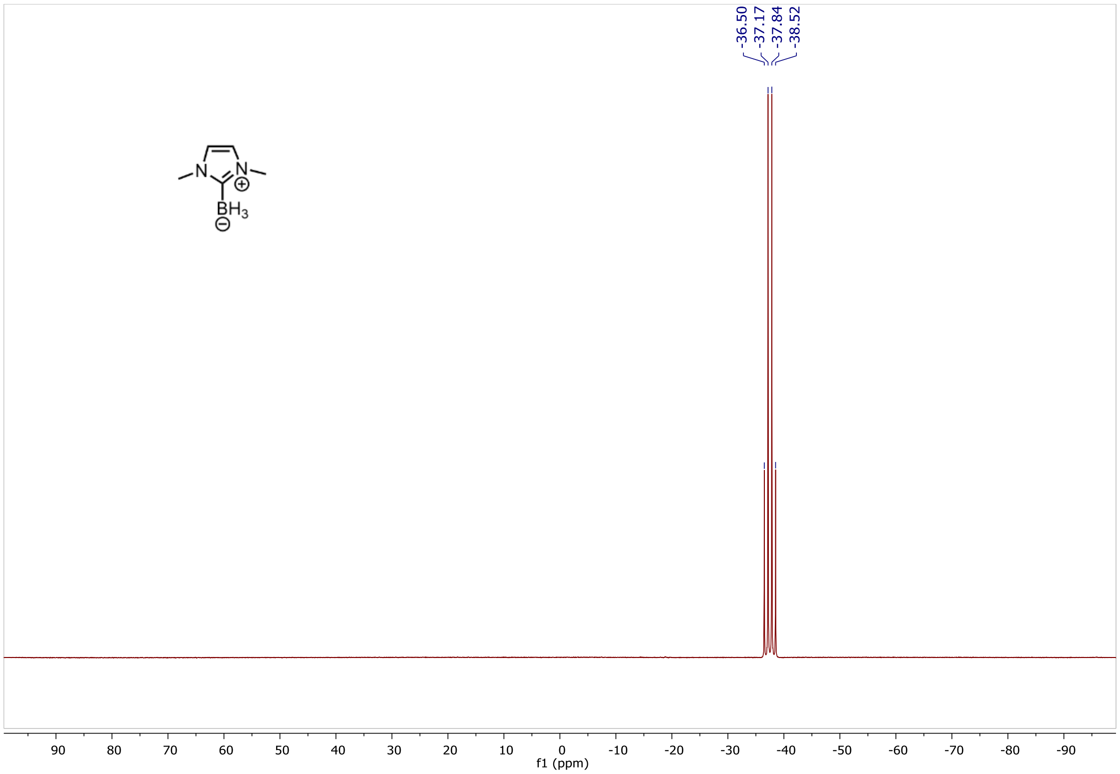

Figure S24. ^{11}B NMR (128 MHz, $CDCl_3$) of **8**

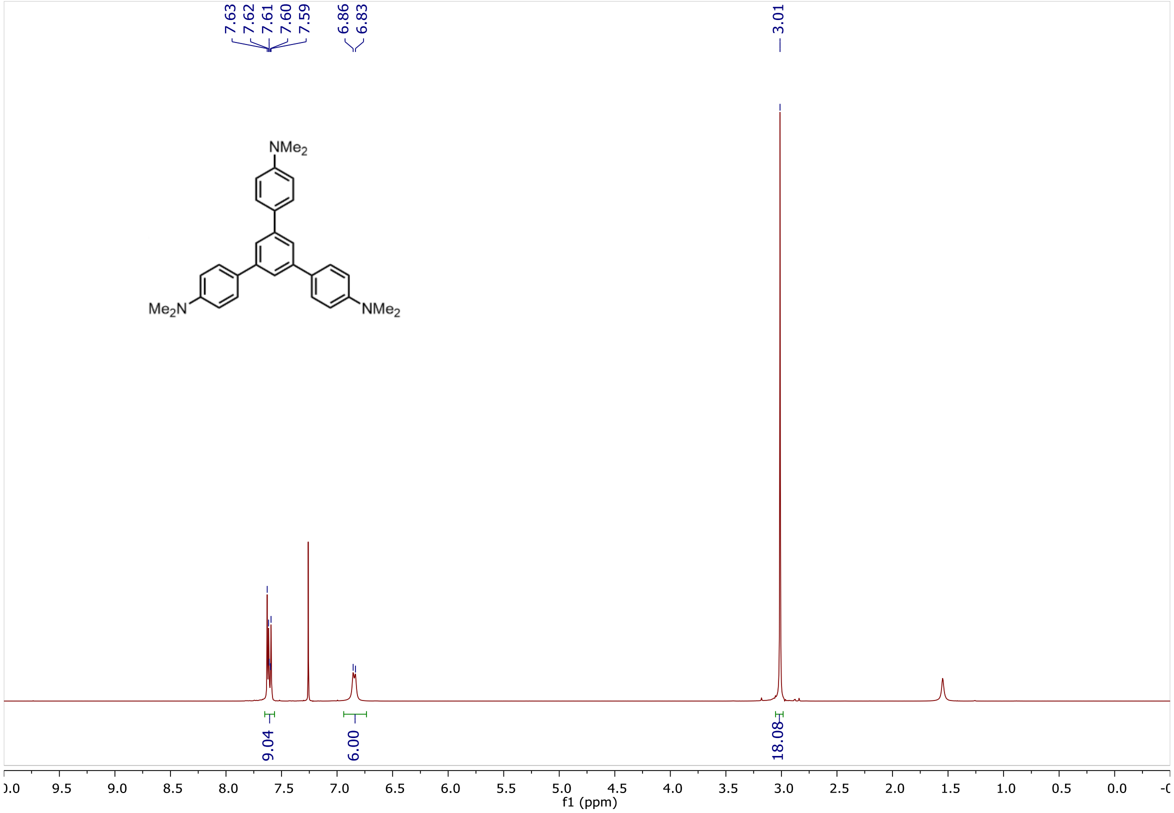

Figure S25. 1H NMR (400 MHz, $CDCl_3$) of **6**

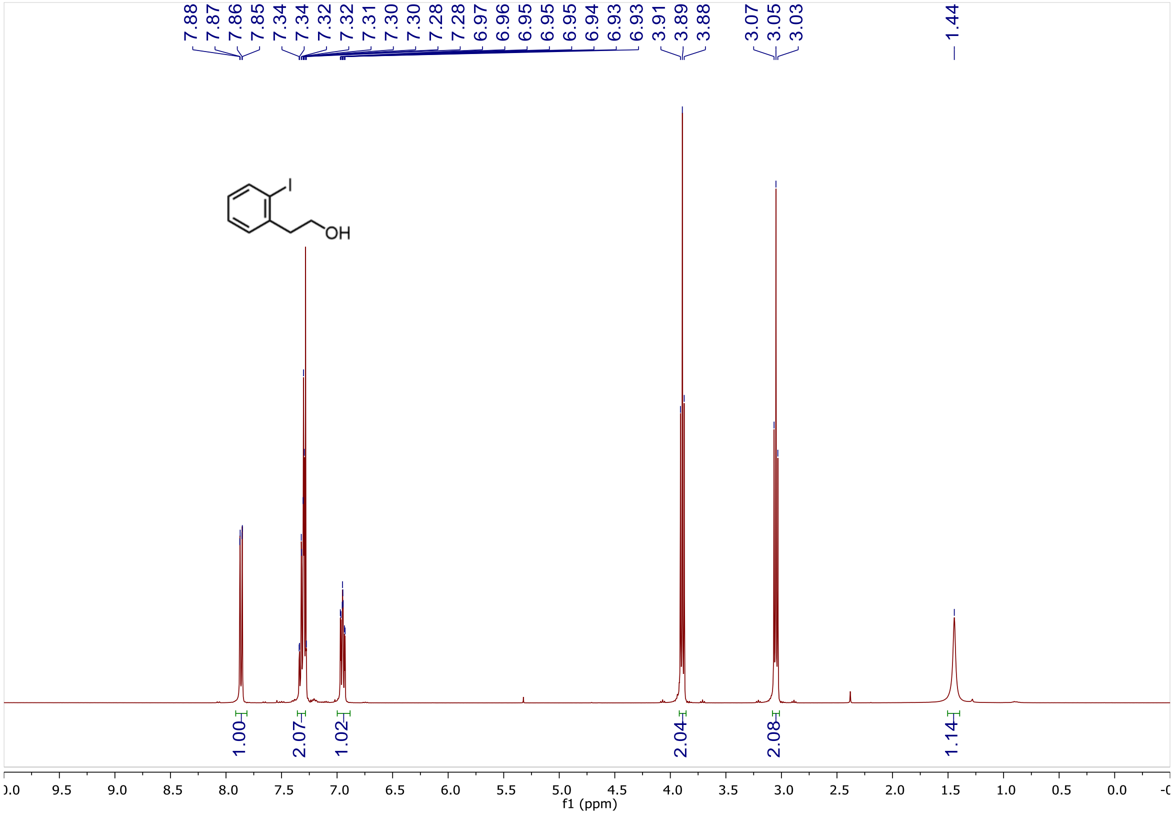

Figure S26. 1H NMR (400 MHz, $CDCl_3$) of **S1**

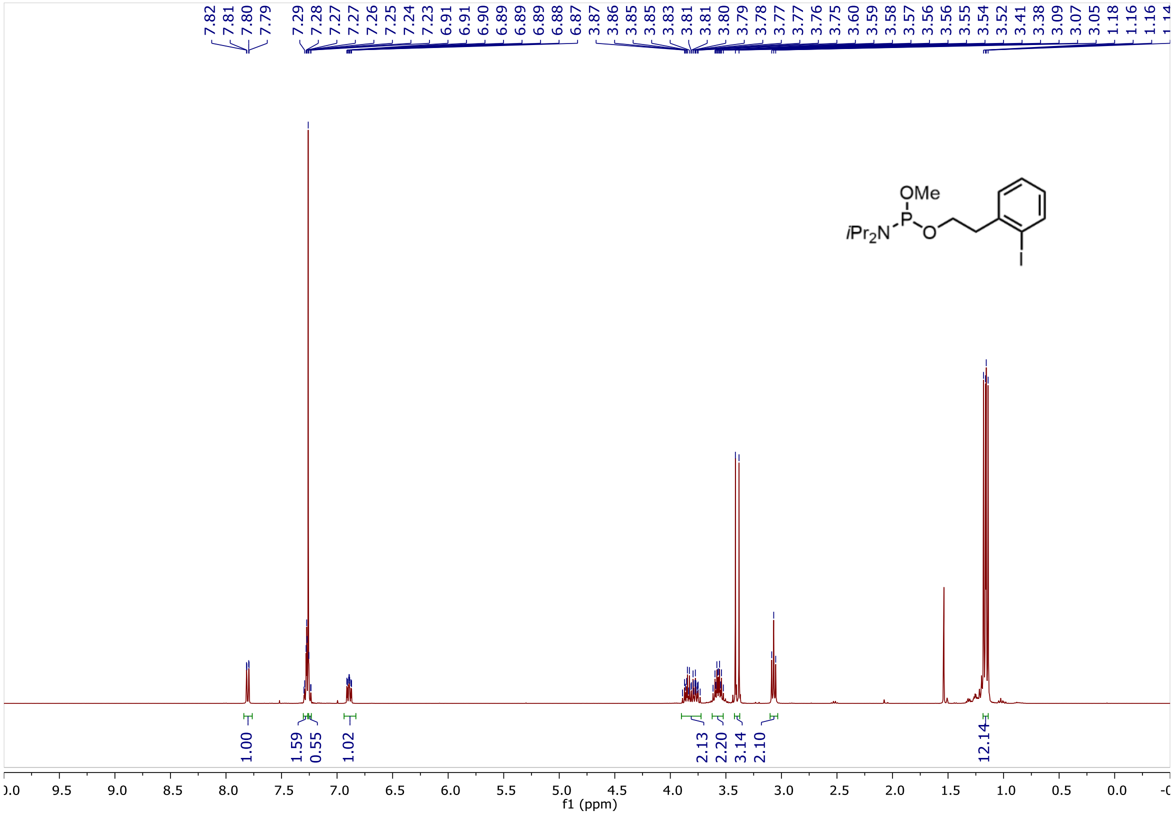

Figure S27. ^{1}**H NMR** (400 MHz, $CDCl_3$) of **1**

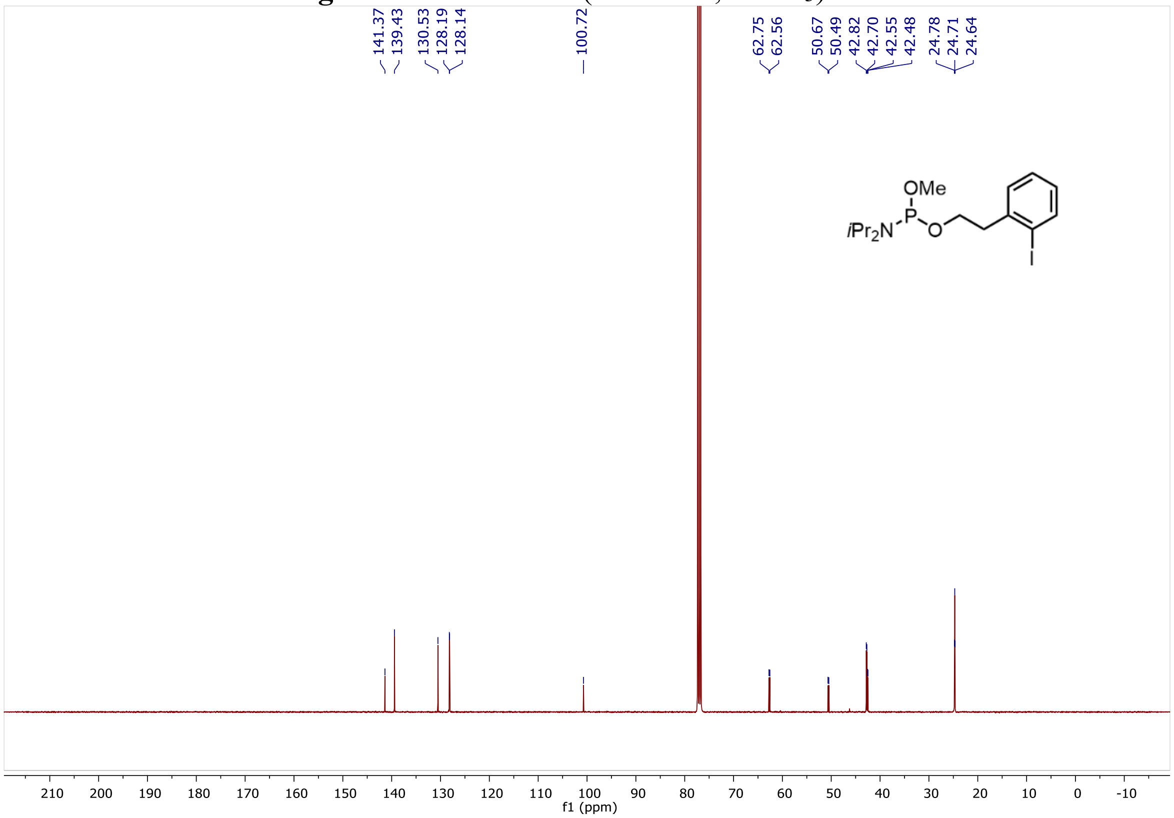

Figure S28. ^{13}**C NMR** (101 MHz, $CDCl_3$) of **1**

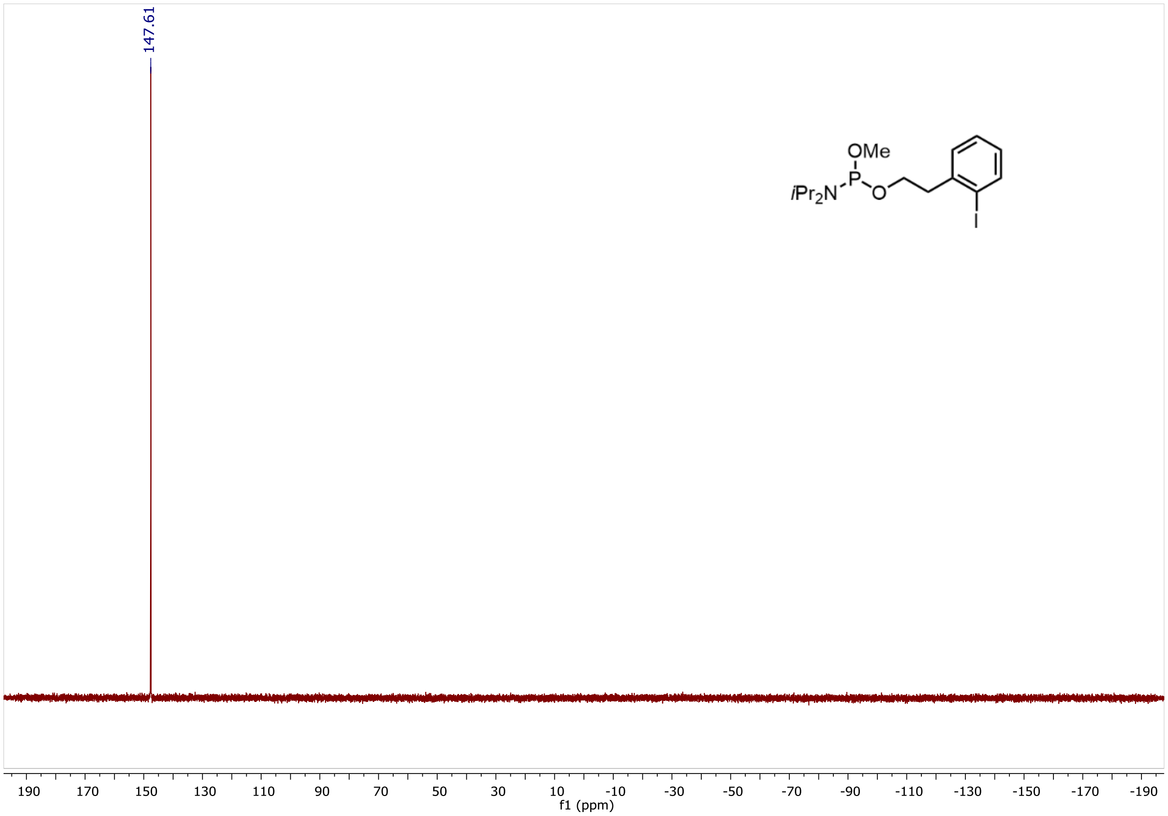

Figure S29. ^{31}**P NMR** (162 MHz, $CDCl_3$) of **1**

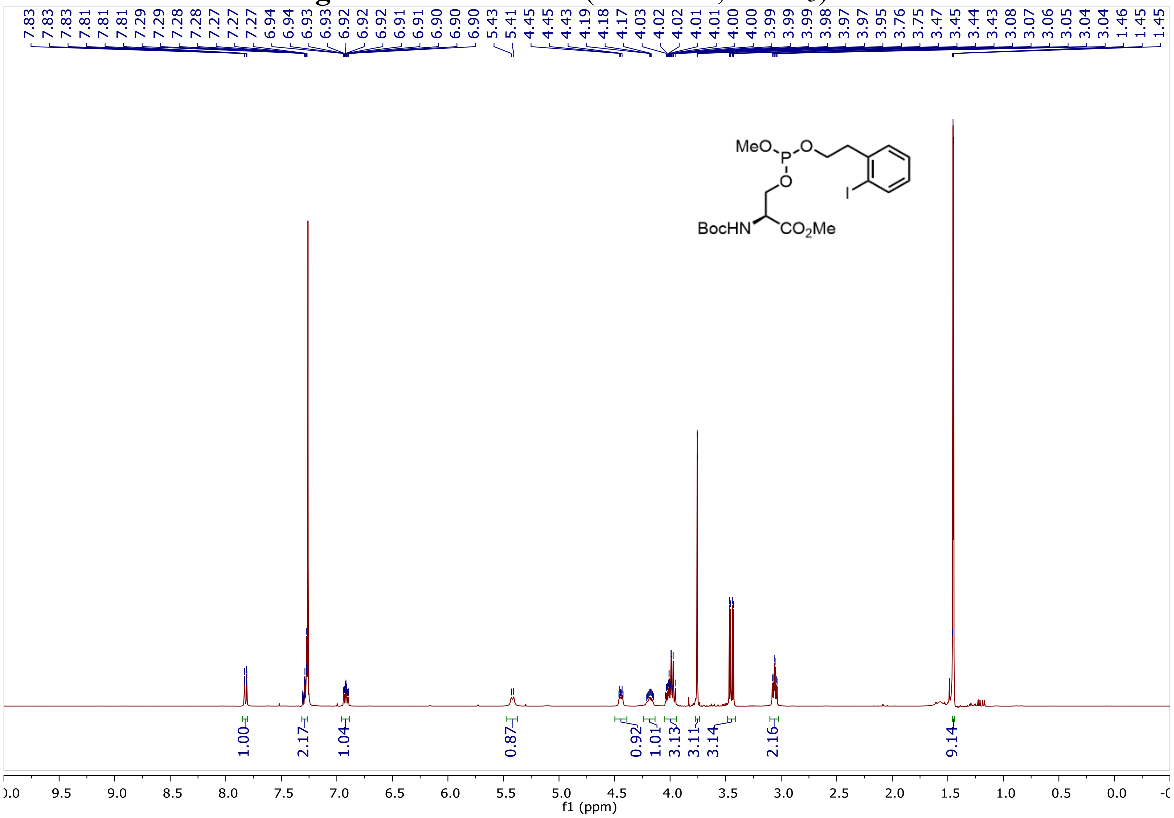

Figure S30. ^{1}**H NMR** (400 MHz, $CDCl_3$) of **3**

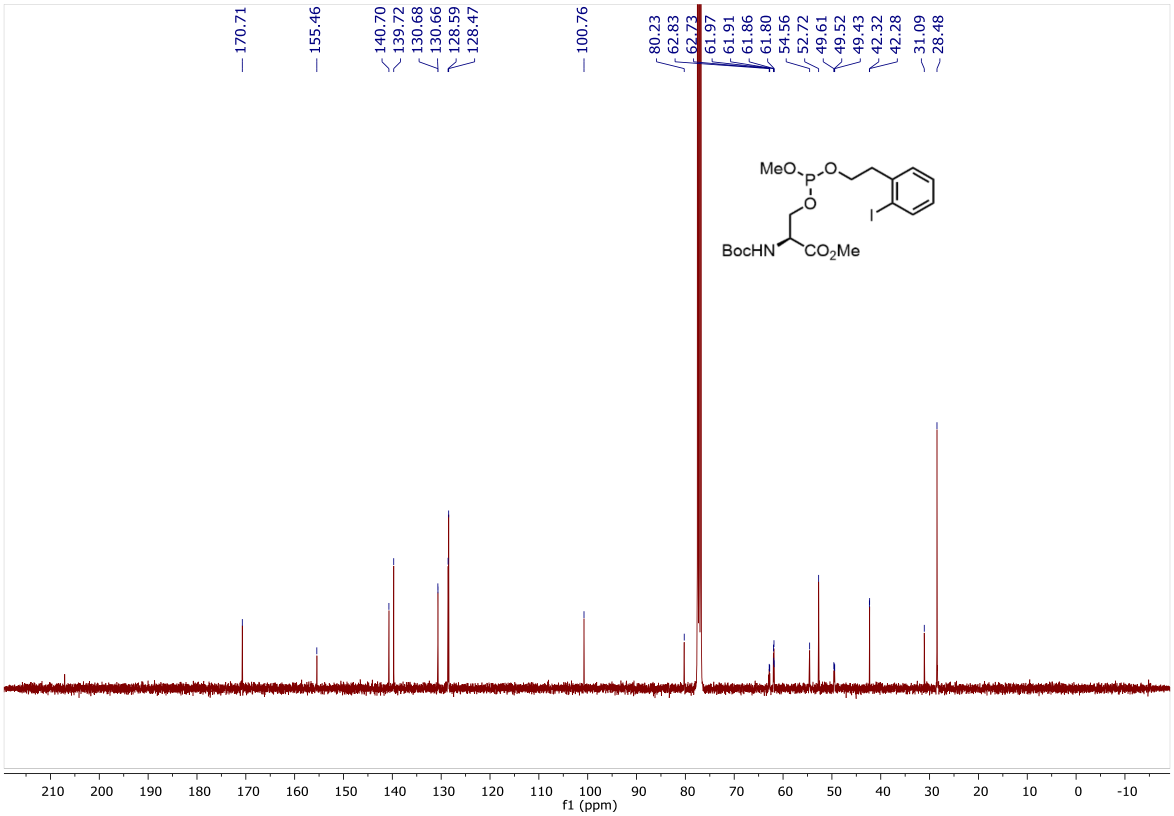

Figure S31. ^{13}C NMR (101 MHz, $CDCl_3$) of **3**

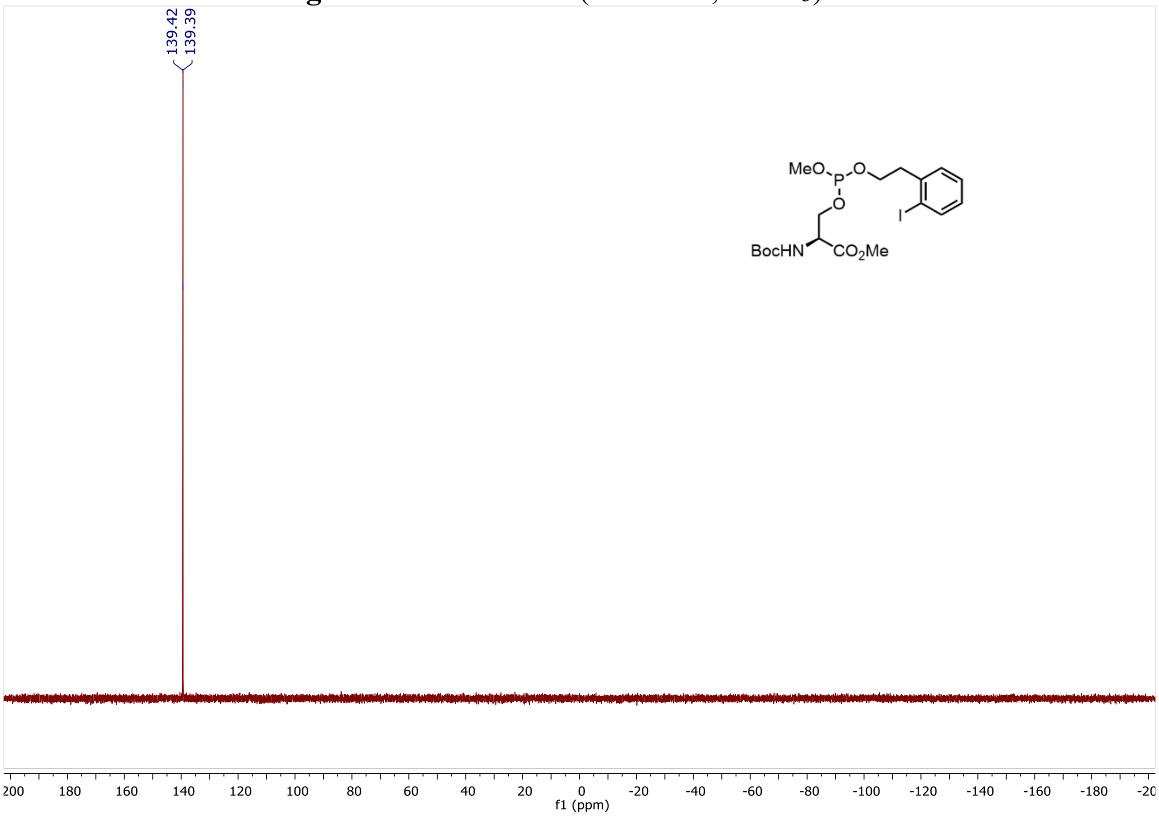

Figure S32. ^{31}P NMR (162 MHz, $CDCl_3$) of **3**

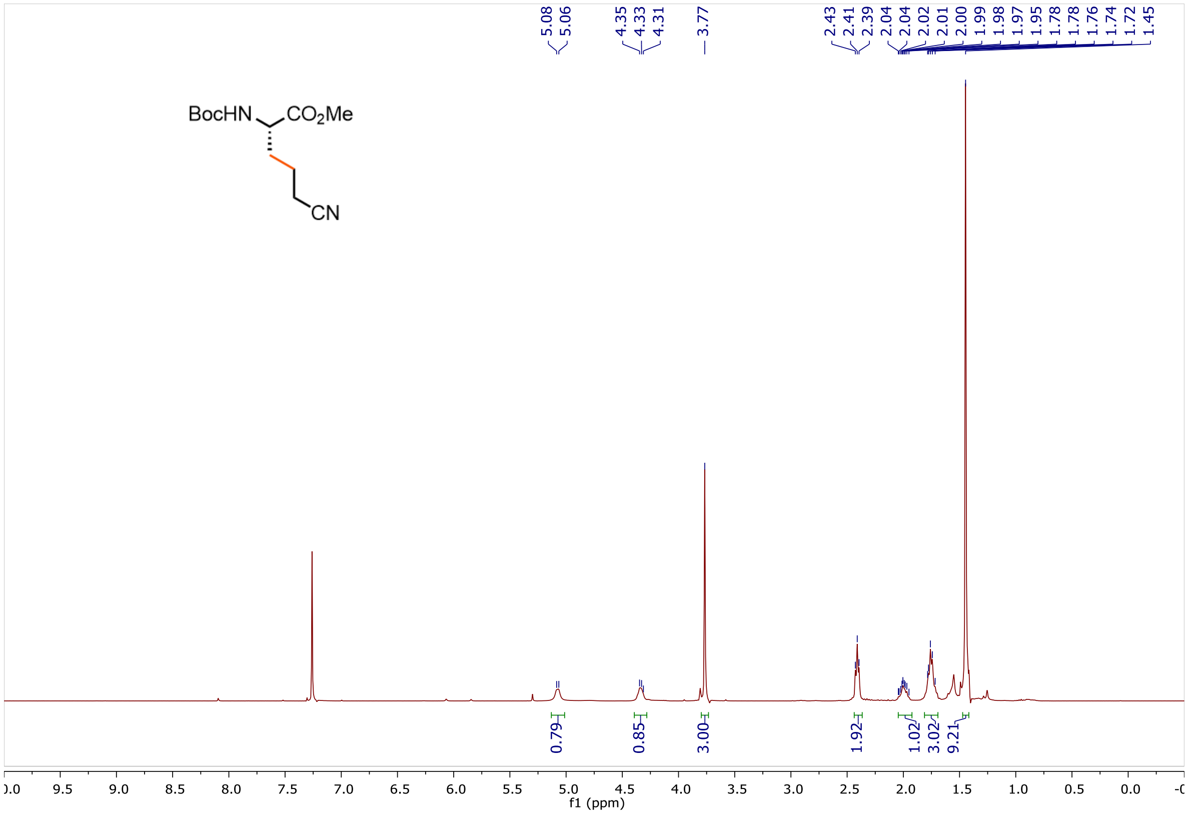

Figure S33. ^{1}**H NMR** (400 MHz, $CDCl_3$) of **4**

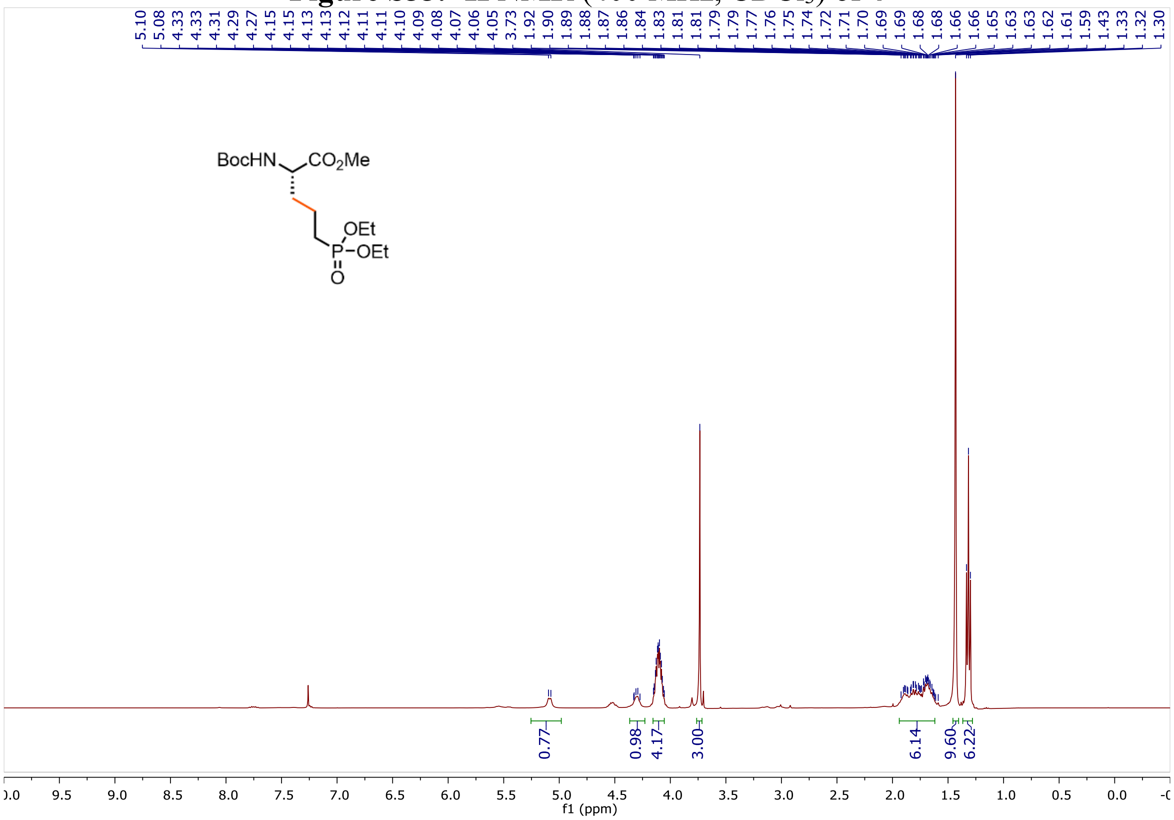

Figure S34. ^{1}**H NMR** (400 MHz, $CDCl_3$) of **15**

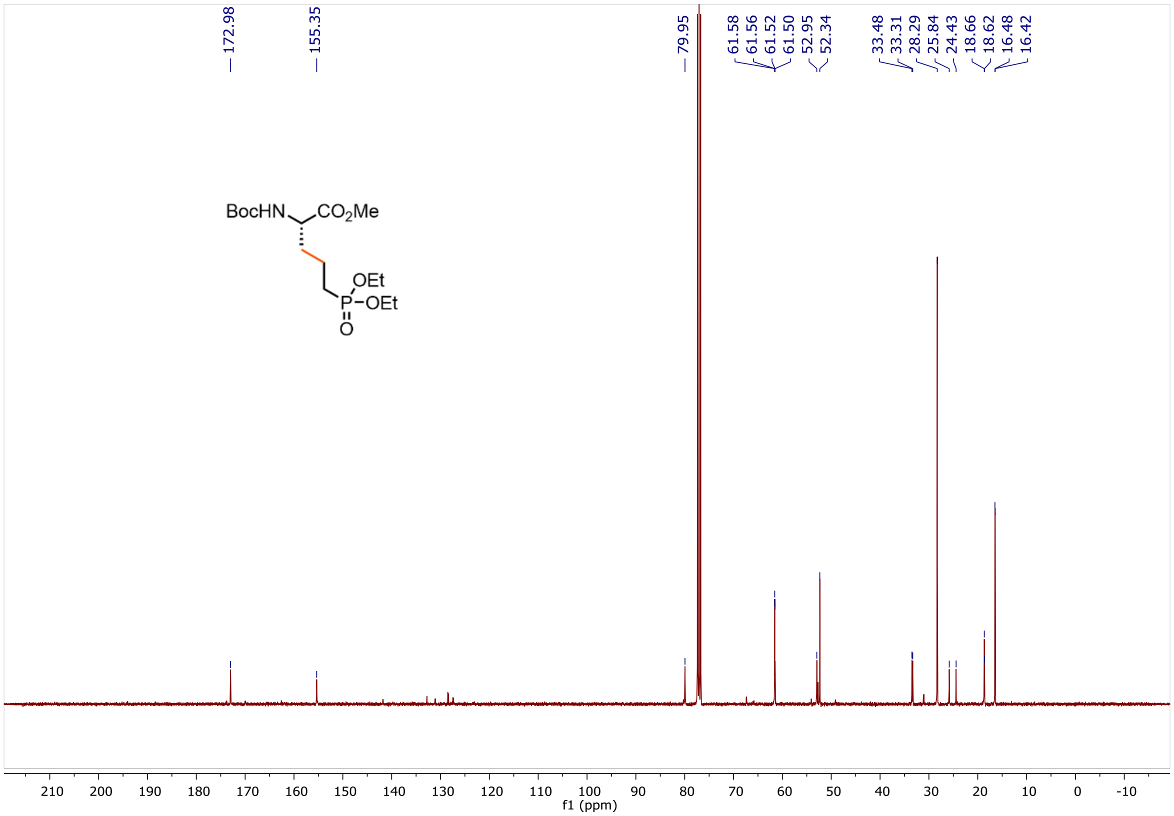

Figure S35. ^{13}**C NMR** (101 MHz, $CDCl_3$) of **15**

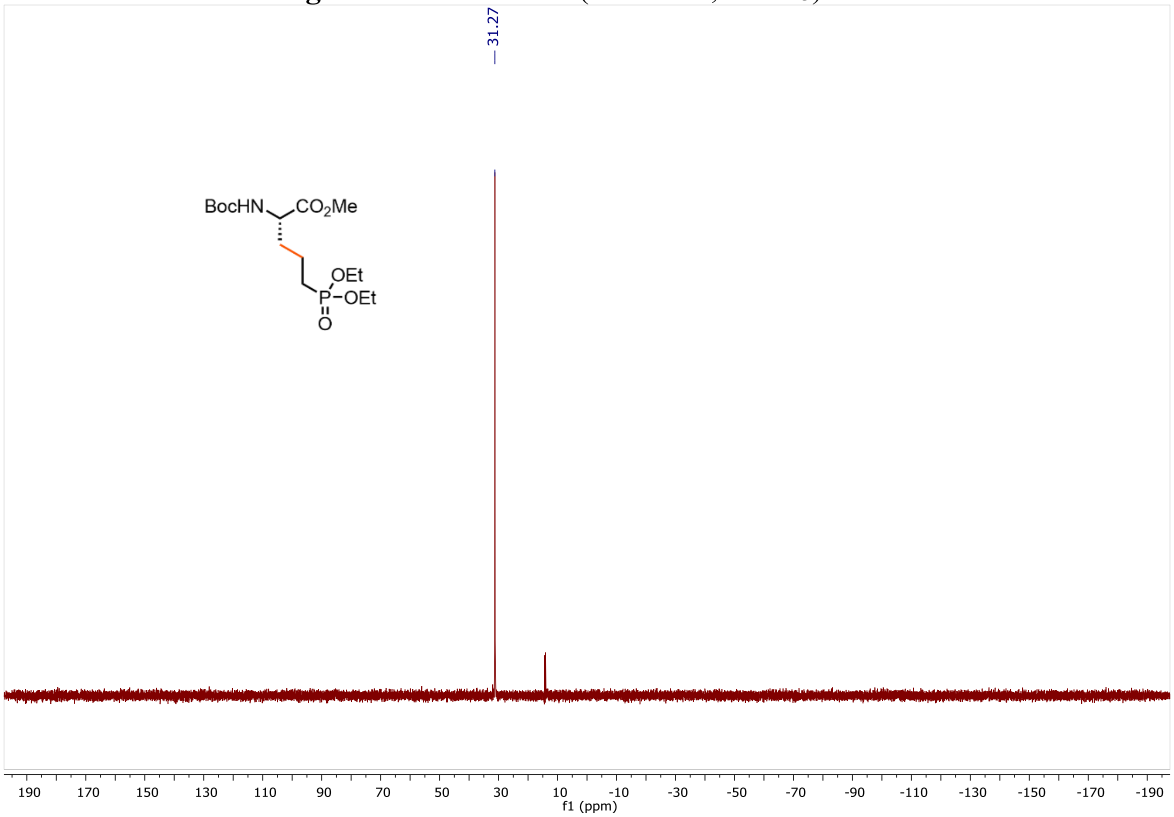

Figure S36. ^{31}**P NMR** (162 MHz, $CDCl_3$) of **15**

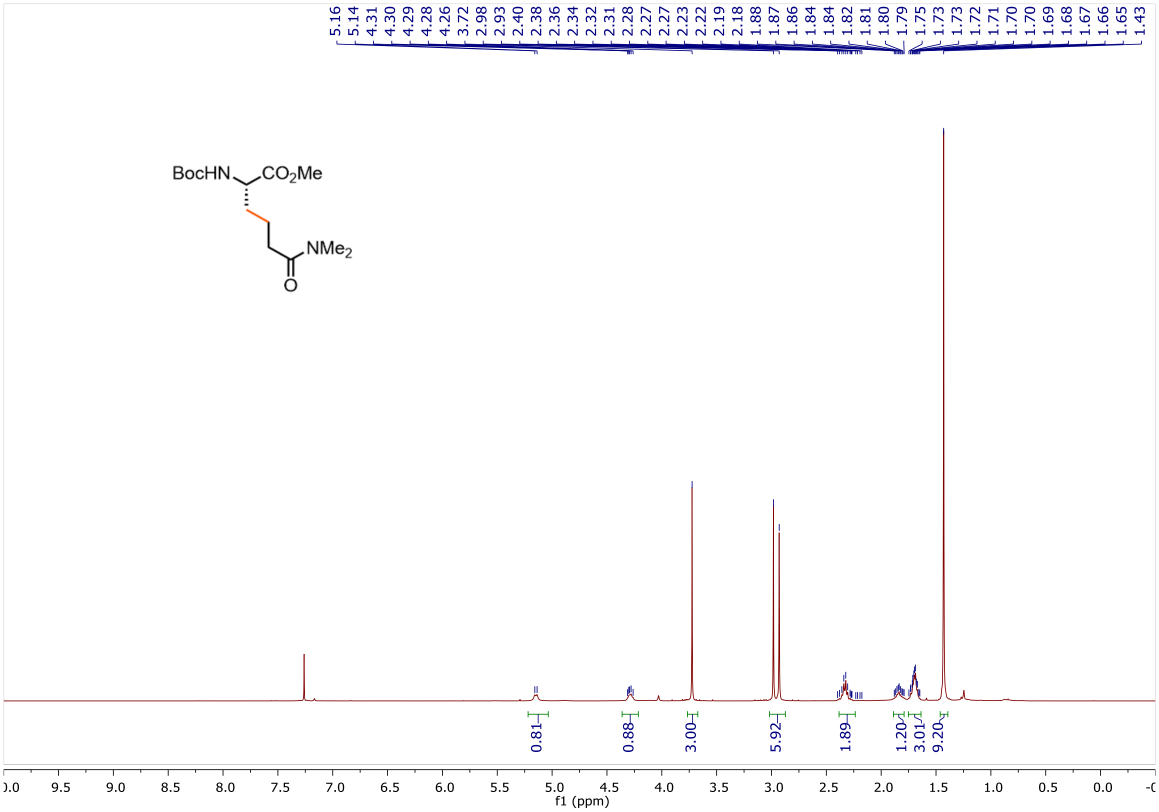

Figure S37. ^{1}**H NMR** (600 MHz, $CDCl_3$) of **16**

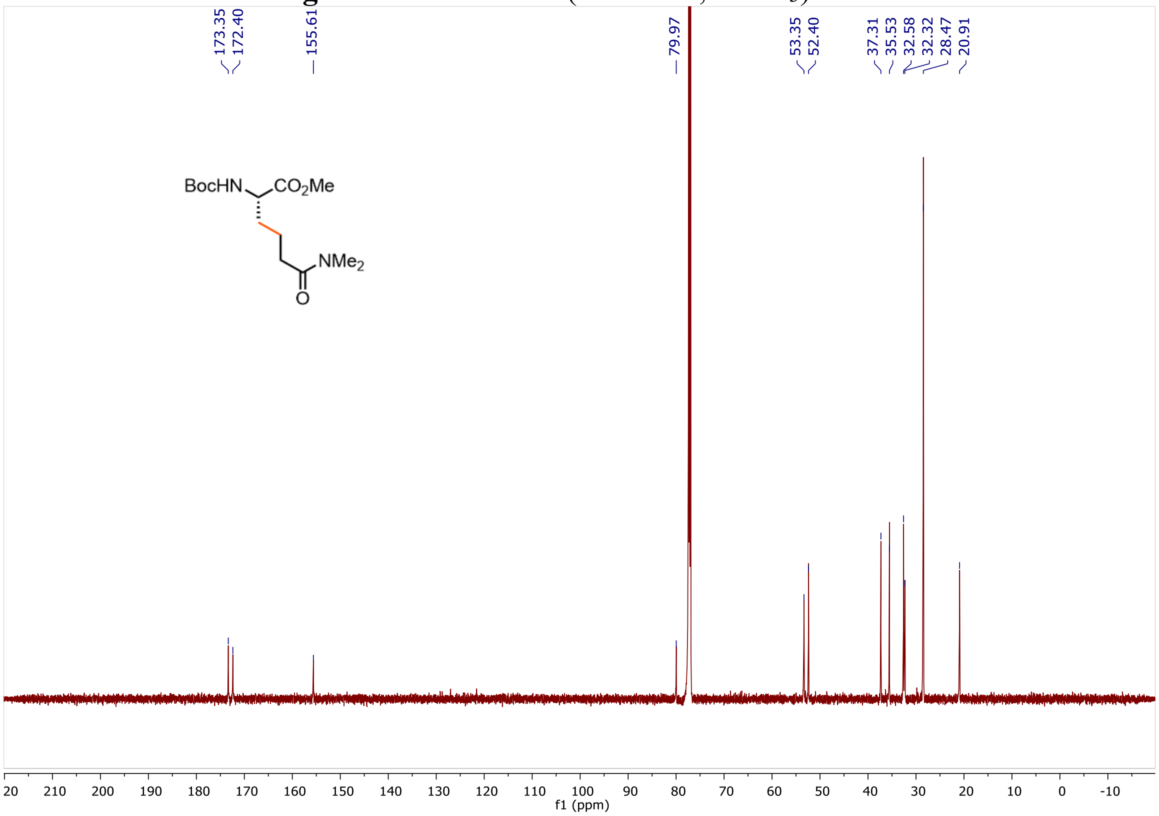

Figure S38. ^{13}**C NMR** (151 MHz, $CDCl_3$) of **16**

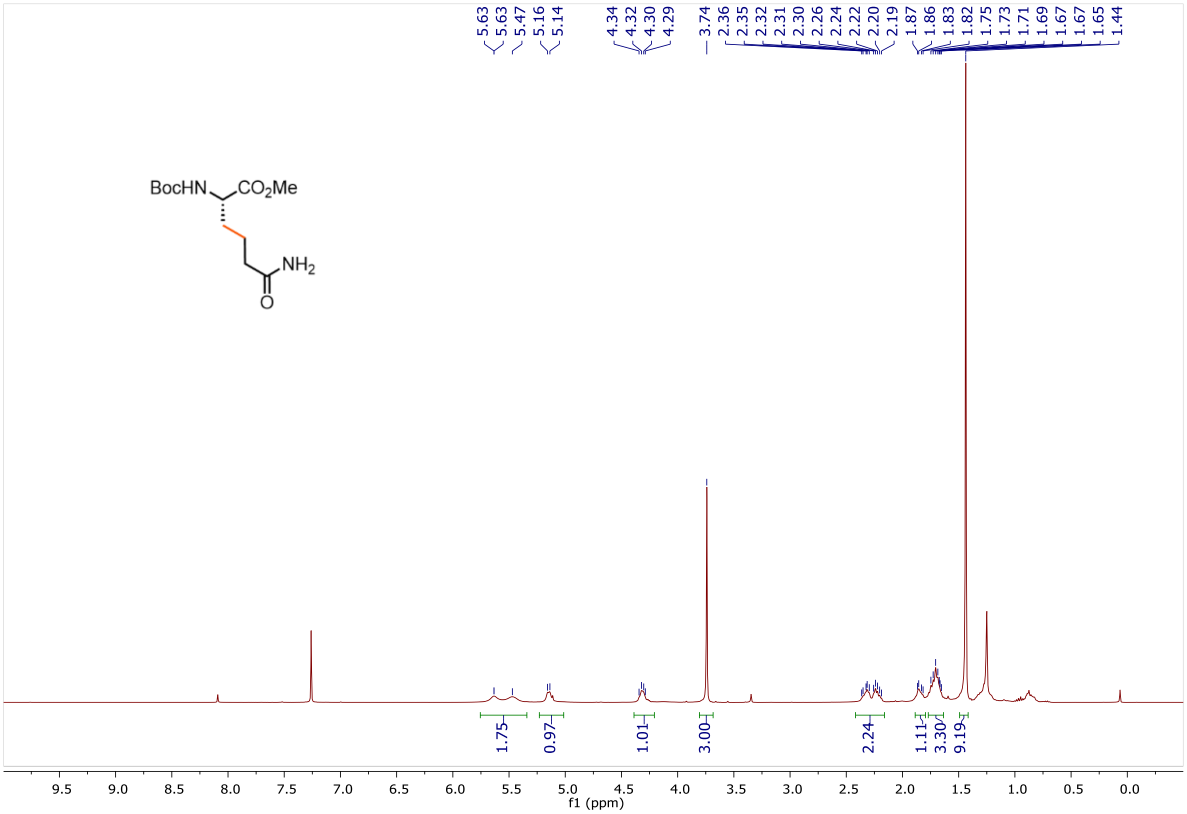

Figure S39. ^{1}**H NMR** (400 MHz, $CDCl_3$) of **17**

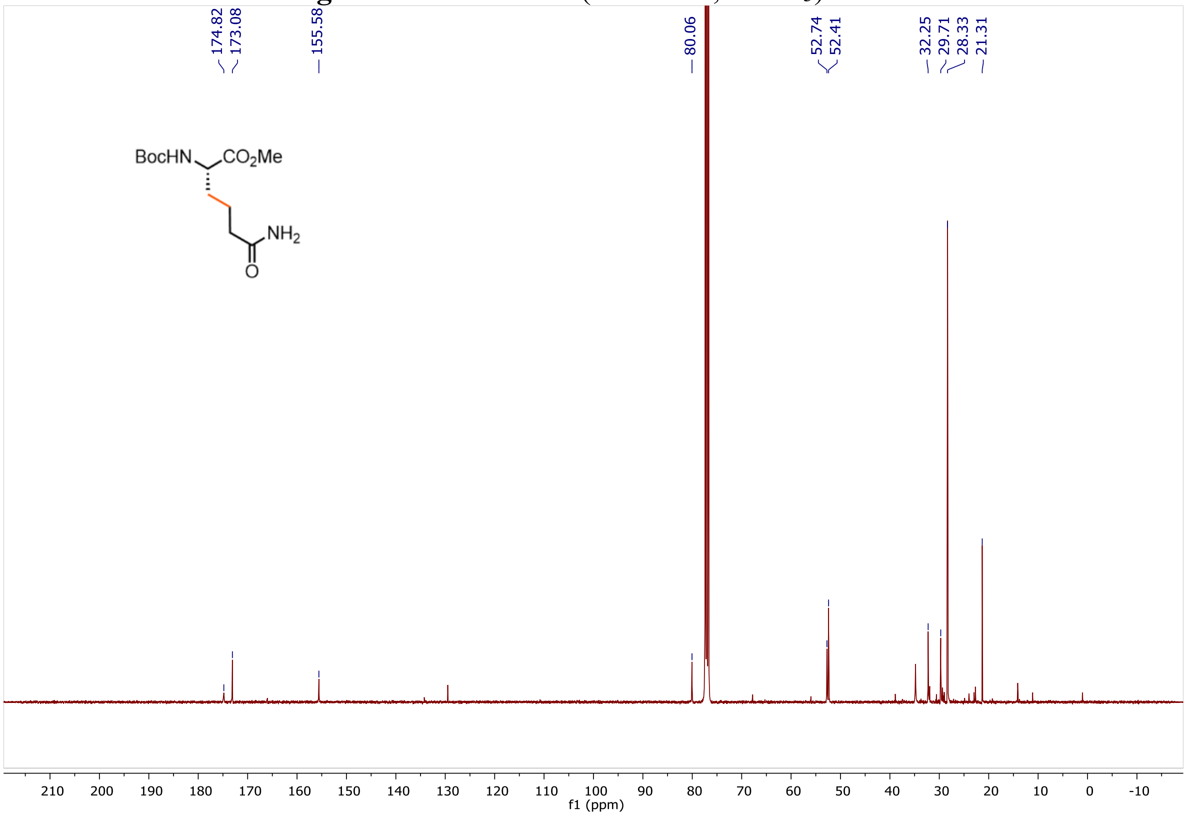

Figure S40. ^{13}**C NMR** (101 MHz, $CDCl_3$) of **17**

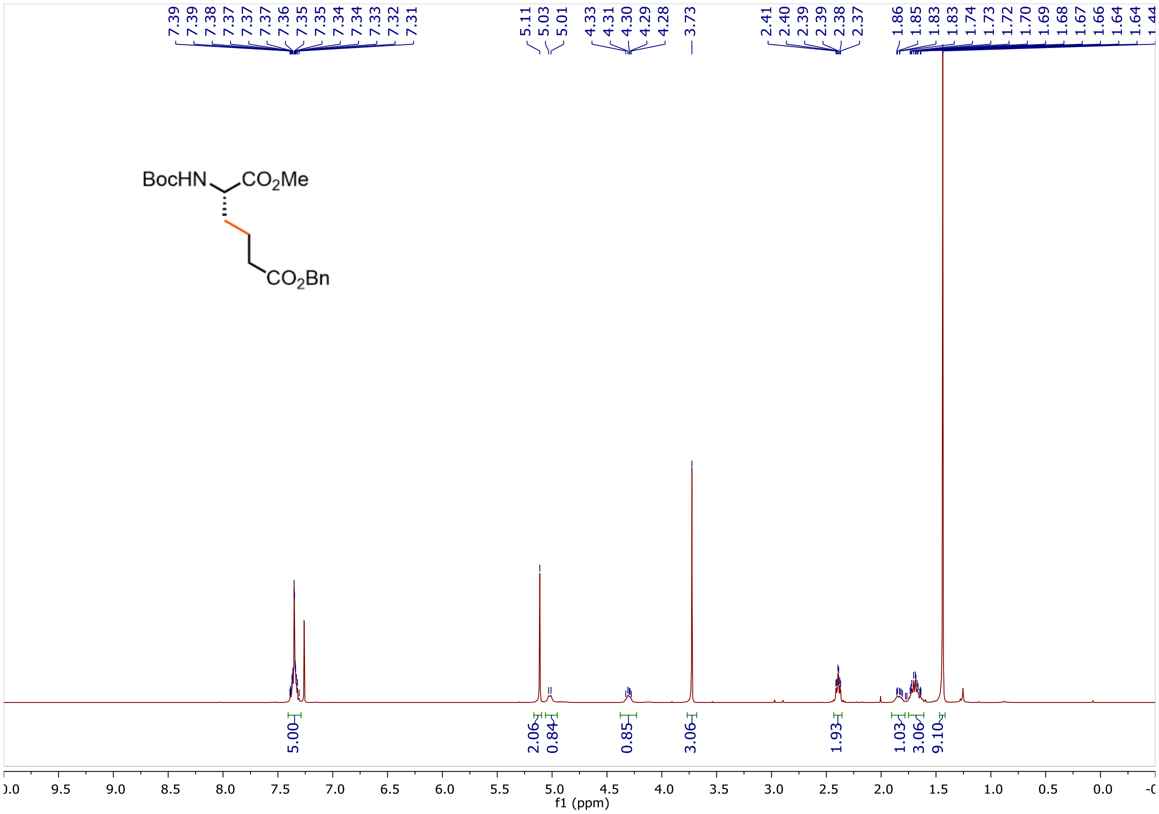

Figure S41. ^{1}**H NMR** (400 MHz, $CDCl_3$) of **18**

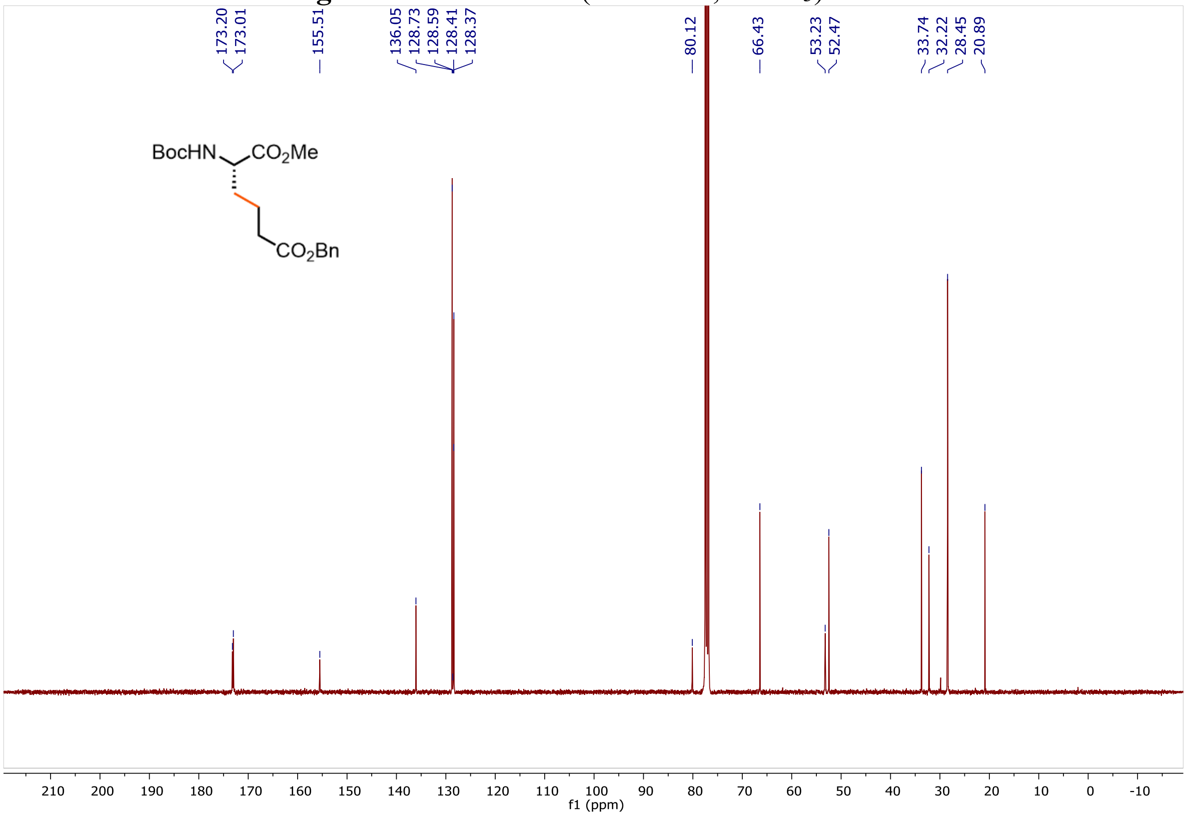

Figure S42. ^{13}**C NMR** (101 MHz, $CDCl_3$) of **18**

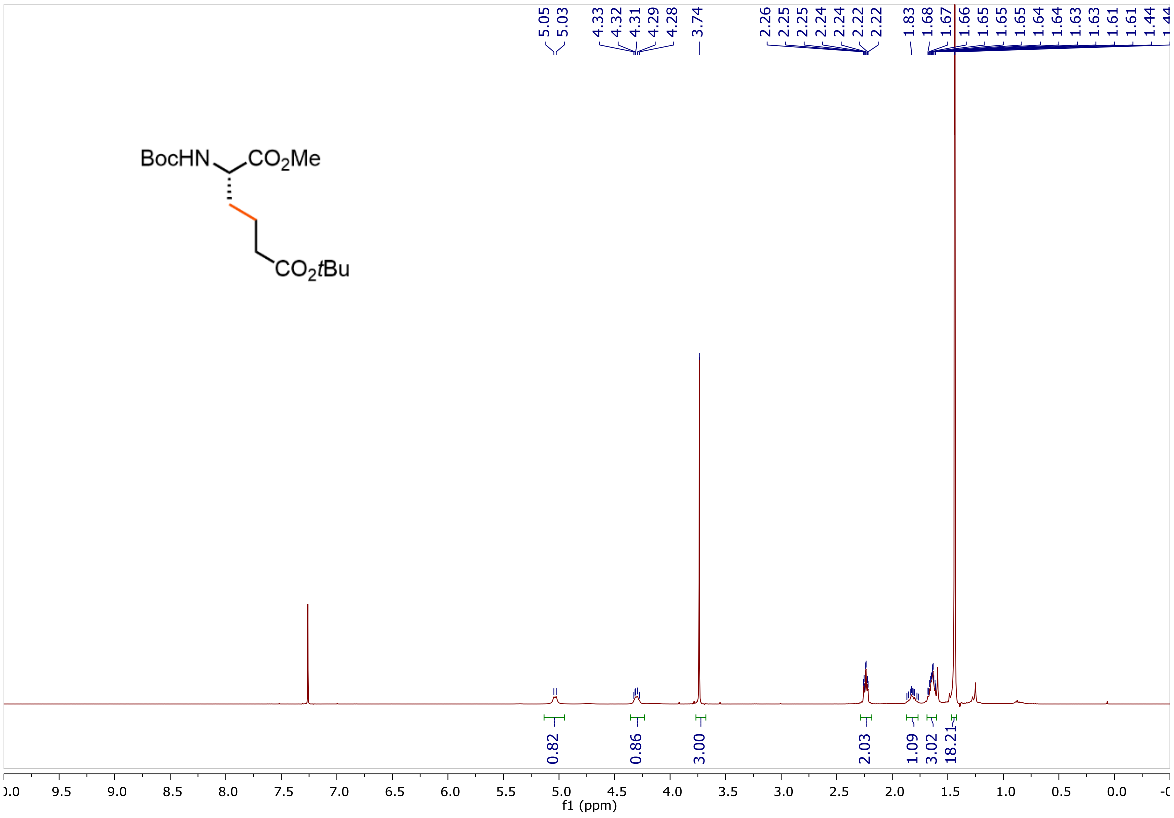

Figure S43. ^{1}**H NMR** (400 MHz, $CDCl_3$) of **19**

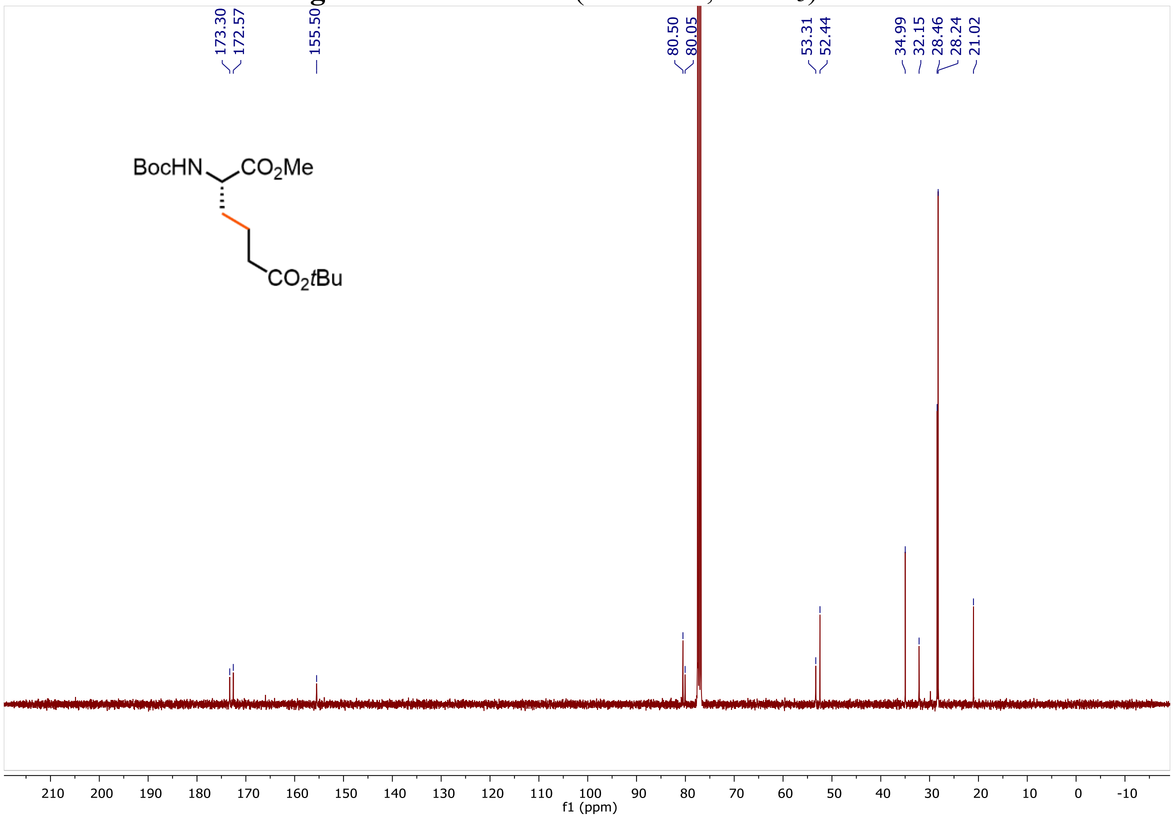

Figure S44. ^{13}**C NMR** (101 MHz, $CDCl_3$) of **19**

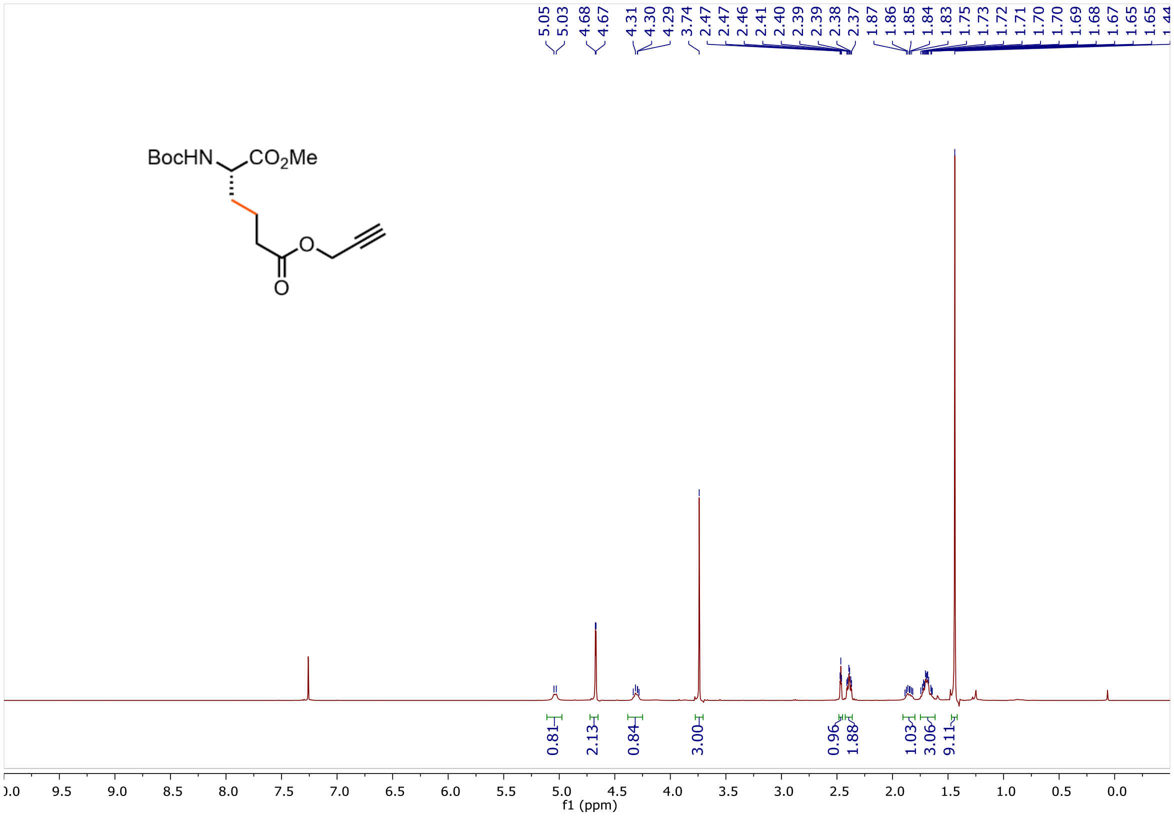

Figure S45. ^{1}**H NMR** (400 MHz, $CDCl_3$) of **20**

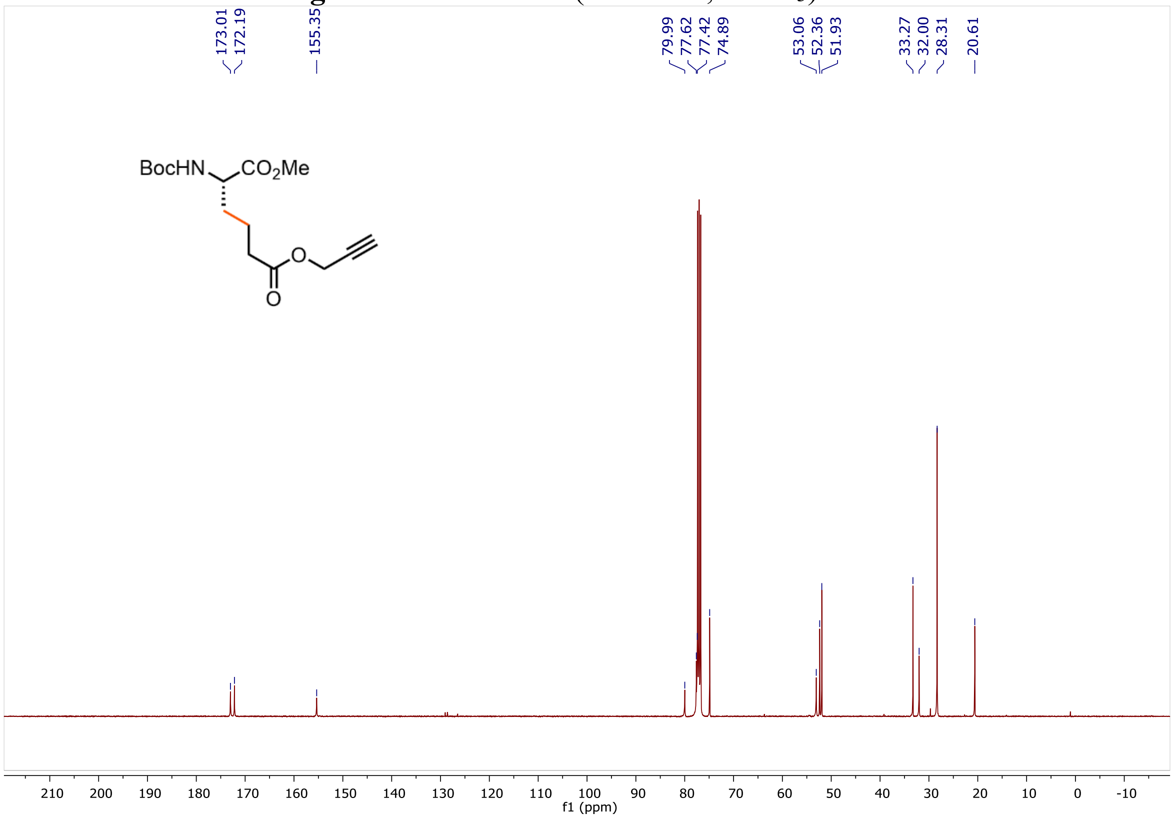

Figure S46. ^{13}**C NMR** (101 MHz, $CDCl_3$) of **20**

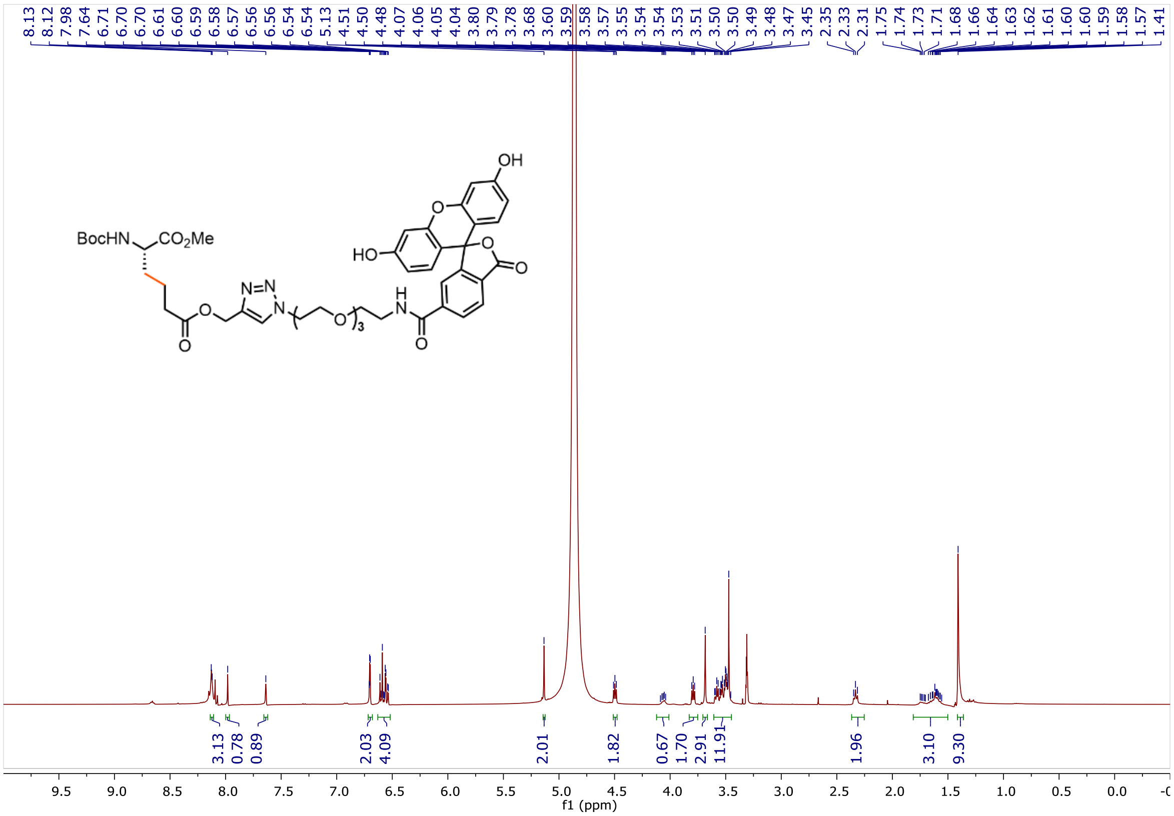

Figure S47. ^{1}**H NMR** (400 MHz, MeOD) of **21**

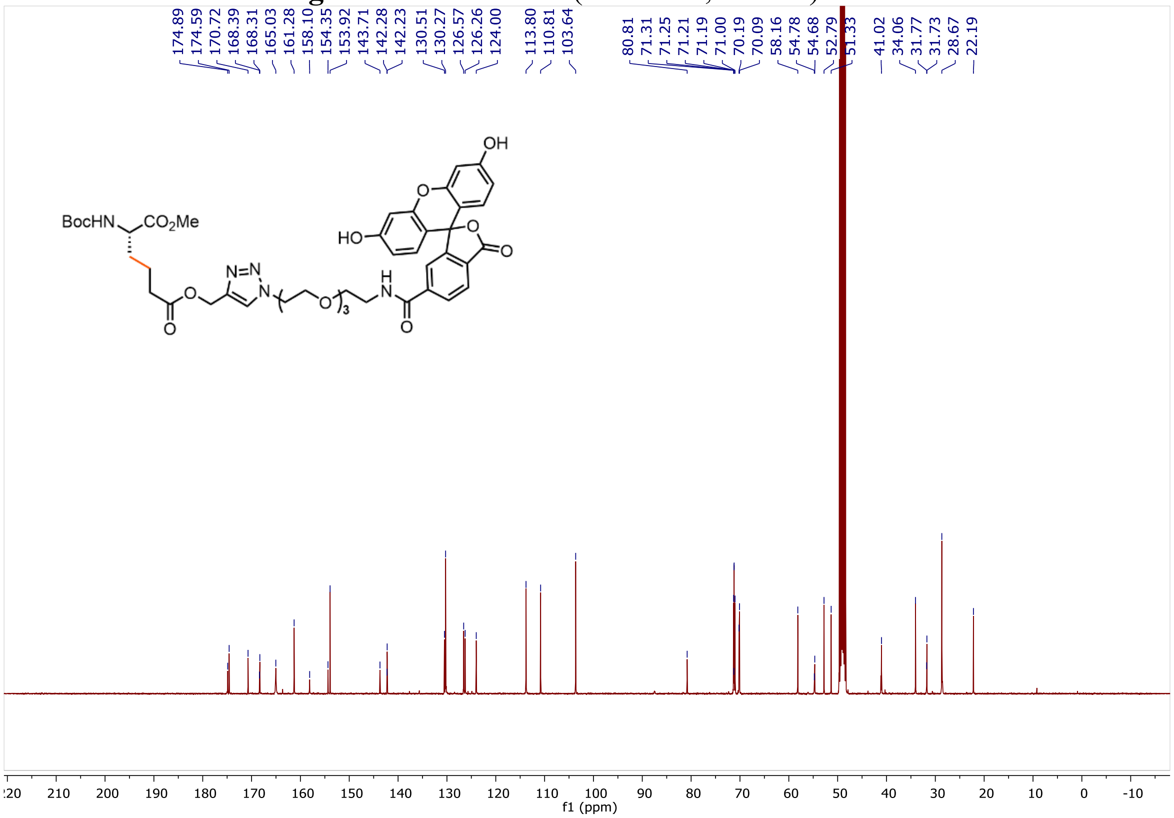

Figure S48. ^{13}**C NMR** (101 MHz, MeOD) of **21**

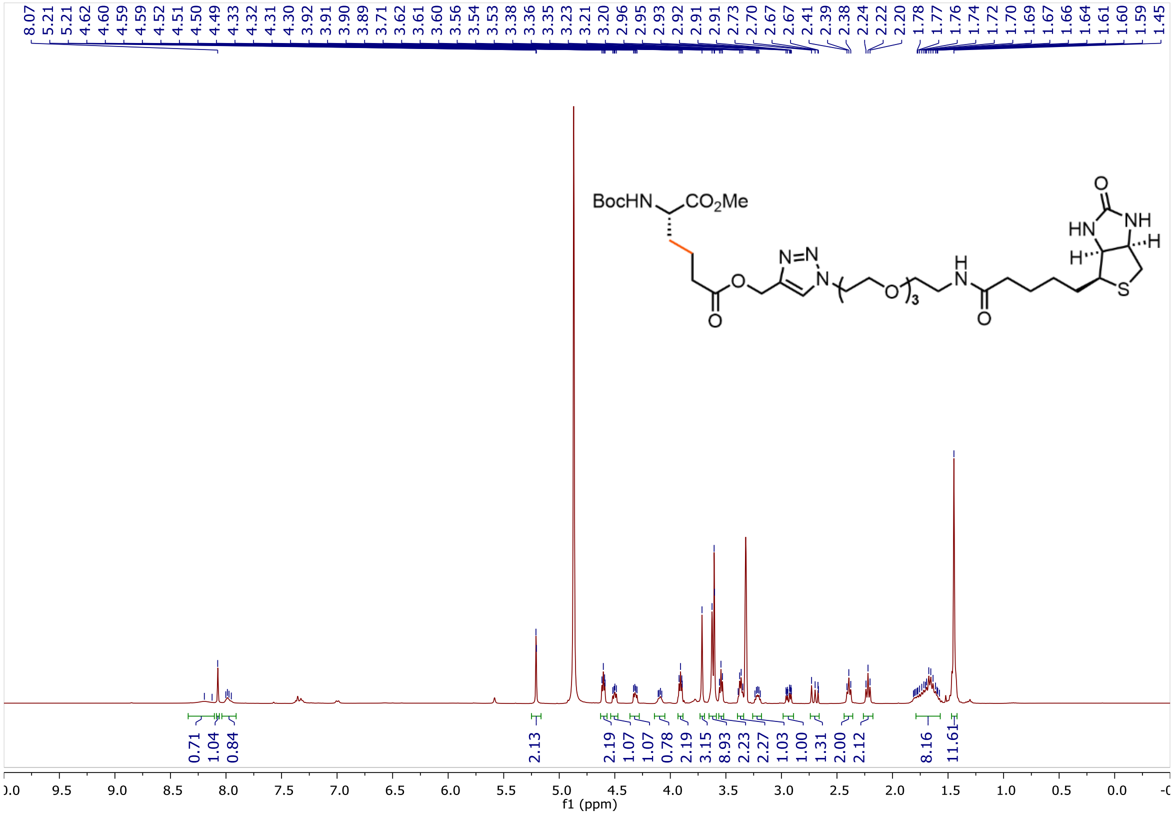

Figure S49. ^{1}H NMR (400 MHz, MeOD) of **22**

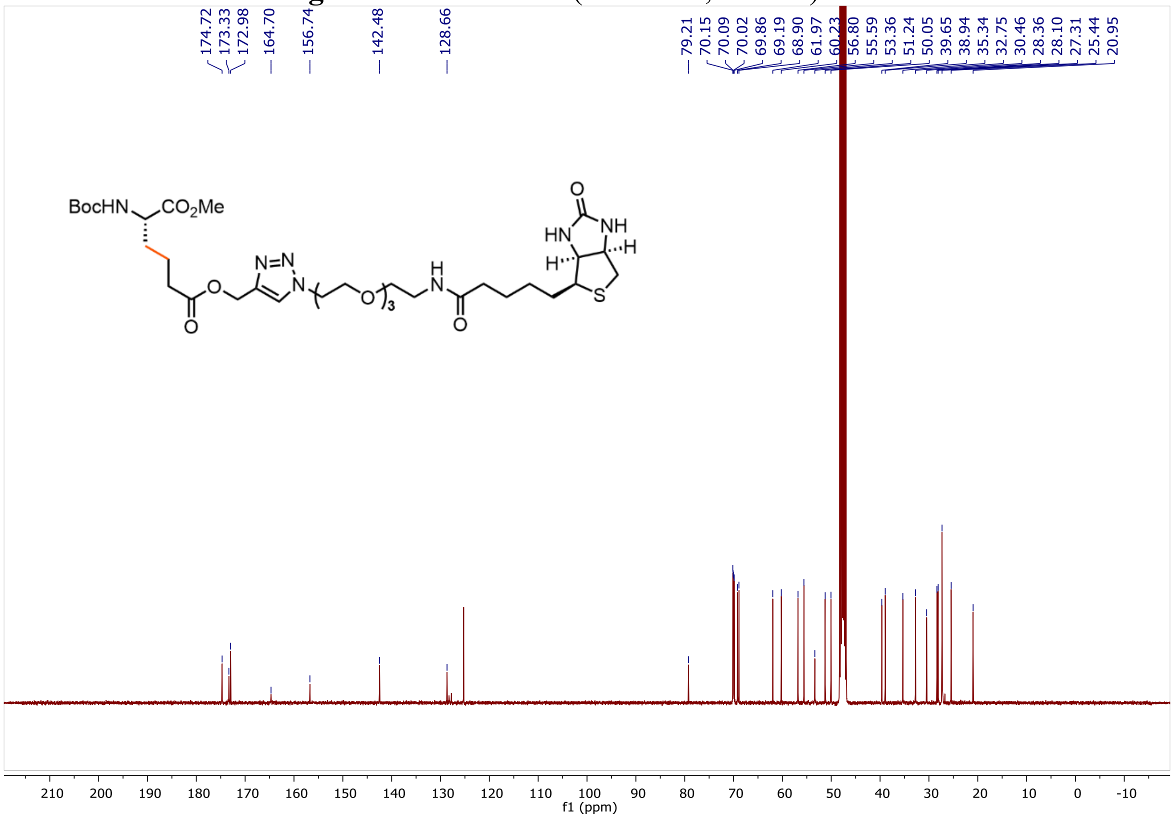

Figure S50. ^{13}C NMR (101 MHz, MeOD) of **22**

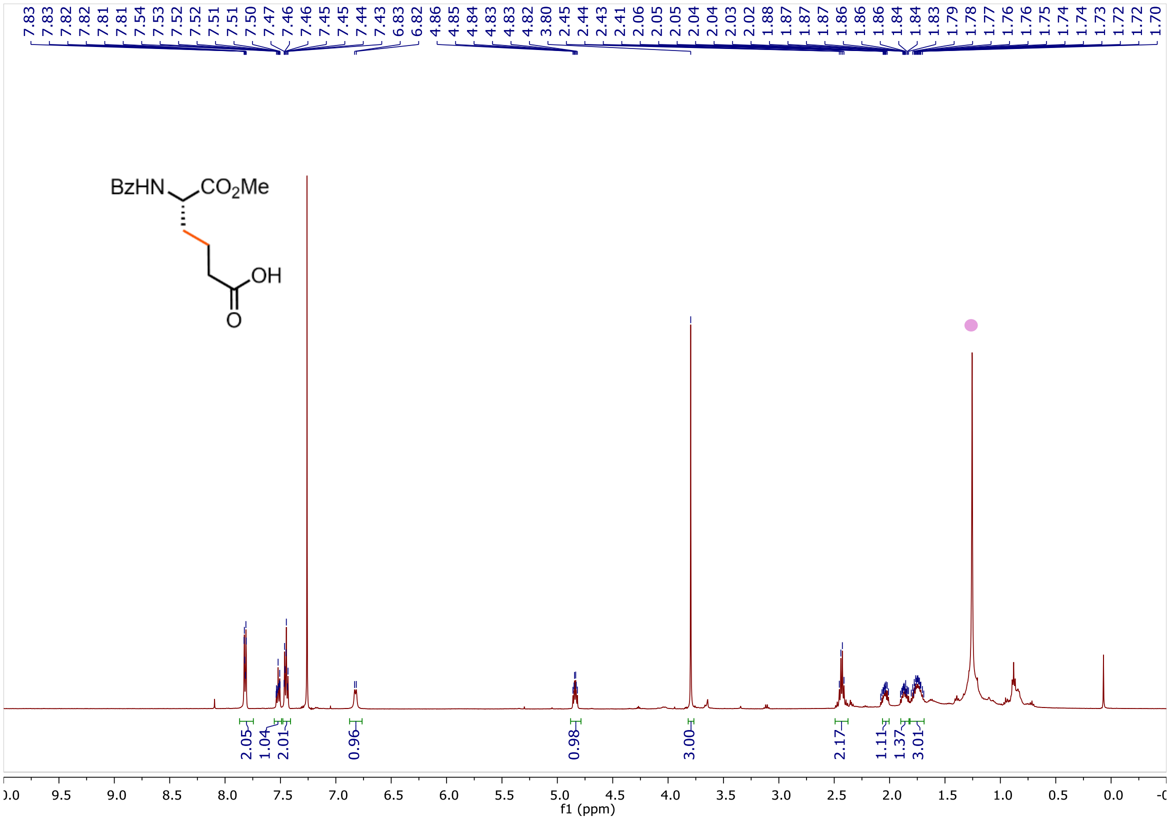

Figure S51. **^{1}H NMR** (400 MHz, $CDCl_3$) of **23** (●) H grease

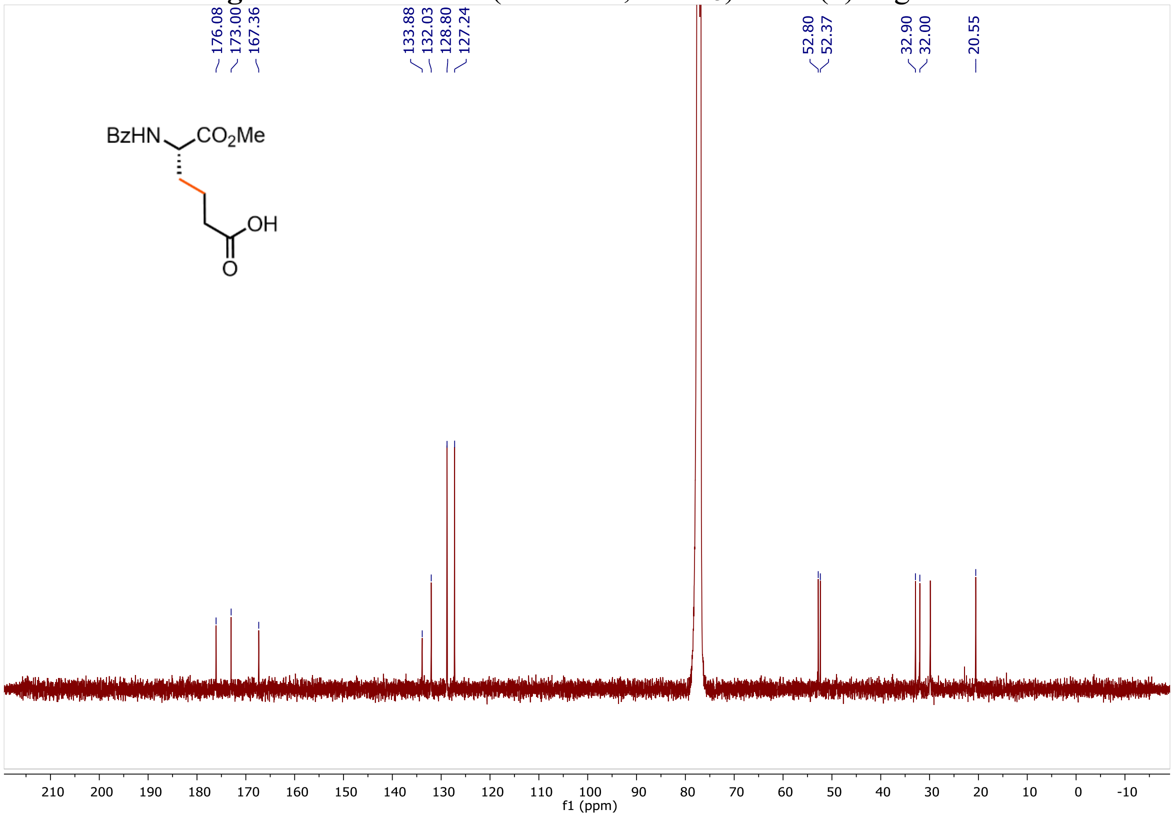

Figure S52. **^{13}C NMR** (101 MHz, $CDCl_3$) of **23**

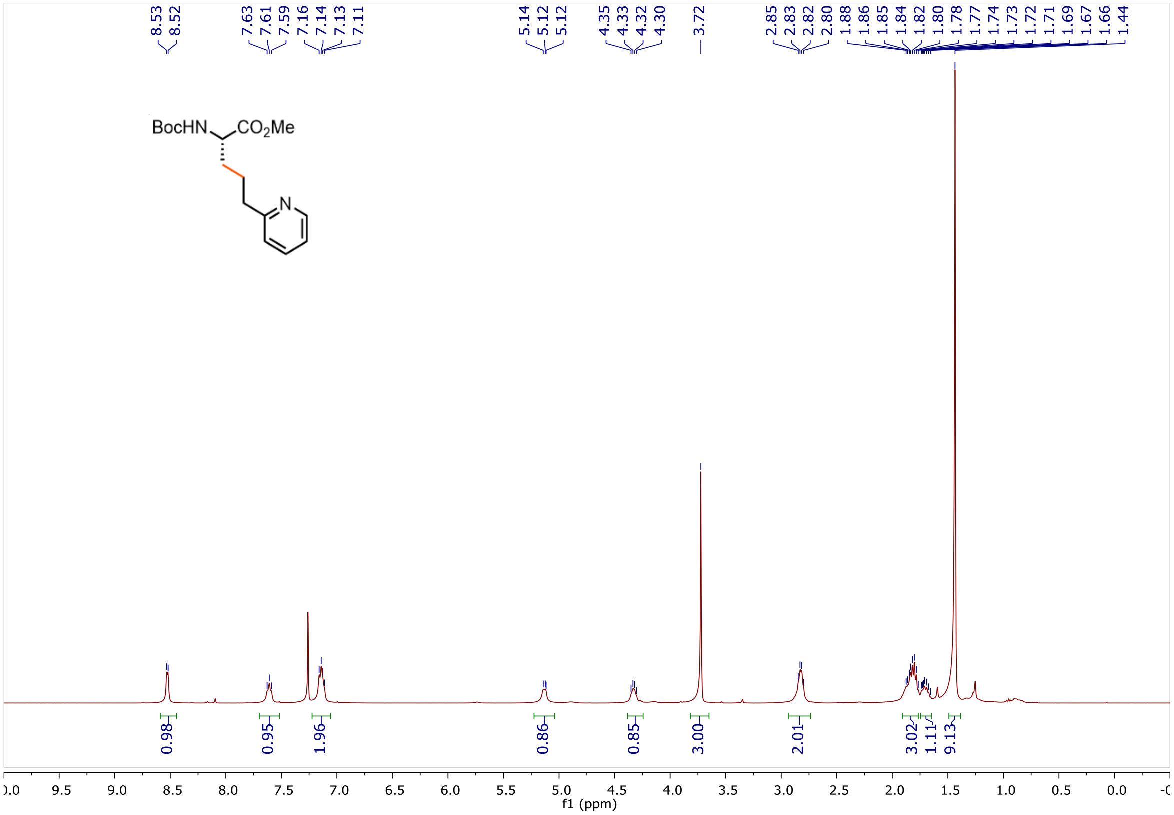

Figure S53. ^{1}**H NMR** (400 MHz, $CDCl_3$) of **24**

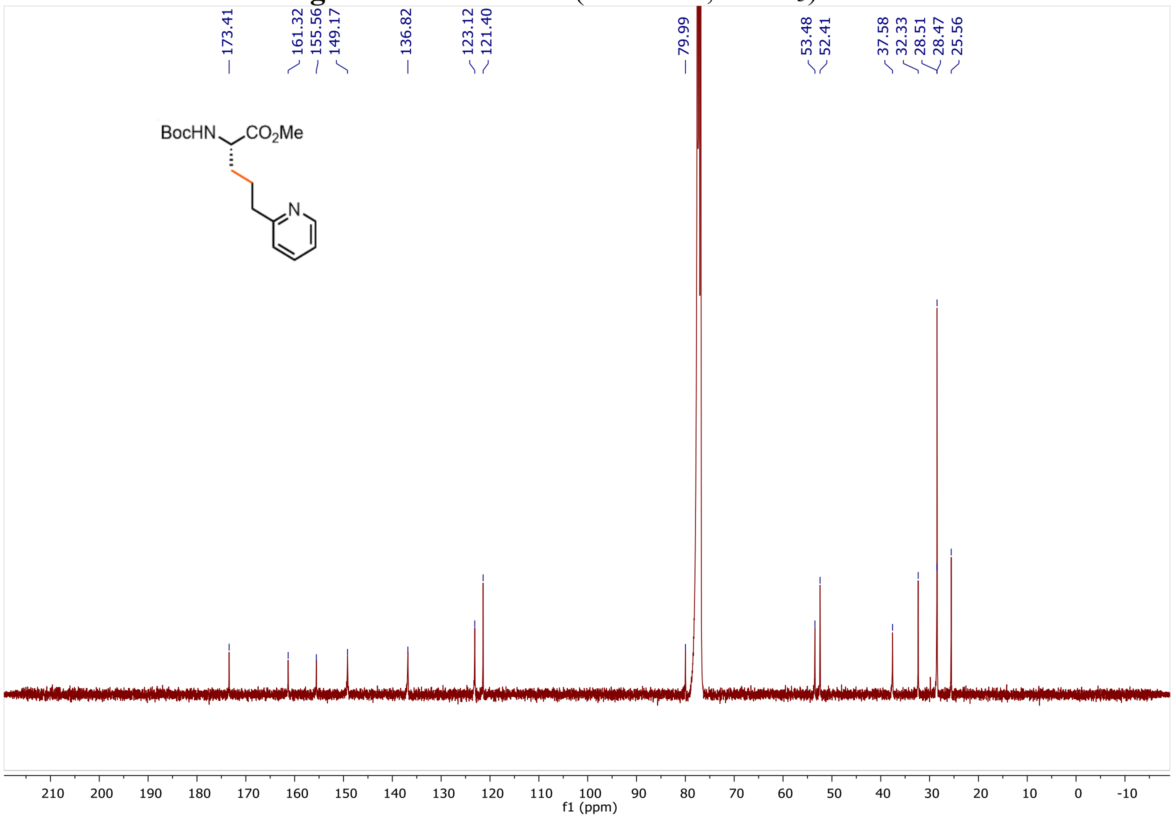

Figure S54. ^{13}**C NMR** (101 MHz, $CDCl_3$) of **24**

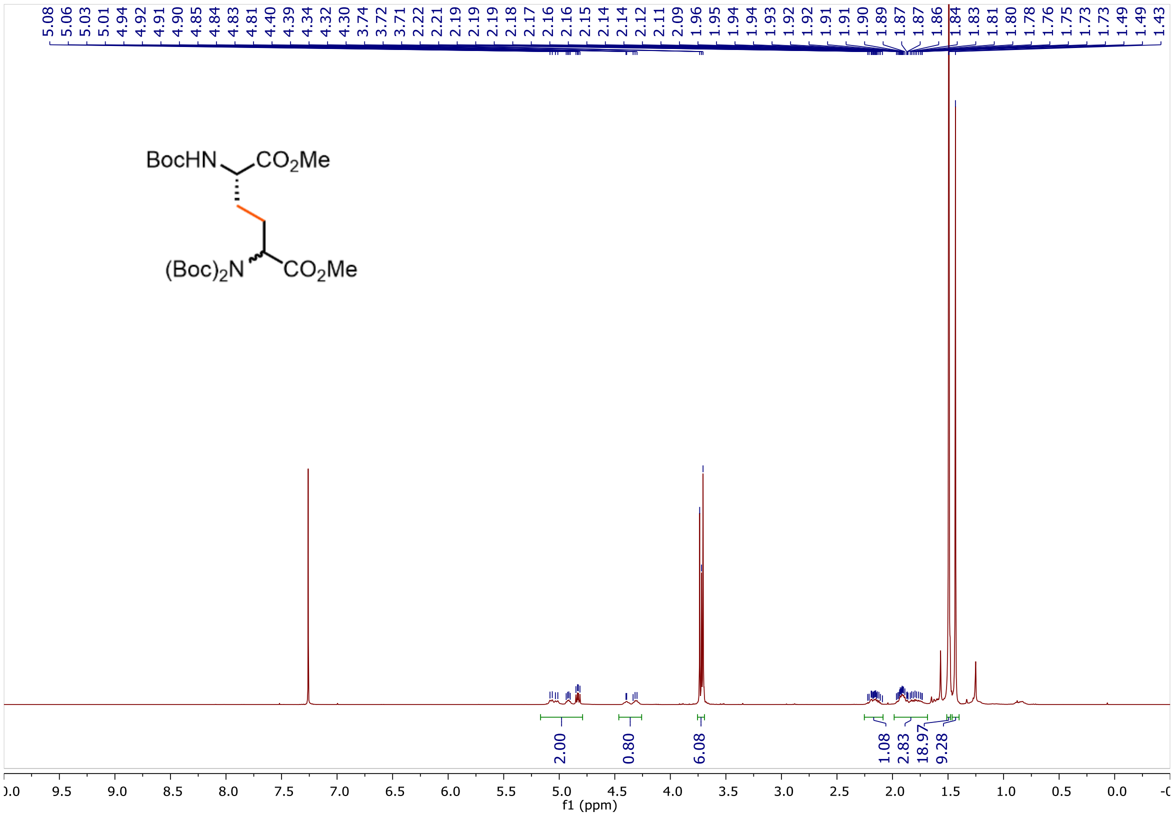

Figure S55. ^{1}H NMR (400 MHz, $CDCl_3$) of **25**

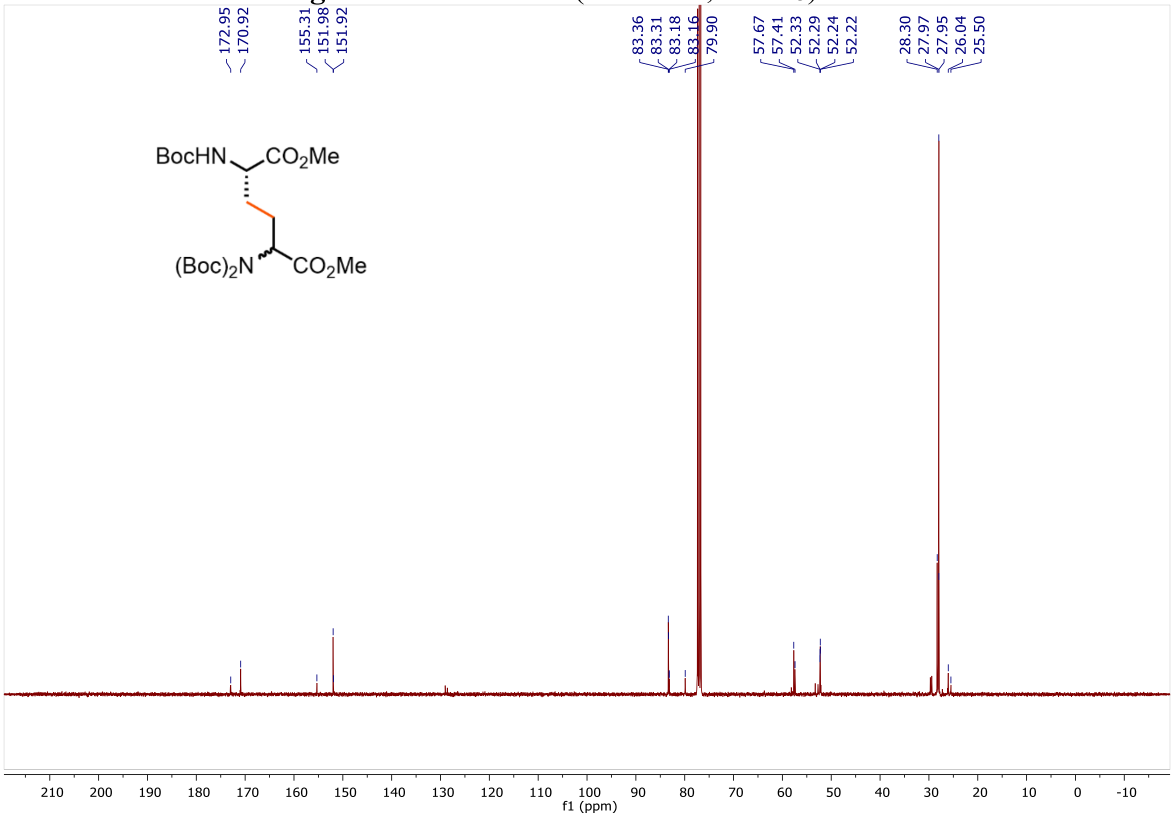

Figure S56. ^{13}C NMR (101 MHz, $CDCl_3$) of **25**

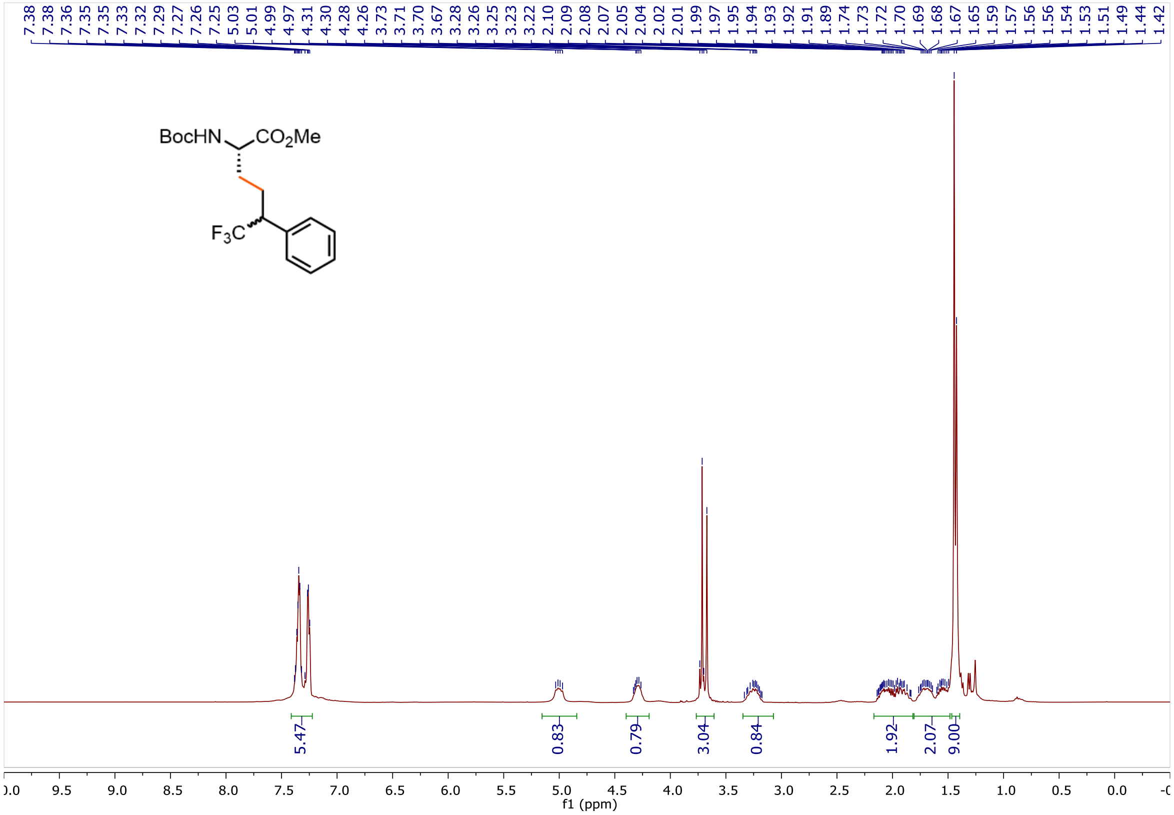

Figure S57. ^{1}**H NMR** (400 MHz, $CDCl_3$) of **26**

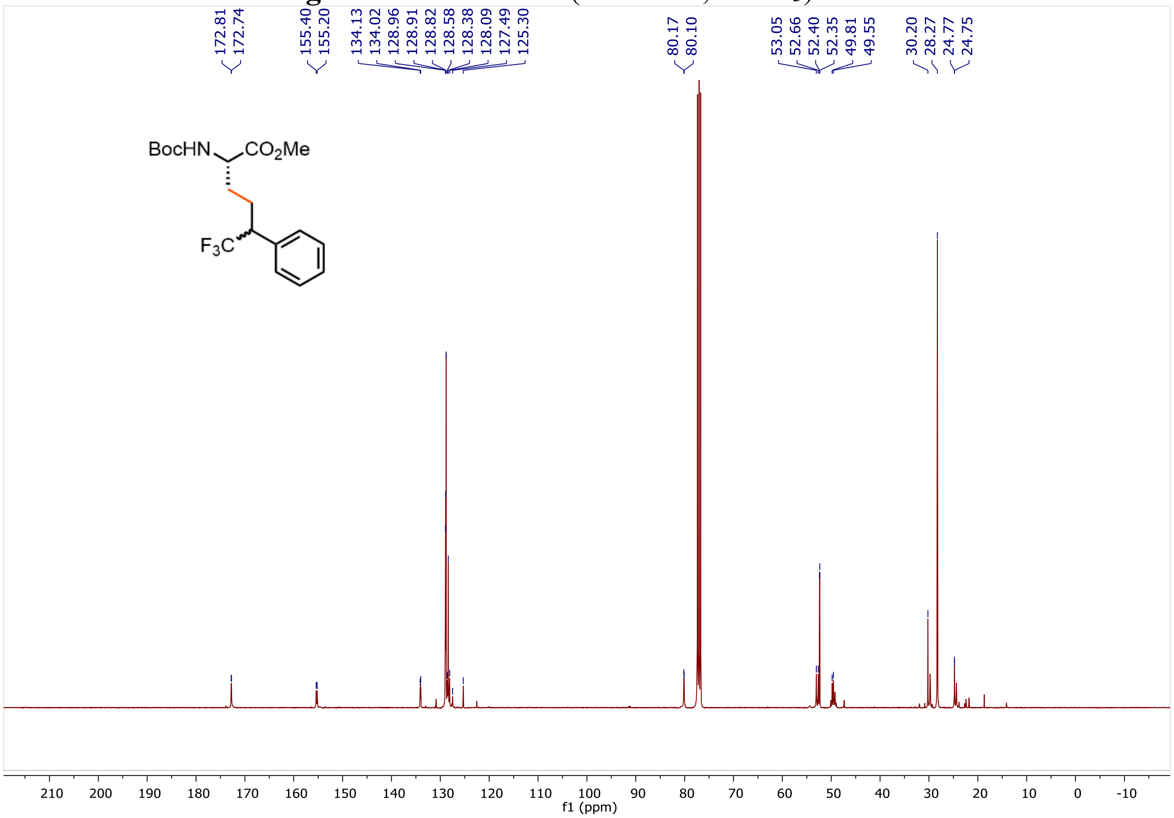

Figure S58. ^{13}**C NMR** (101 MHz, $CDCl_3$) of **26**

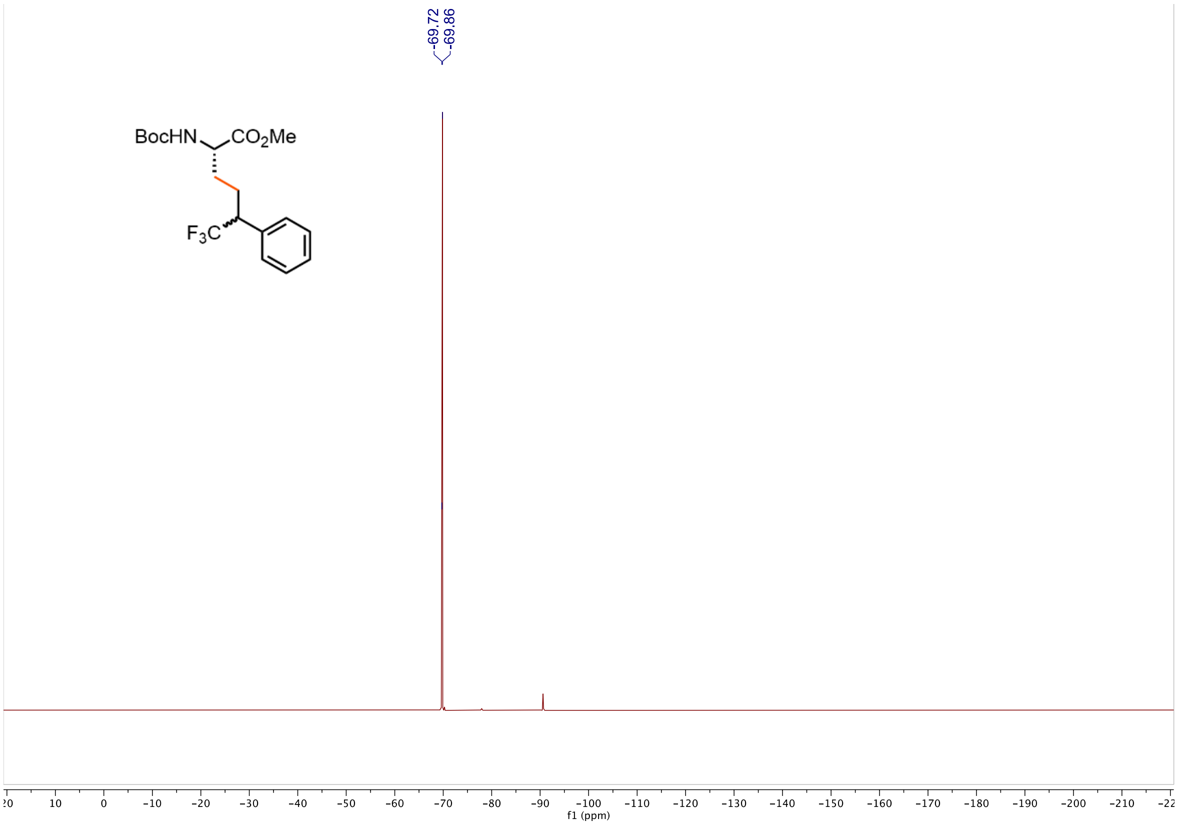

Figure S59. **^{19}F NMR** (471 MHz, $CDCl_3$) of **26**

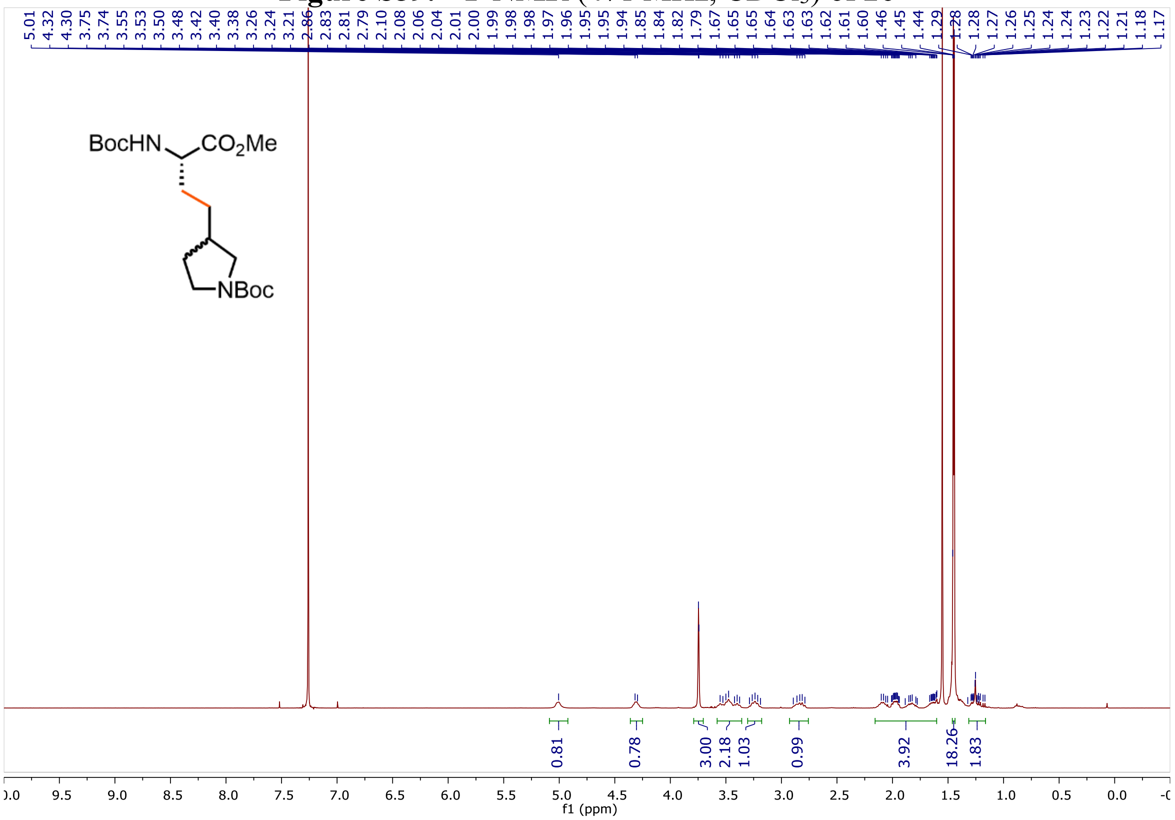

Figure S60. **^{1}H NMR** (400 MHz, $CDCl_3$) of **27**

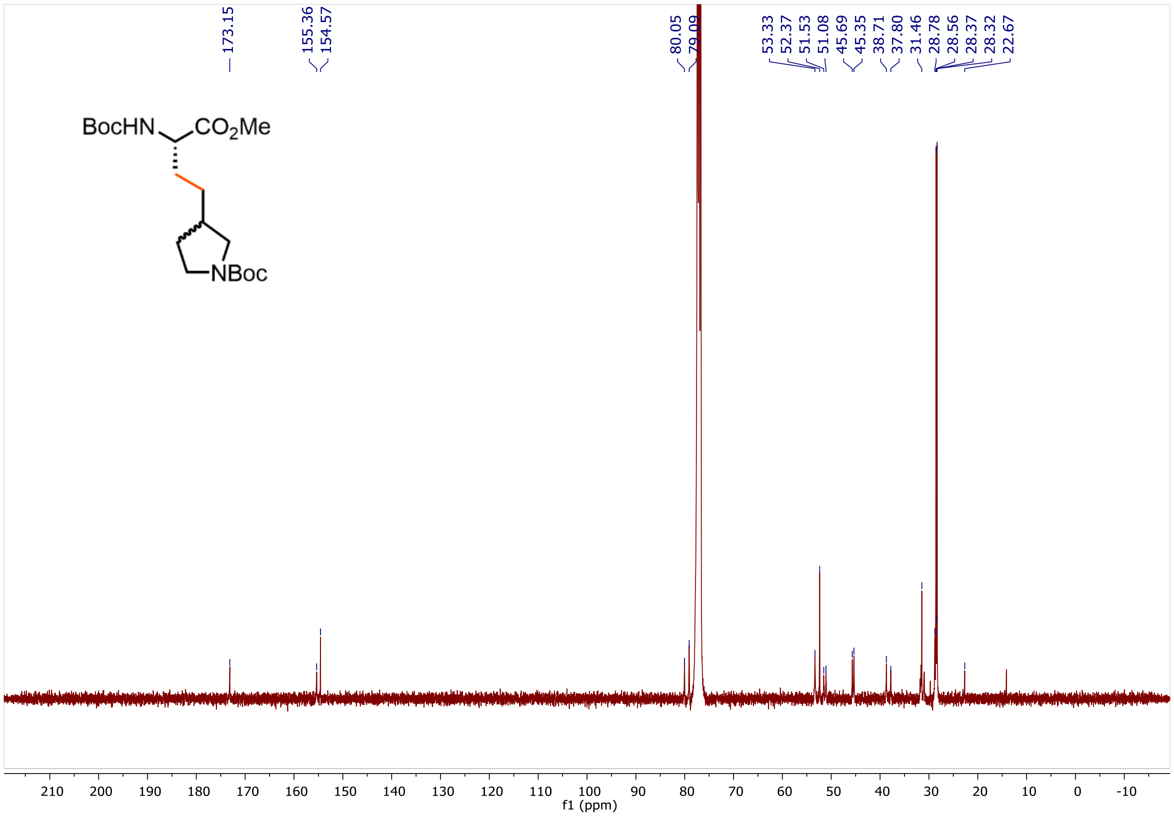

Figure S61. ^{13}**C NMR** (101 MHz, $CDCl_3$) of **27**

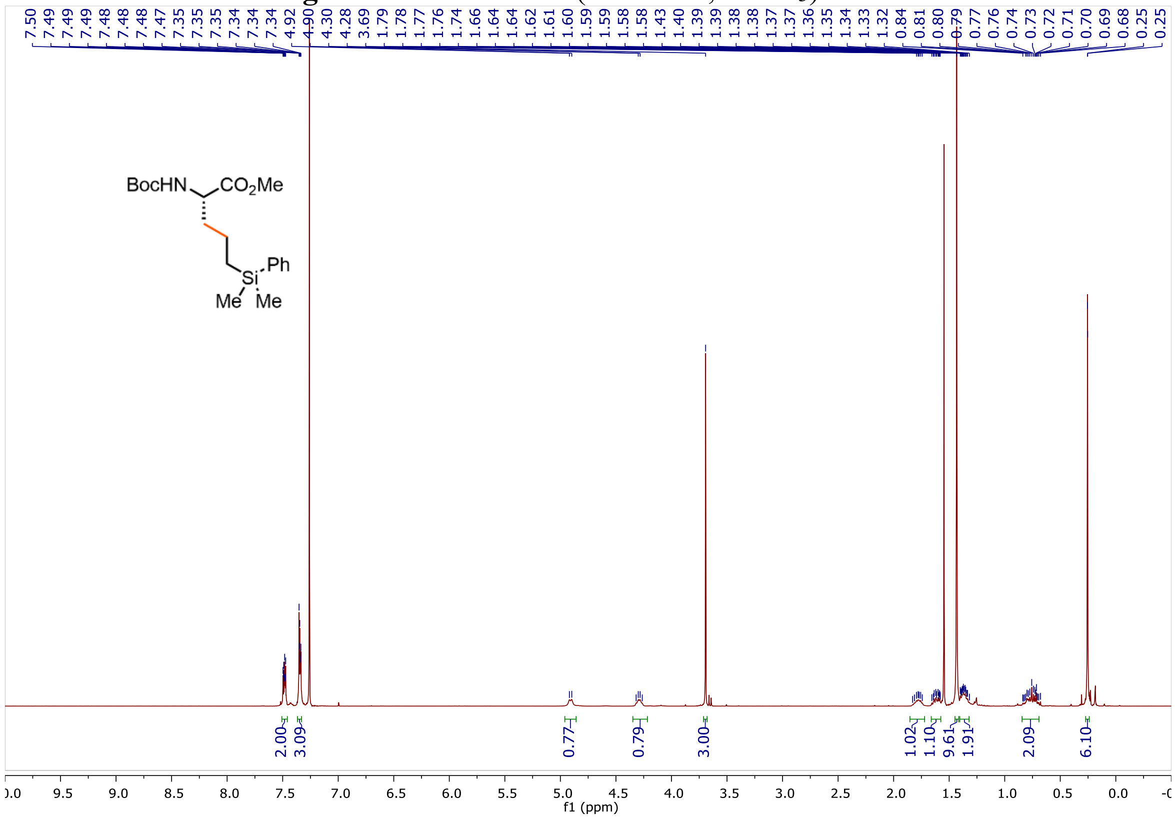

Figure S62. ^{1}**H NMR** (400 MHz, $CDCl_3$) of **28**

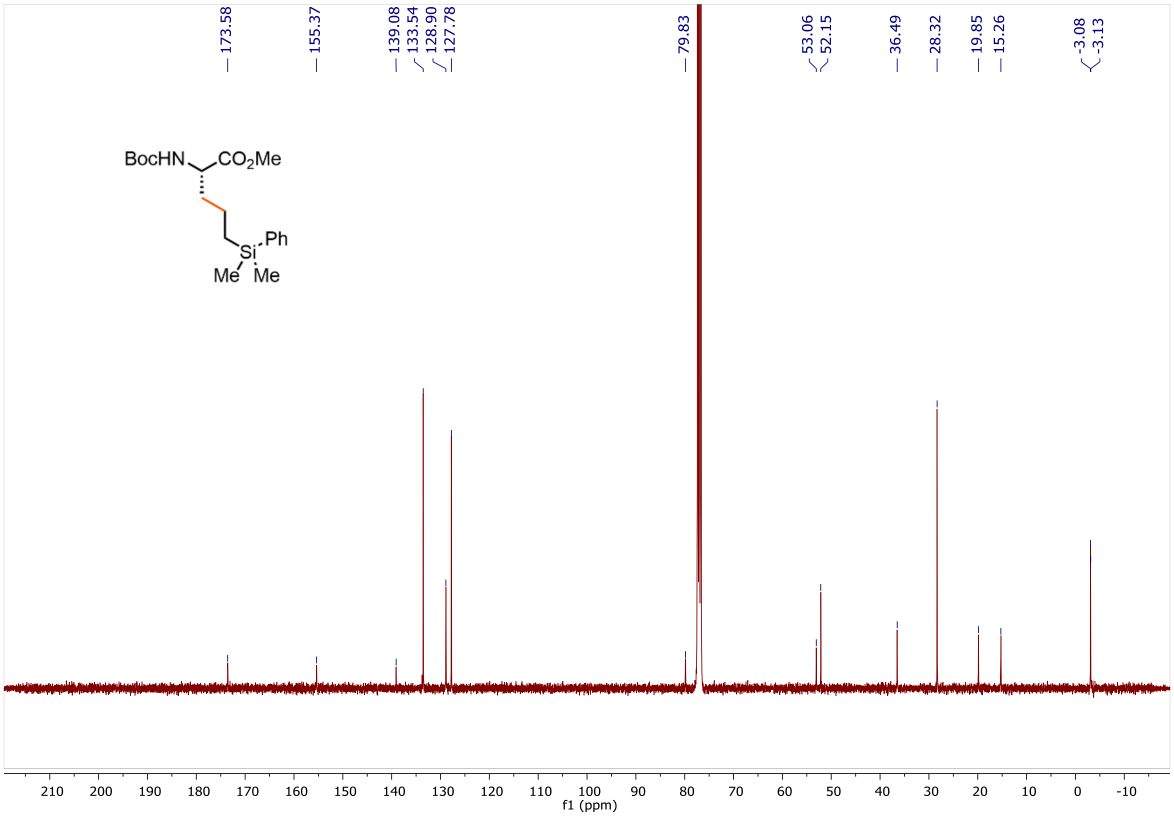

Figure S63. **^{13}C NMR** (101 MHz, $CDCl_3$) of **28**

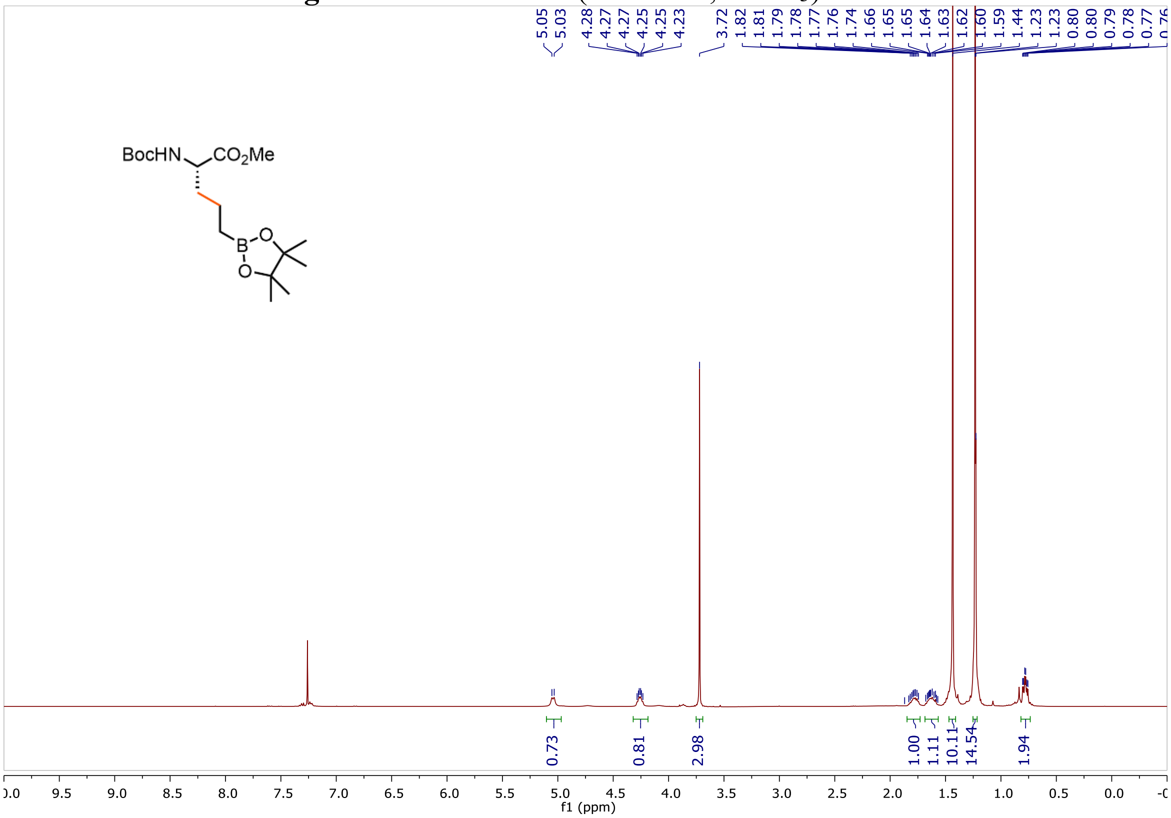

Figure S64. **^{1}H NMR** (400 MHz, $CDCl_3$) of **29**

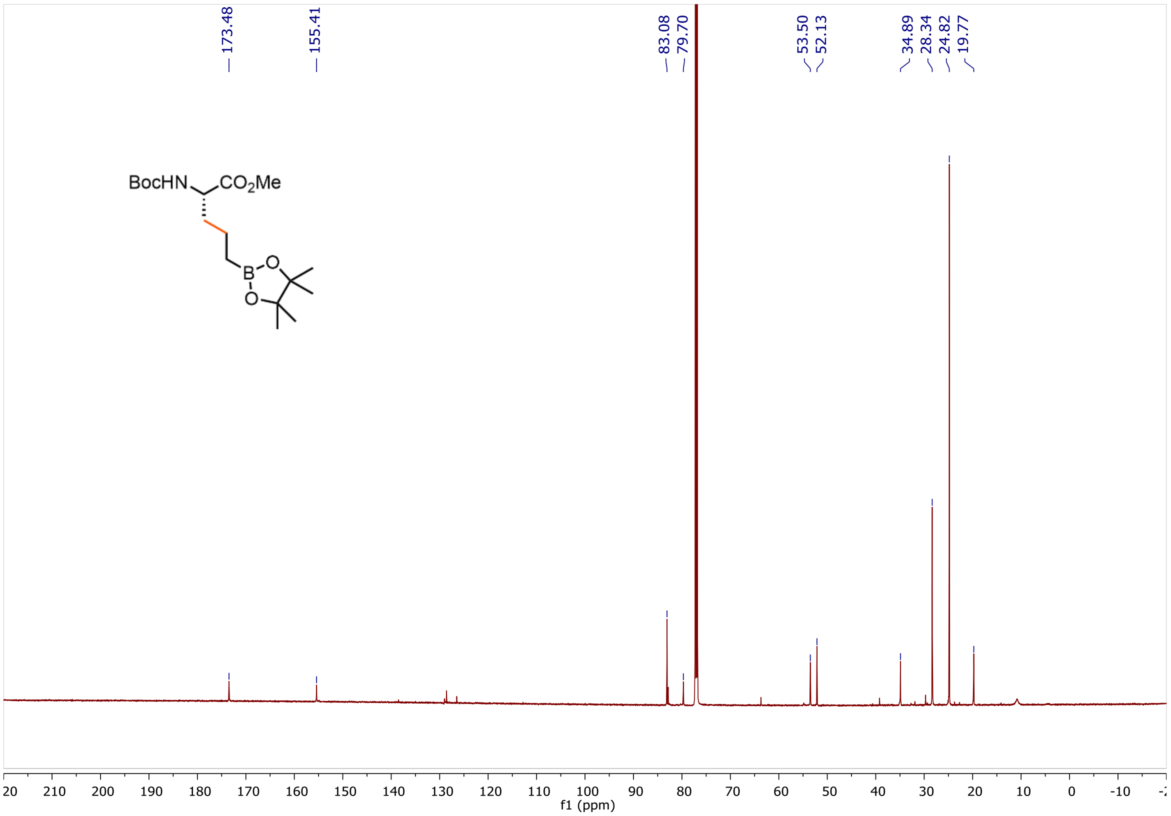

Figure S65. ^{13}**C NMR** (101 MHz, $CDCl_3$) of **29**

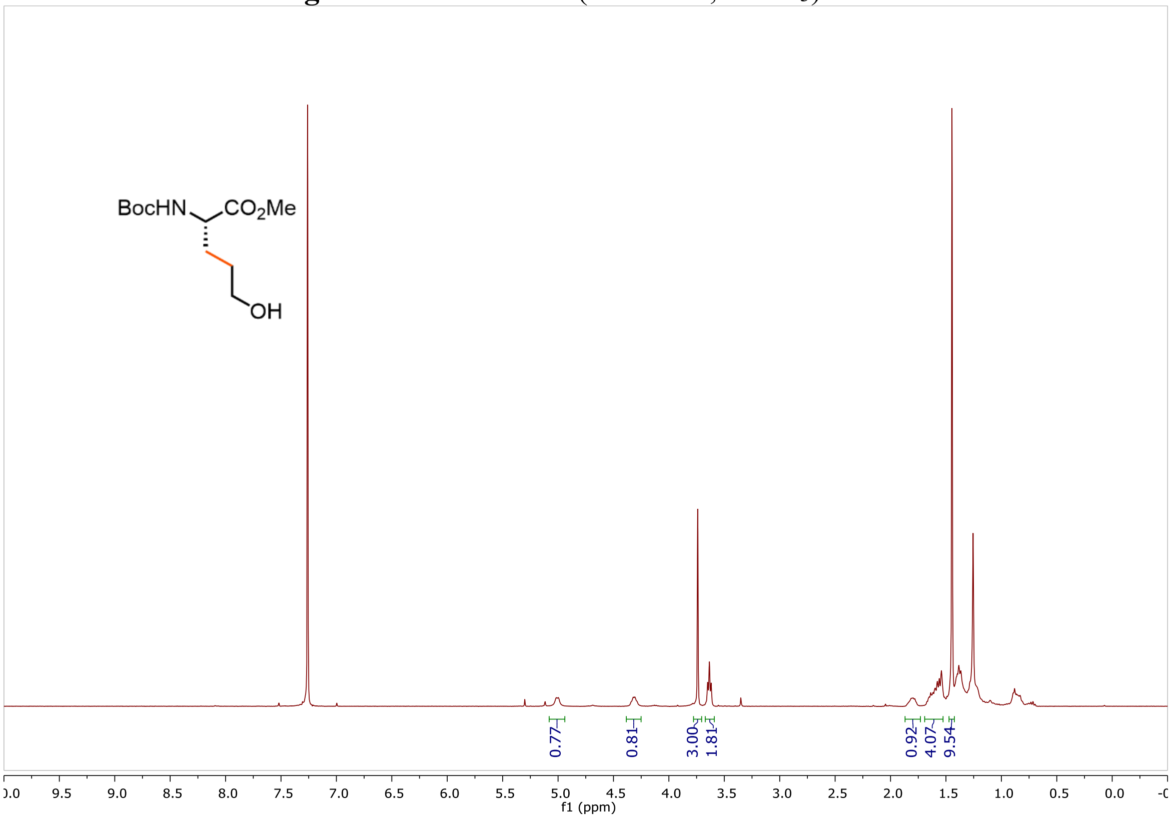

Figure S66. ^{1}**H NMR** (400 MHz, $CDCl_3$) of **30**

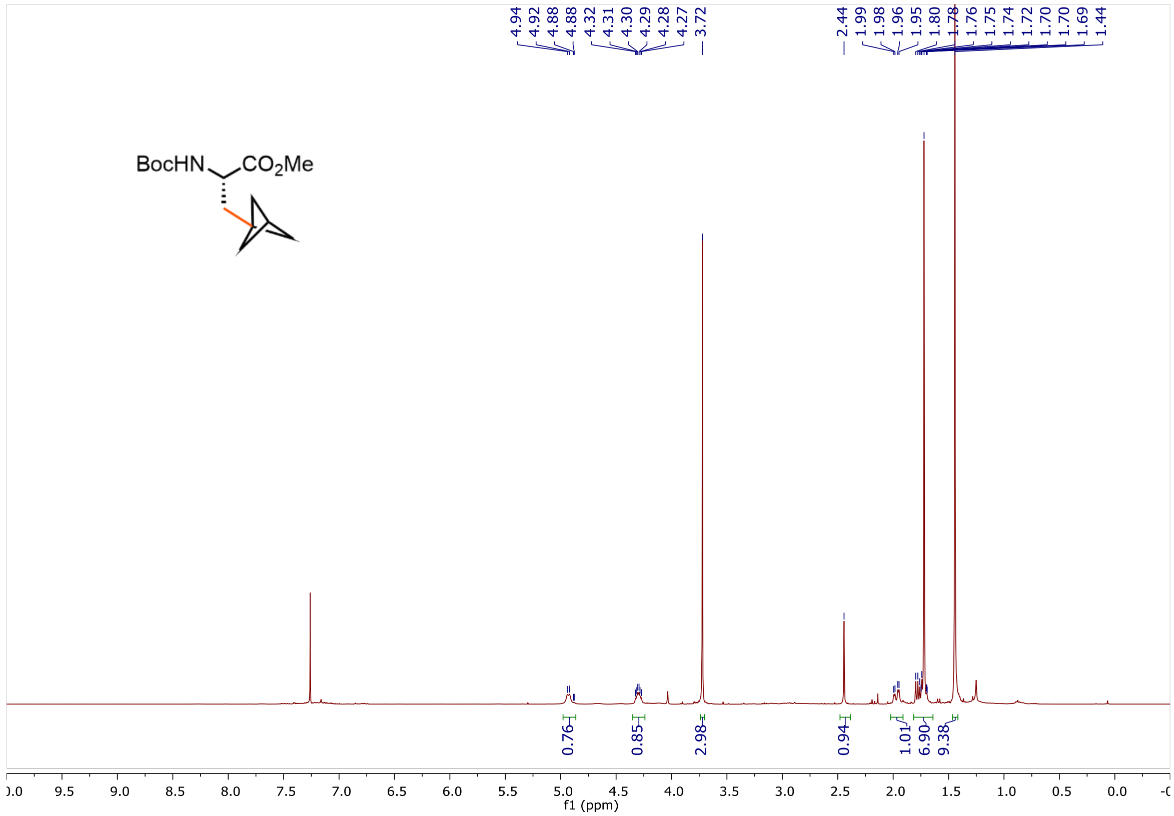

Figure S67. ^{1}**H NMR** (400 MHz, $CDCl_3$) of **31**

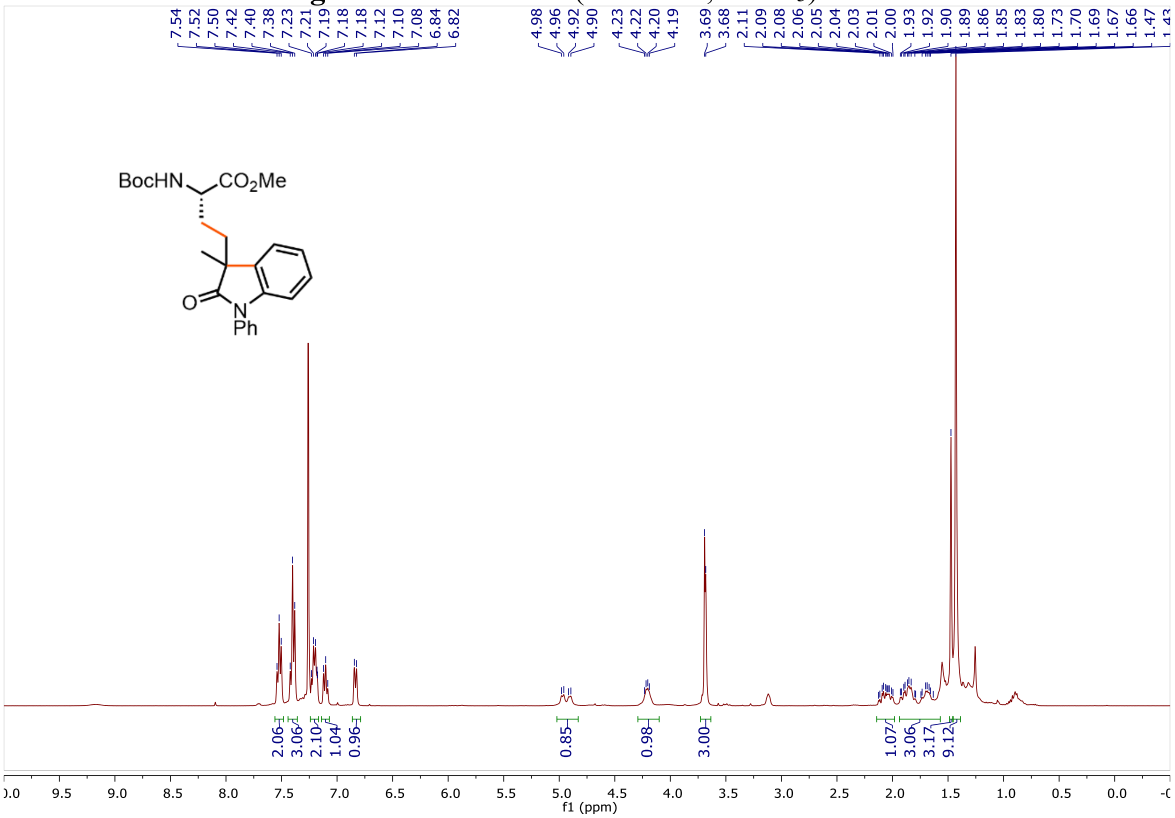

Figure S68. ^{1}**H NMR** (400 MHz, $CDCl_3$) of **32**

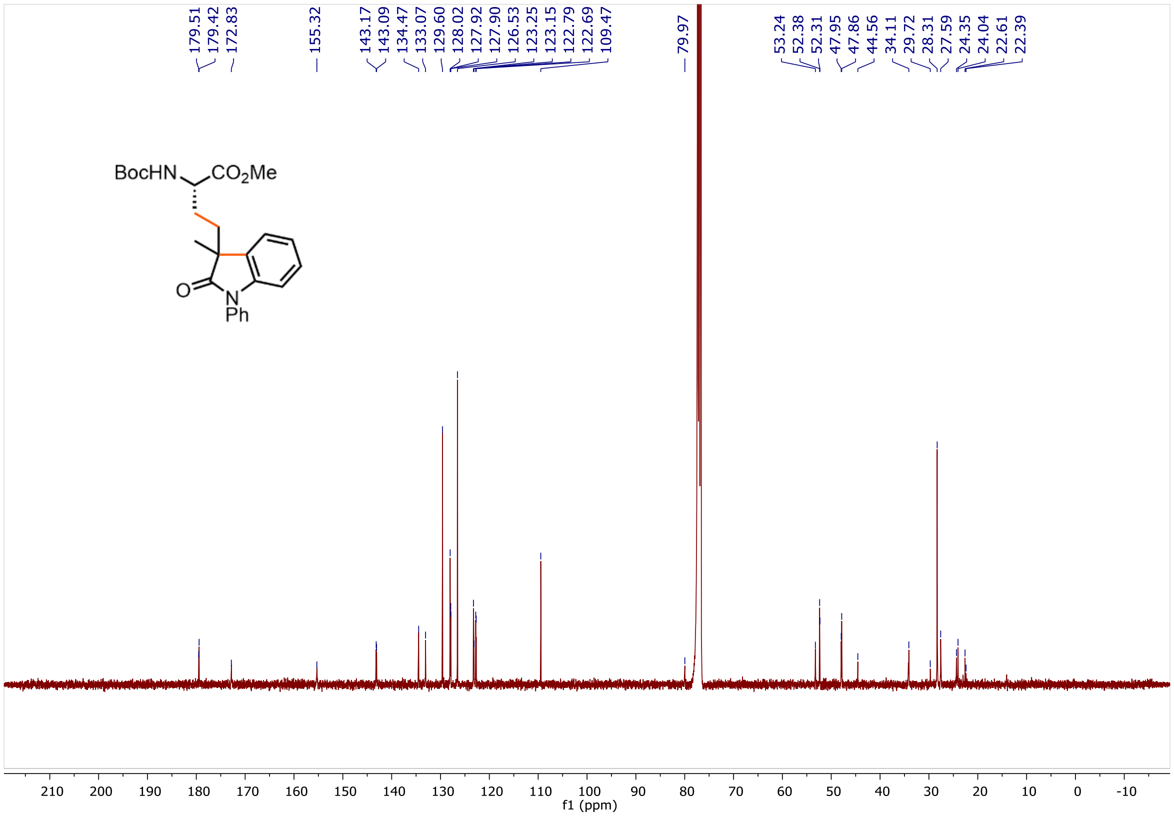

Figure S69. ^{13}C NMR (101 MHz, $CDCl_3$) of **32**

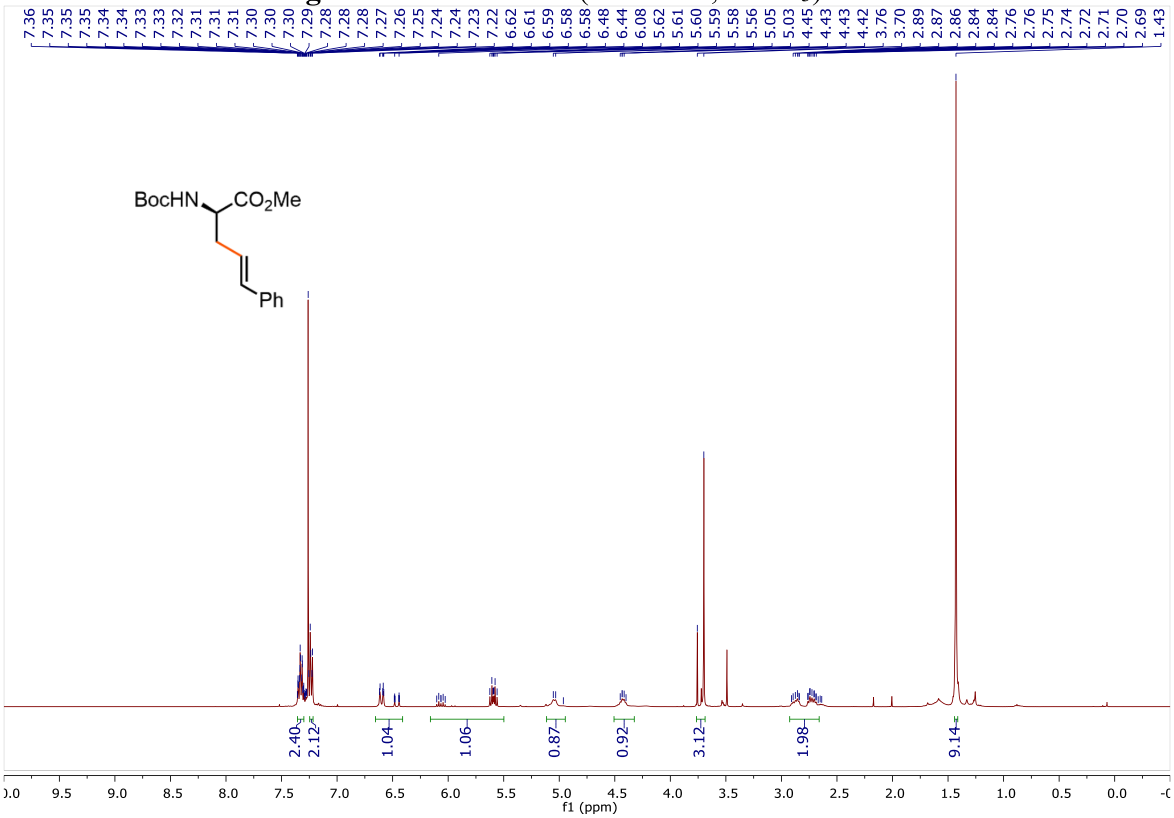

Figure S70. ^{1}H NMR (400 MHz, $CDCl_3$) of **33**

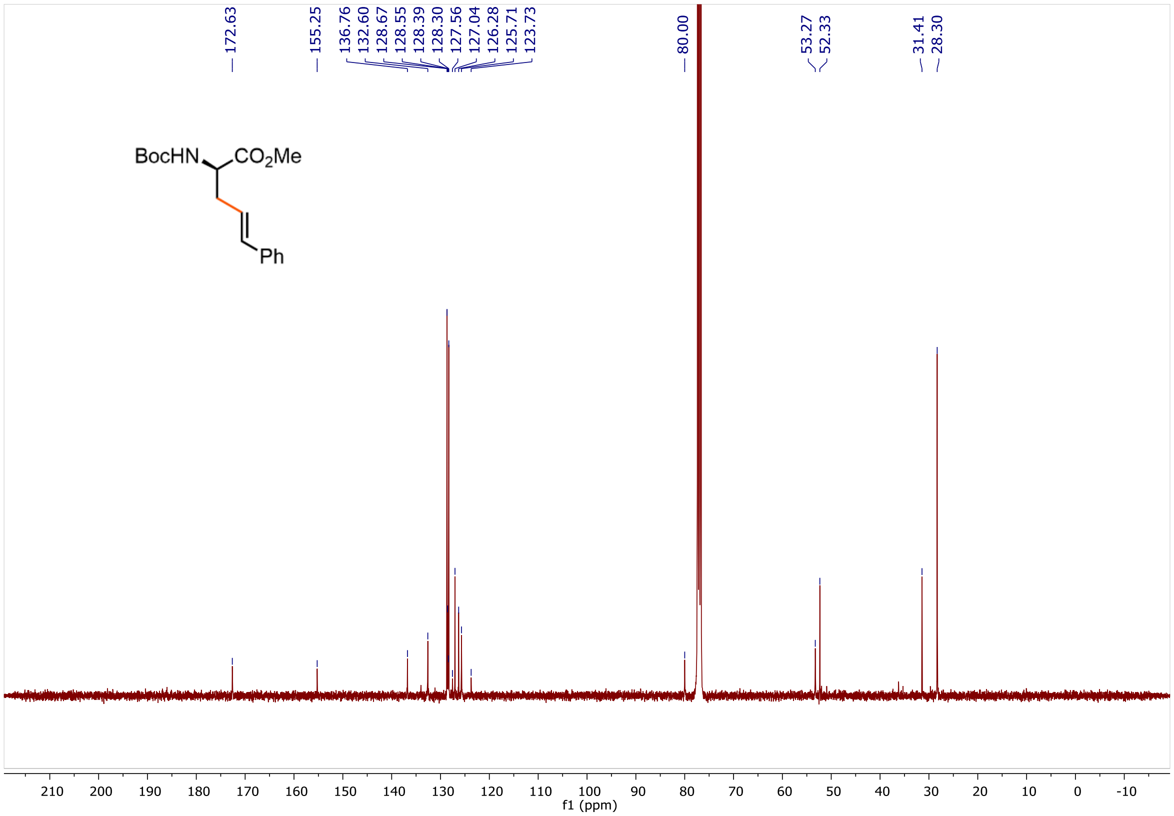

Figure S71. ^{13}**C NMR** (101 MHz, $CDCl_3$) of **33**

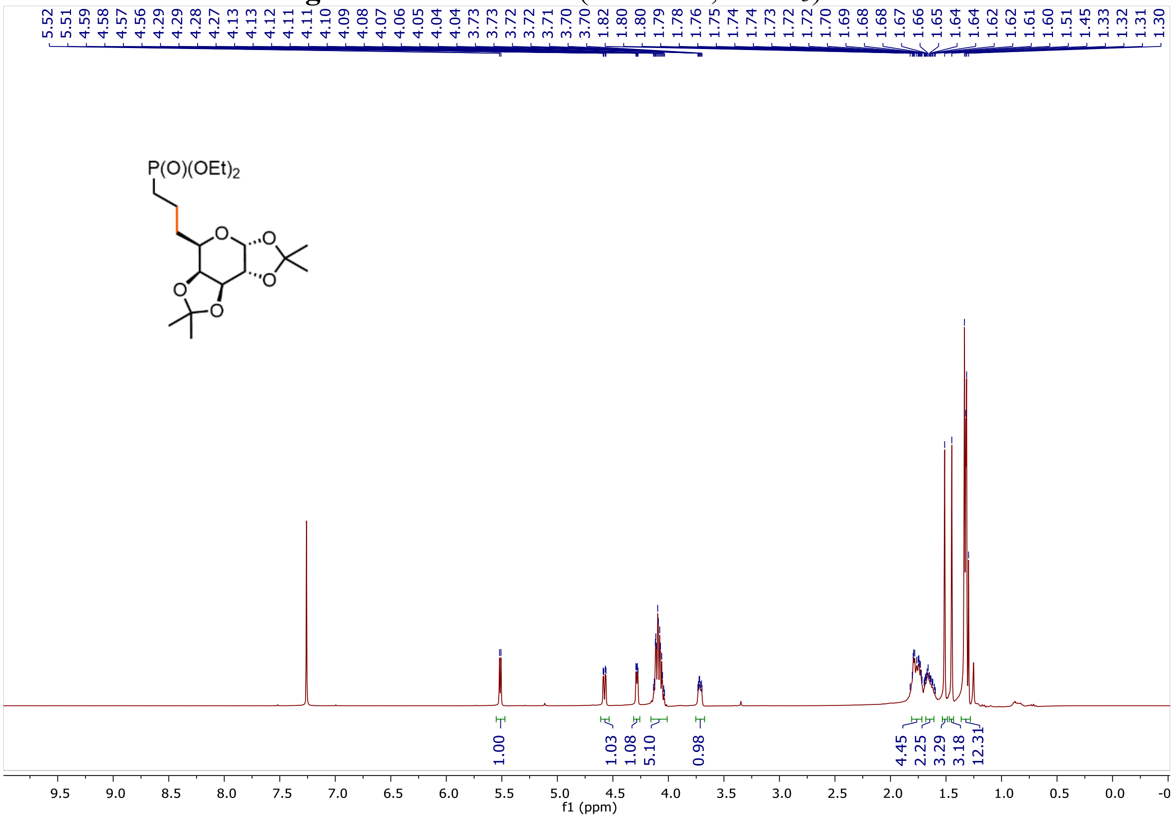

Figure S72. ^{1}**H NMR** (400 MHz, $CDCl_3$) of **34**

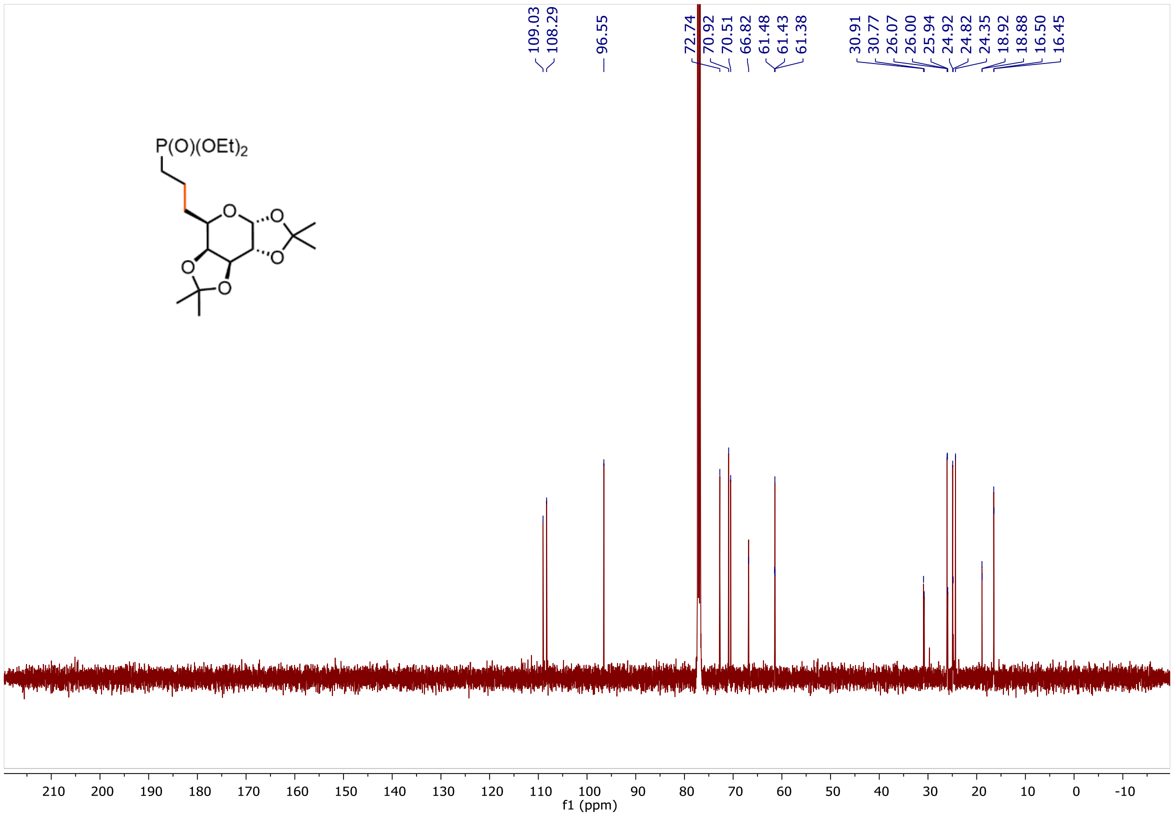

Figure S73. ^{13}**C NMR** (101 MHz, $CDCl_3$) of **34**

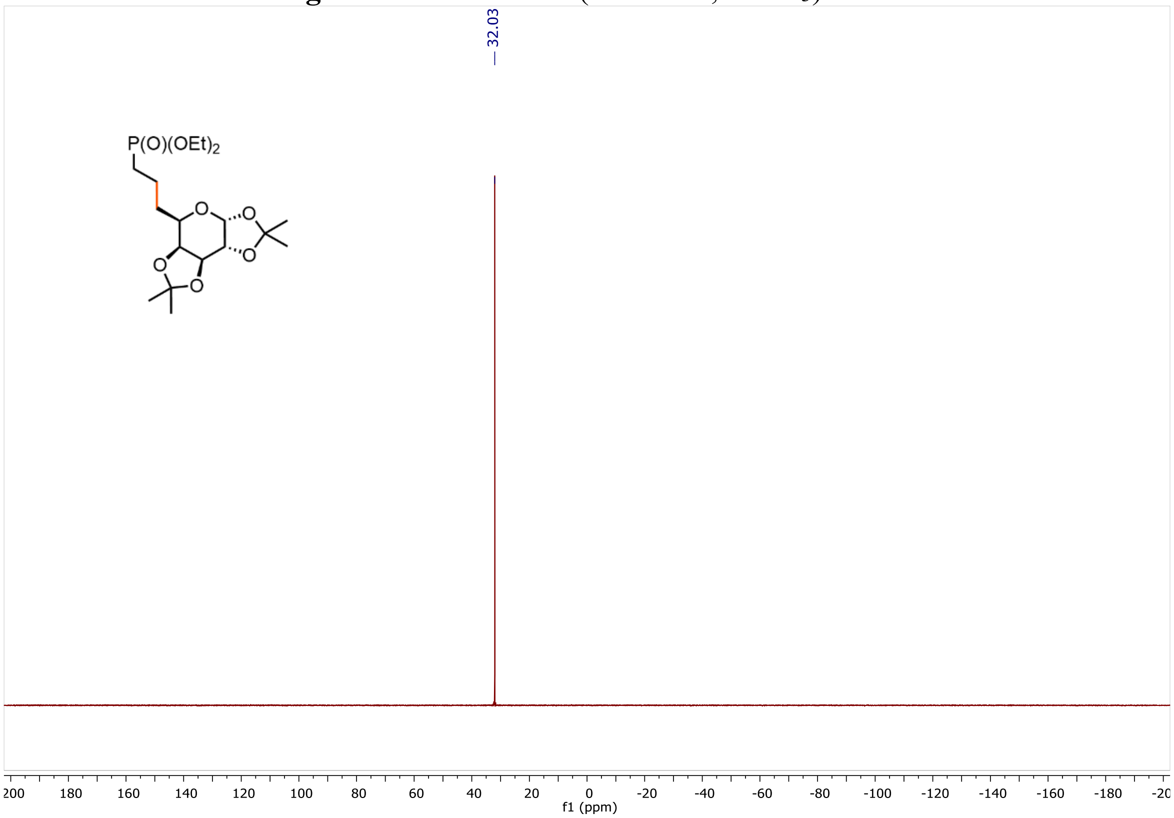

Figure S74. ^{31}**P NMR** (202 MHz, $CDCl_3$) of **34**

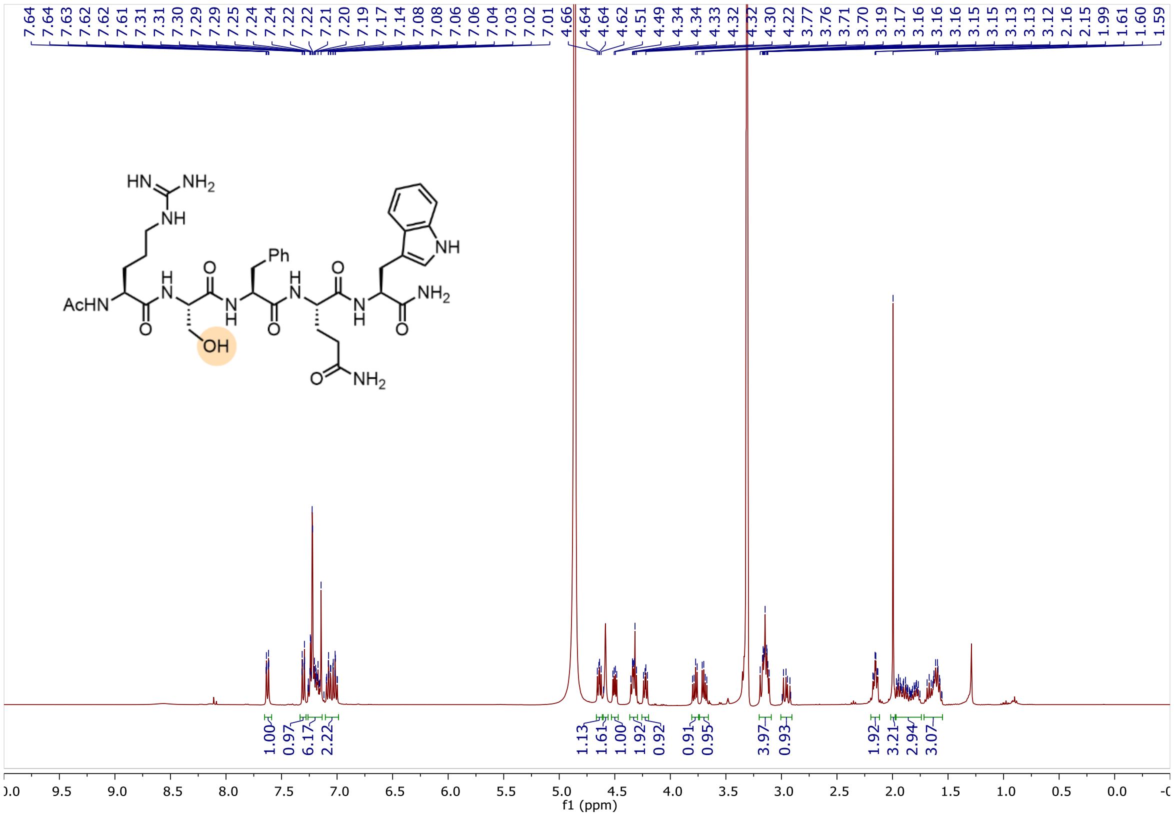

Figure S75. ¹H NMR (400 MHz, MeOD) of **39**

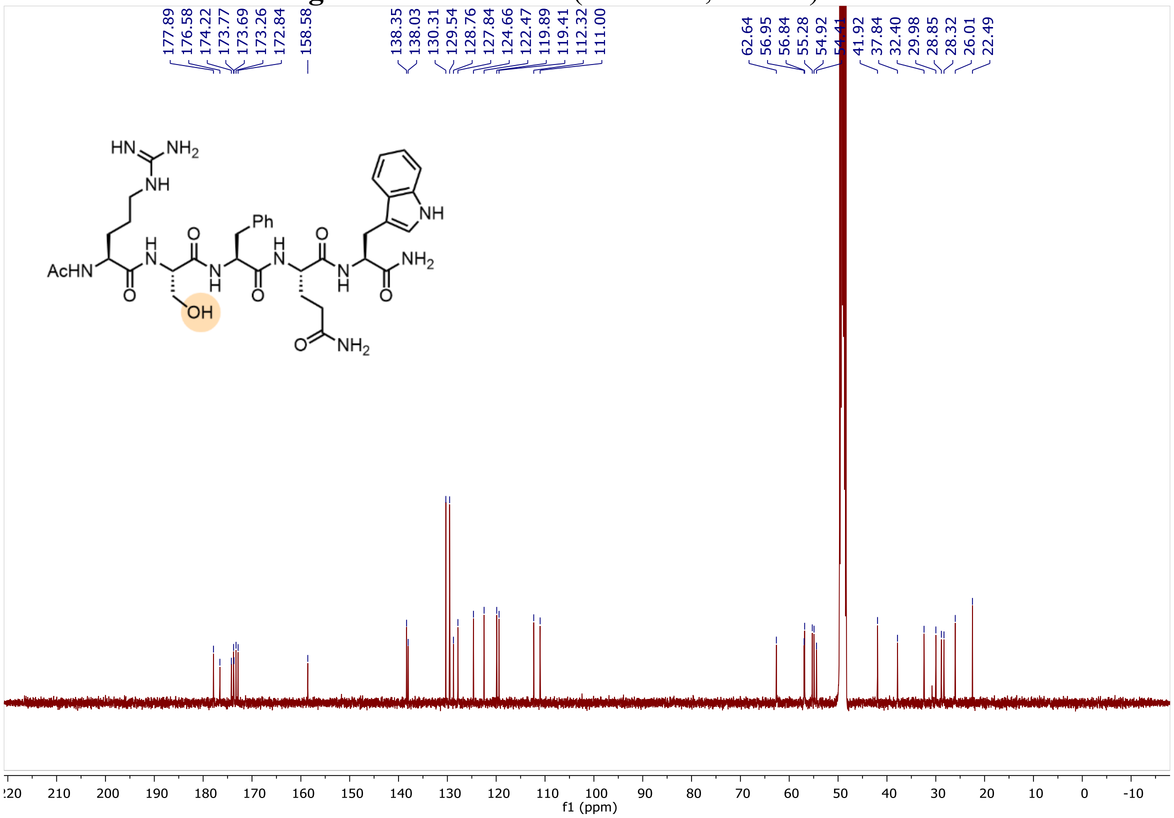

Figure S76. ¹³C NMR (101 MHz, MeOD) of **39**

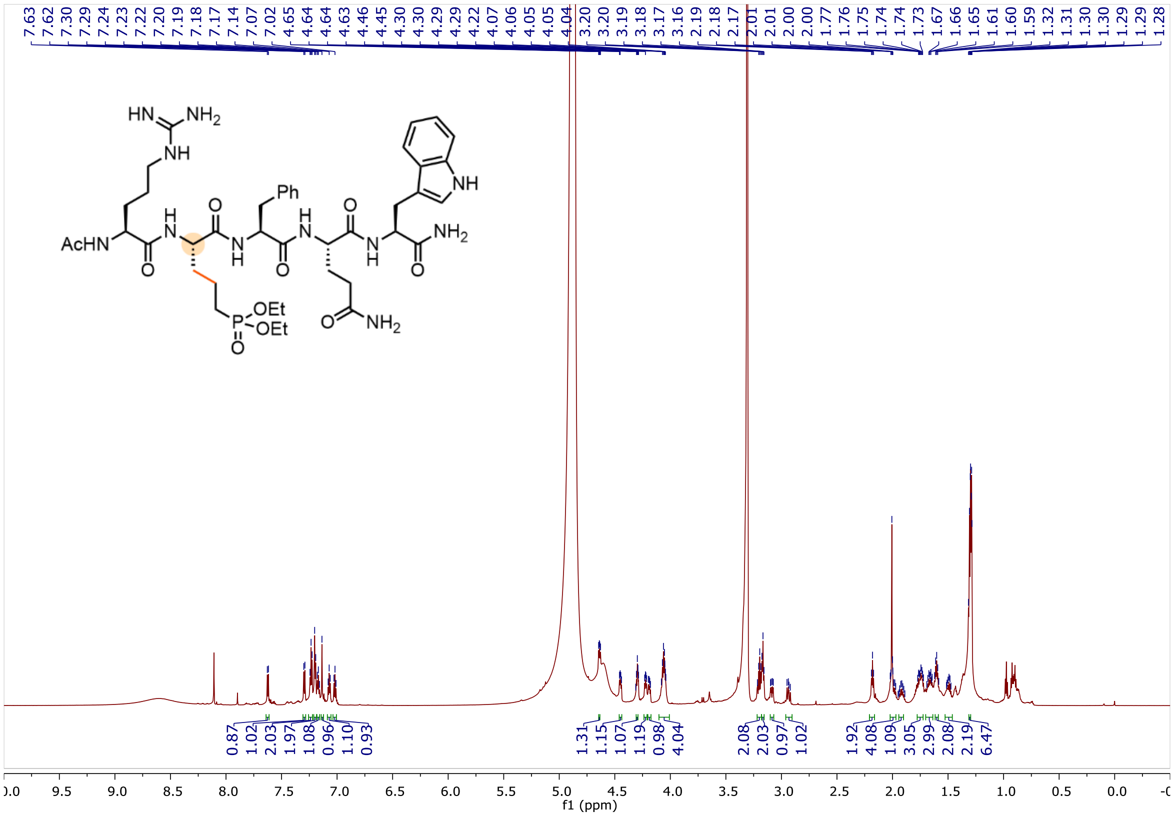

Figure S77. ^{1}H NMR (400 MHz, MeOD) of **37**

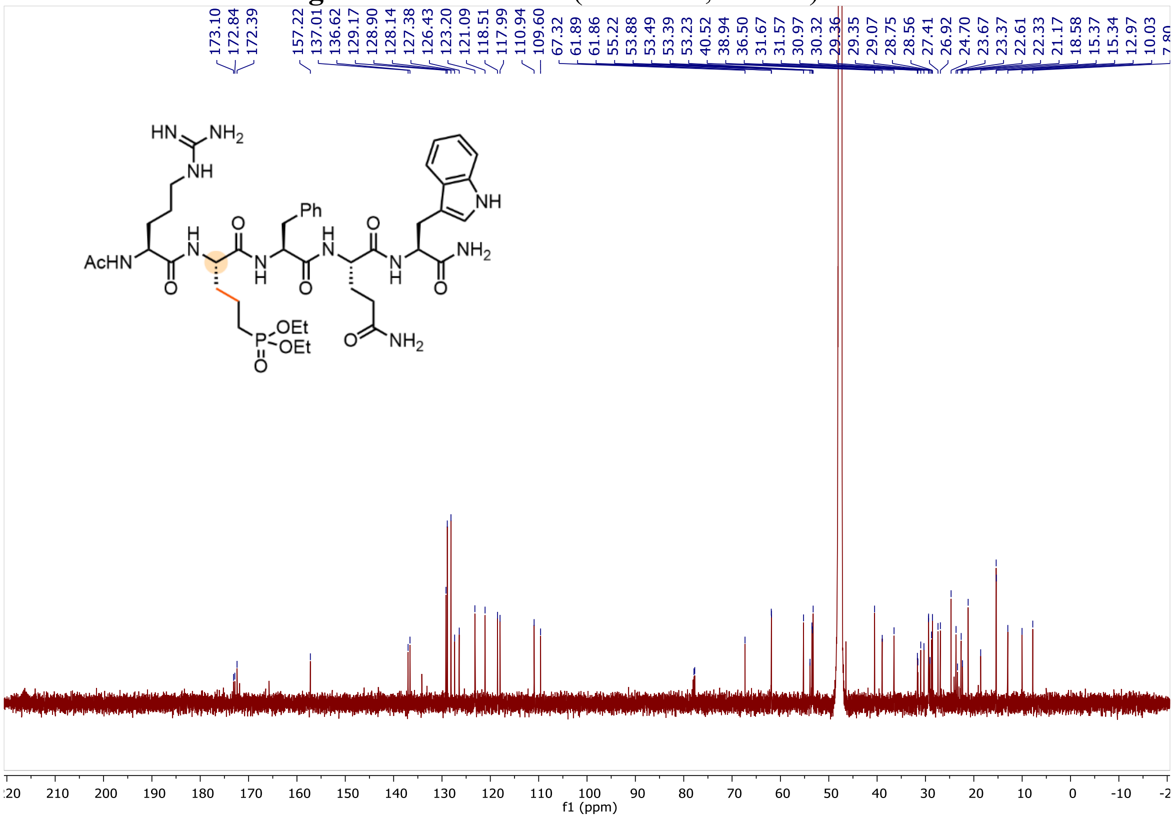

Figure S78. ^{13}C NMR (101 MHz, MeOD) of **37**

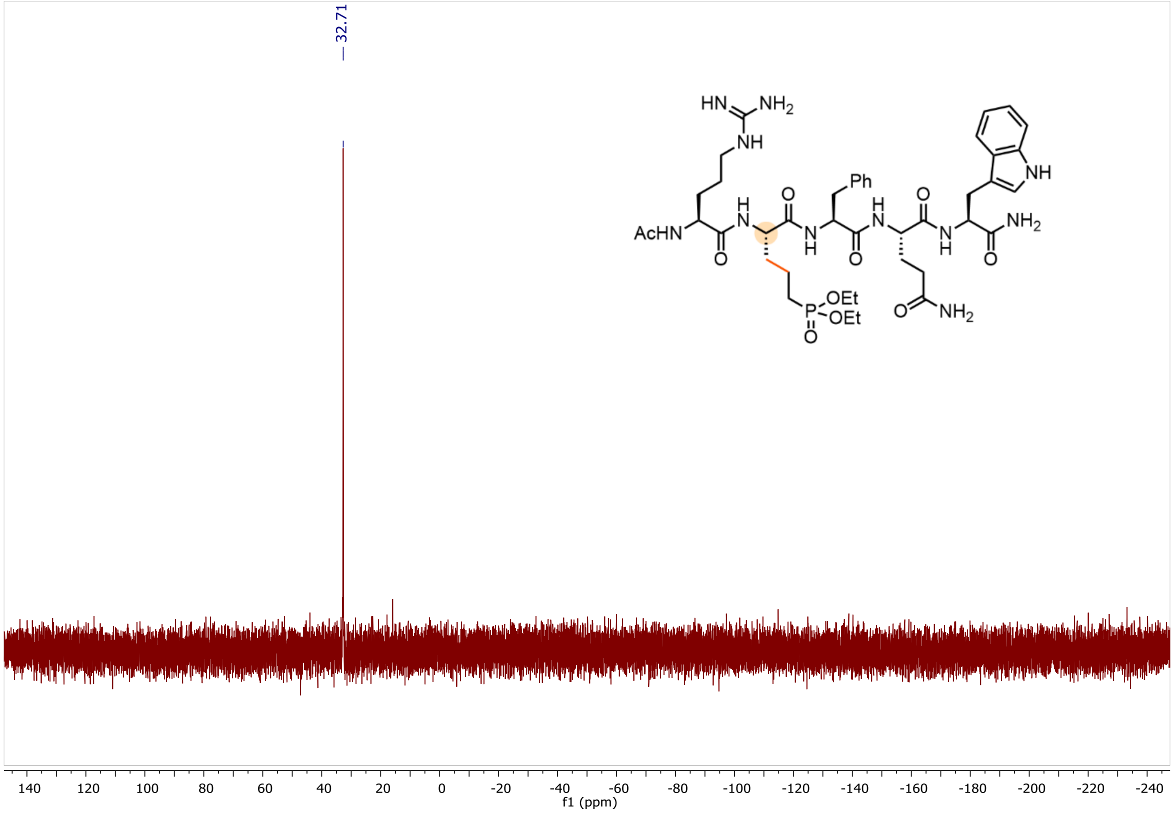

Figure S79. ^{31}**P NMR** (162 MHz, MeOD) of **37**

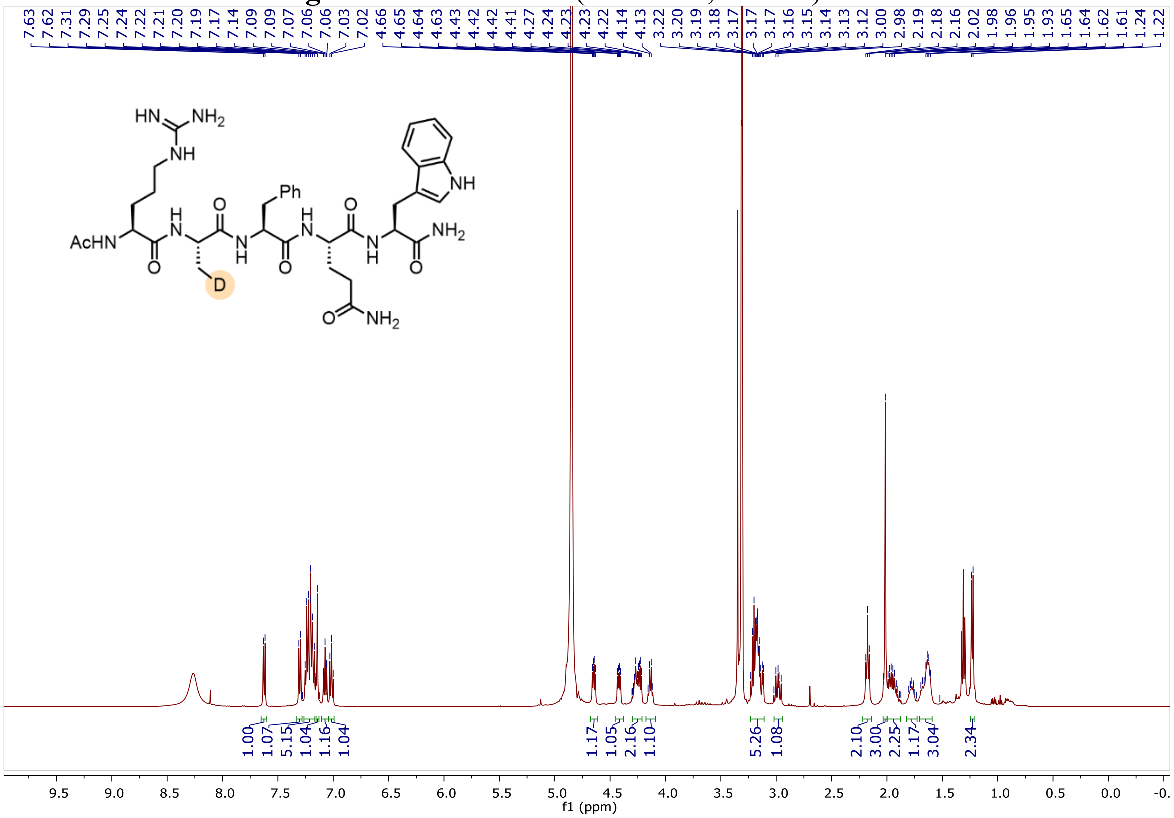

Figure S80. ^{1}**H NMR** (400 MHz, MeOD) of **38**

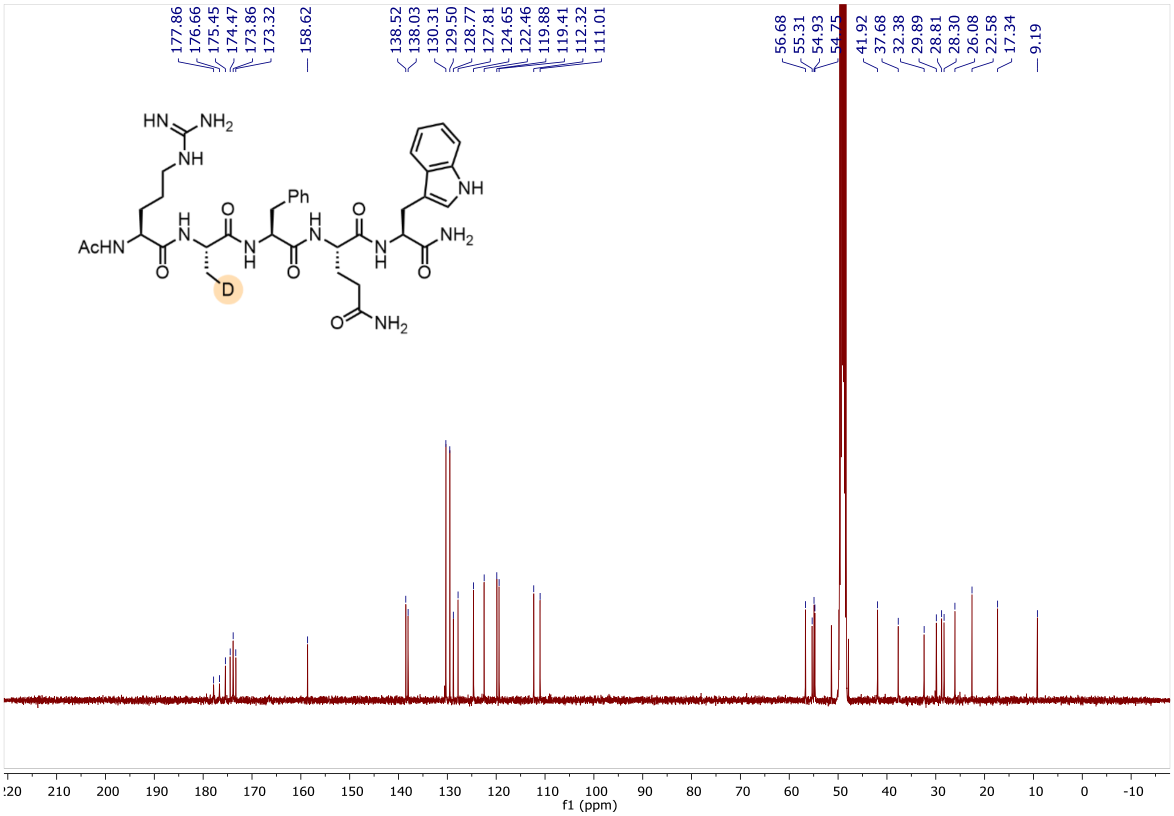

Figure S81. ^{13}**C NMR** (101 MHz, MeOD) of **38**

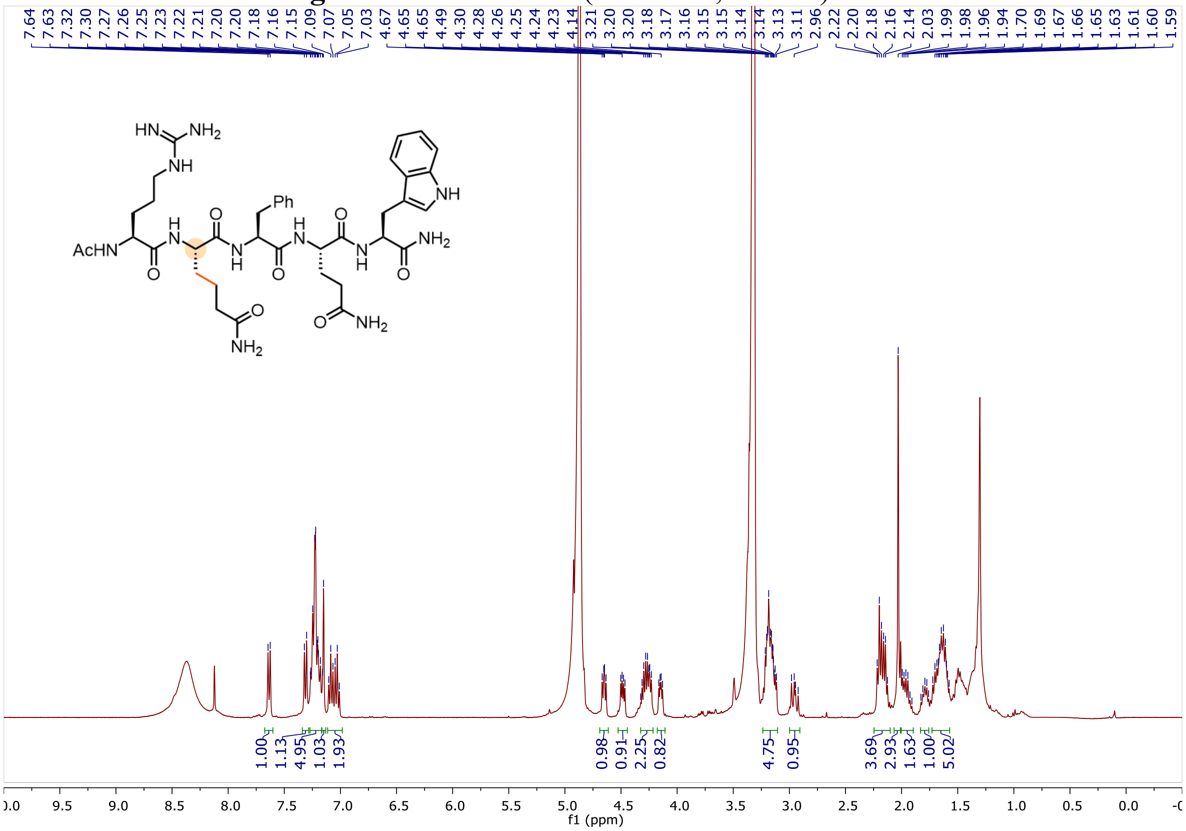

Figure S82. ^{1}**H NMR** (400 MHz, MeOD) of **40**

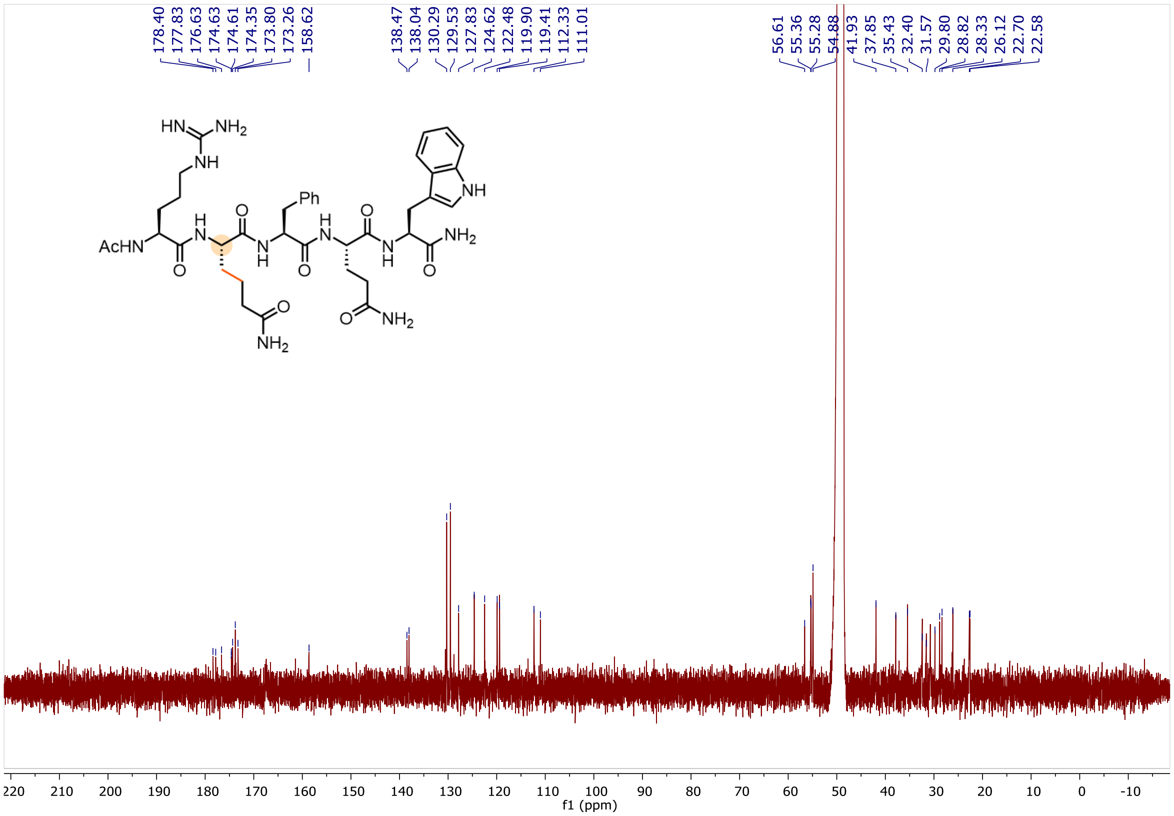

Figure S83. ^{13}**C NMR** (101 MHz, MeOD) of **40**

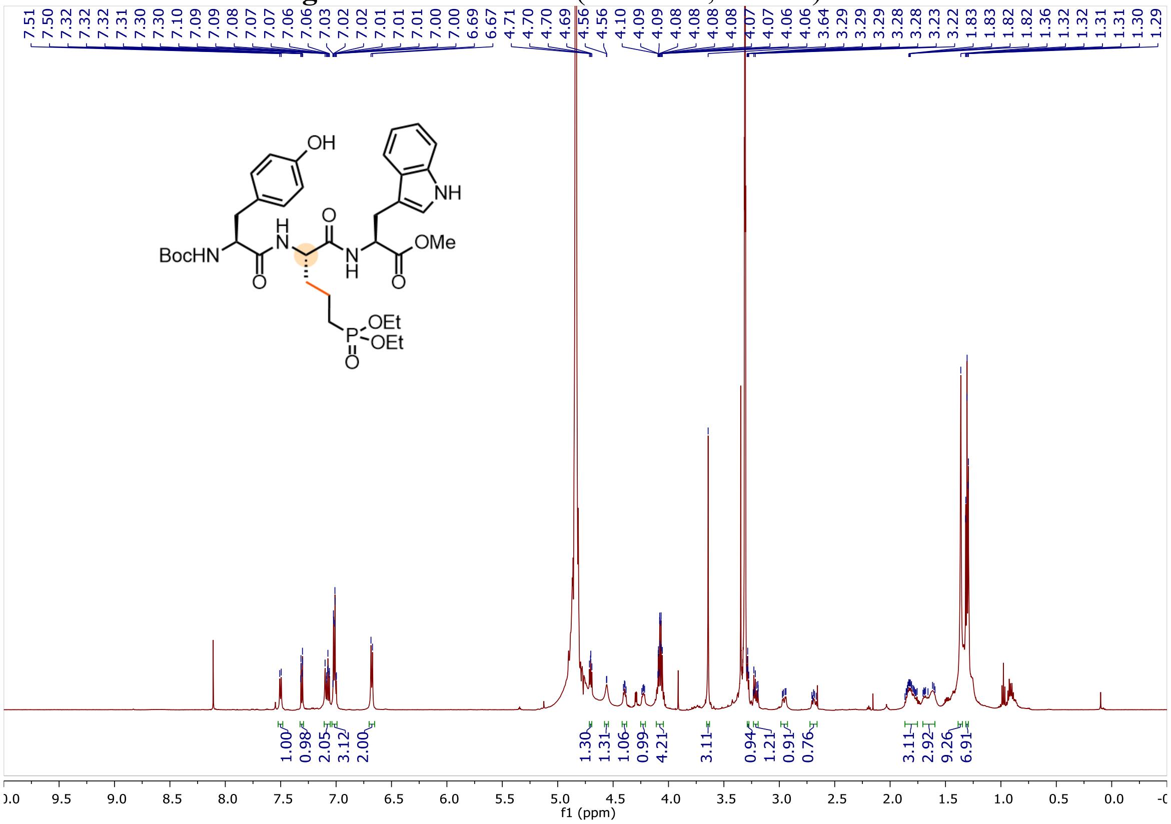

Figure S84. ^{1}**H NMR** (400 MHz, MeOD) of **41**

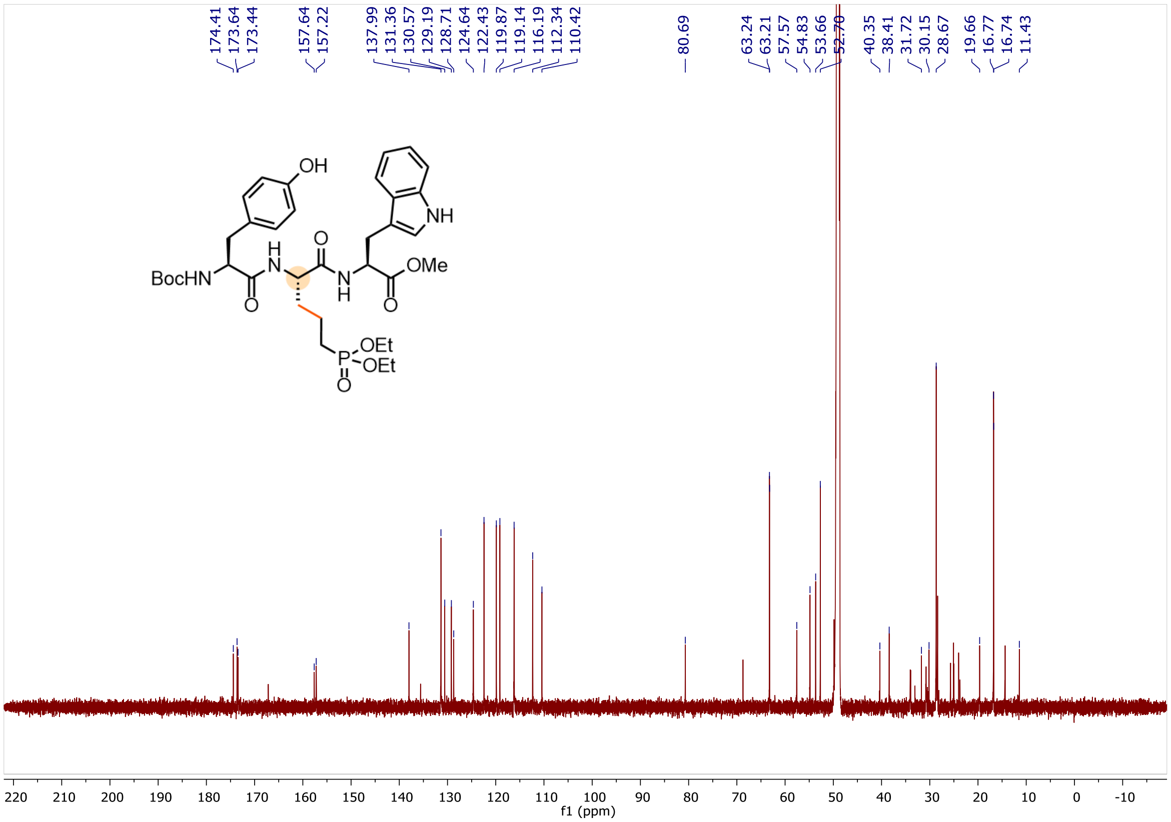

Figure S85. ^{13}**C NMR** (101 MHz, MeOD) of **41**

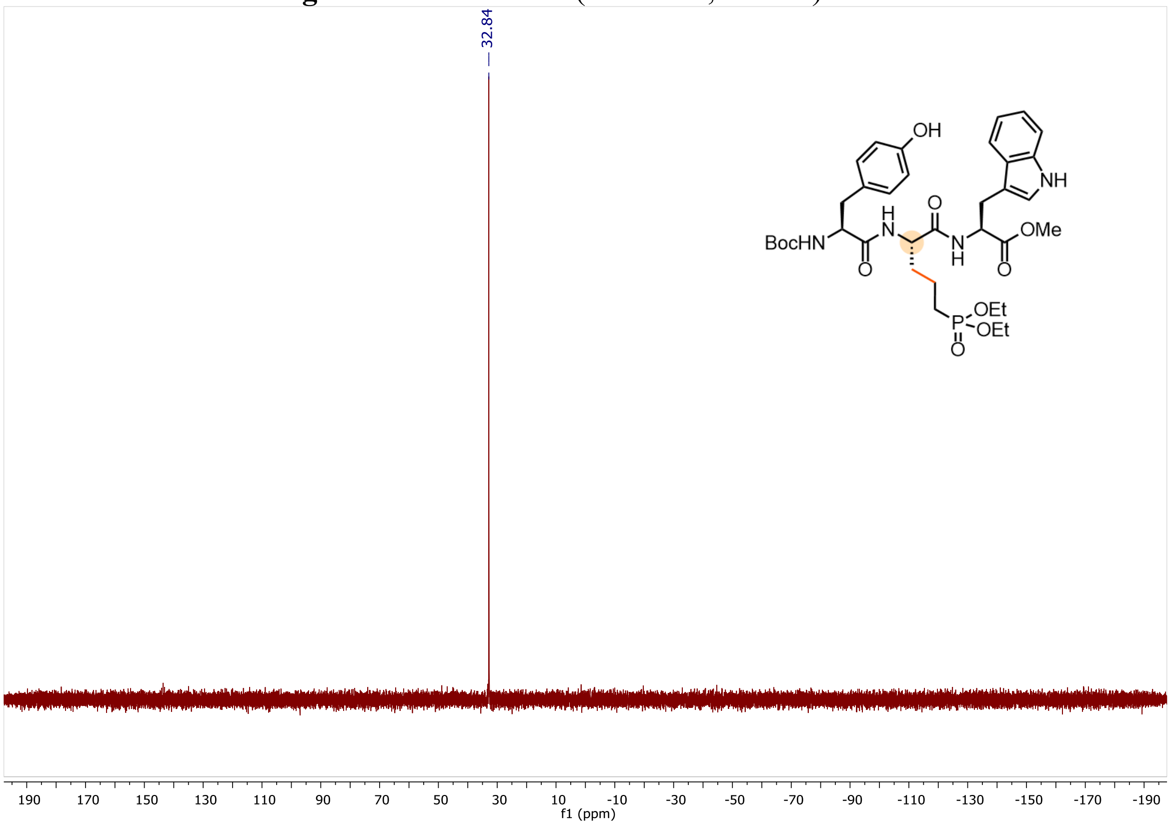

Figure S86. ^{31}**P NMR** (162 MHz, MeOD) of **41**

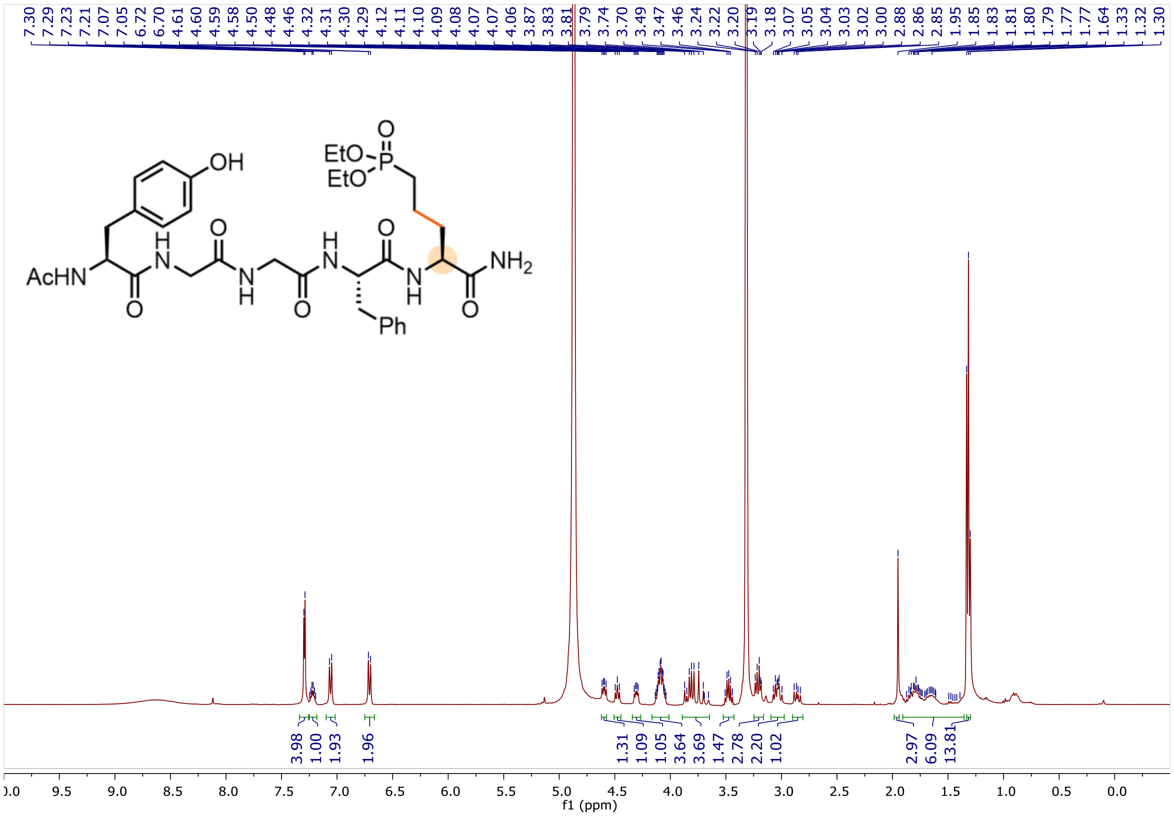

Figure S87. ^{1}**H NMR** (400 MHz, MeOD) of **42**

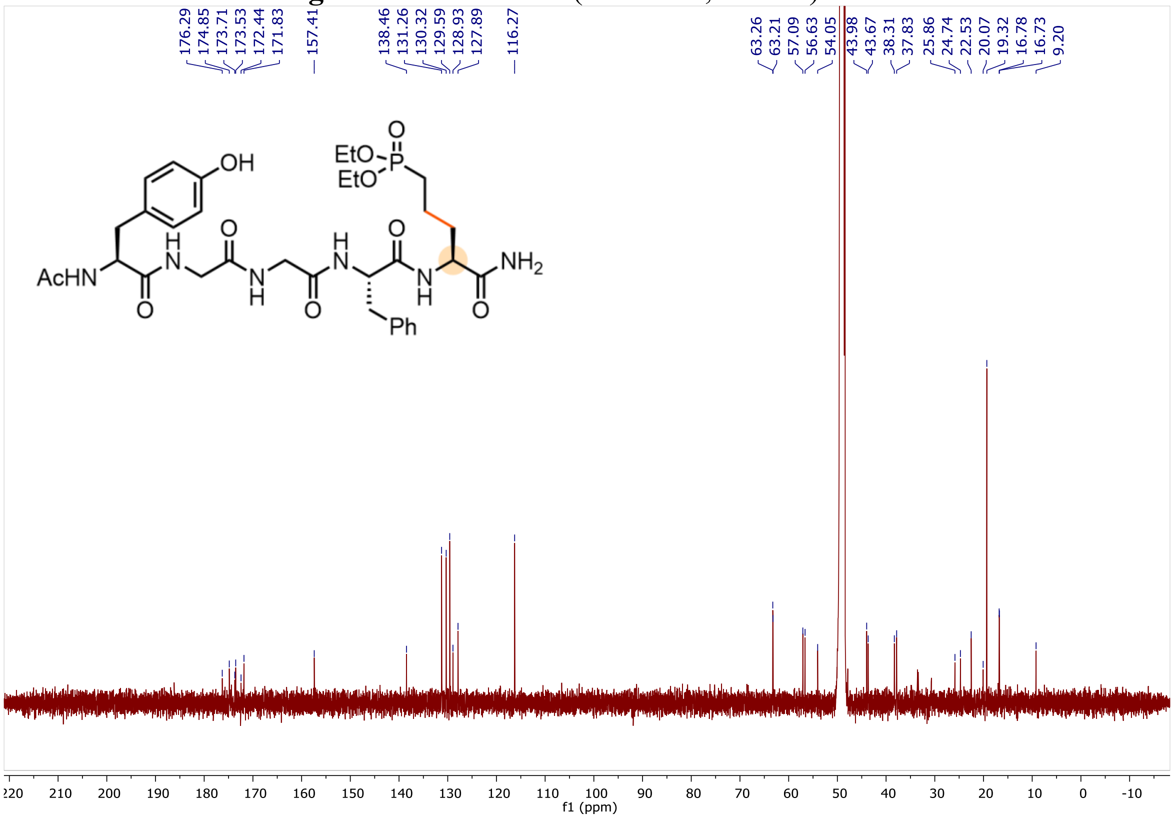

Figure S88. ^{13}**C NMR** (126 MHz, MeOD) of **42**

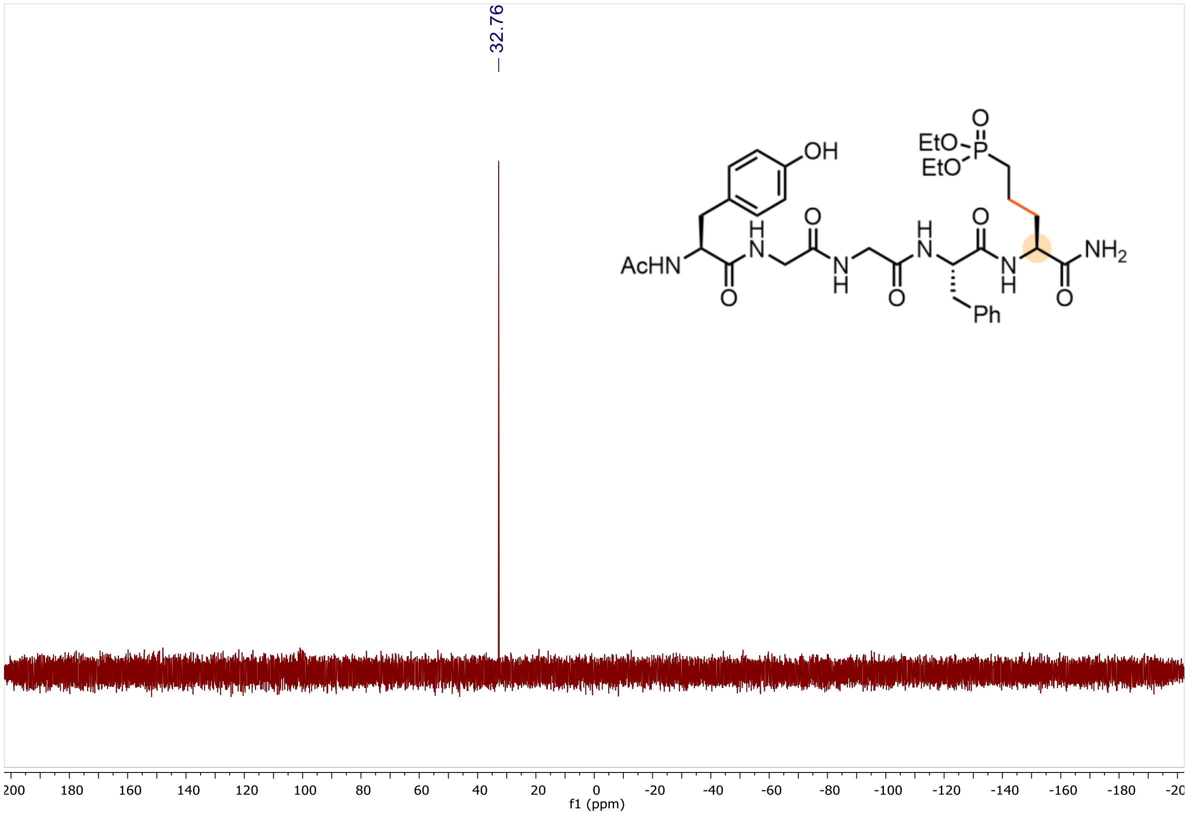

Figure S89. ^{31}P NMR (202 MHz, MeOD) of **42**

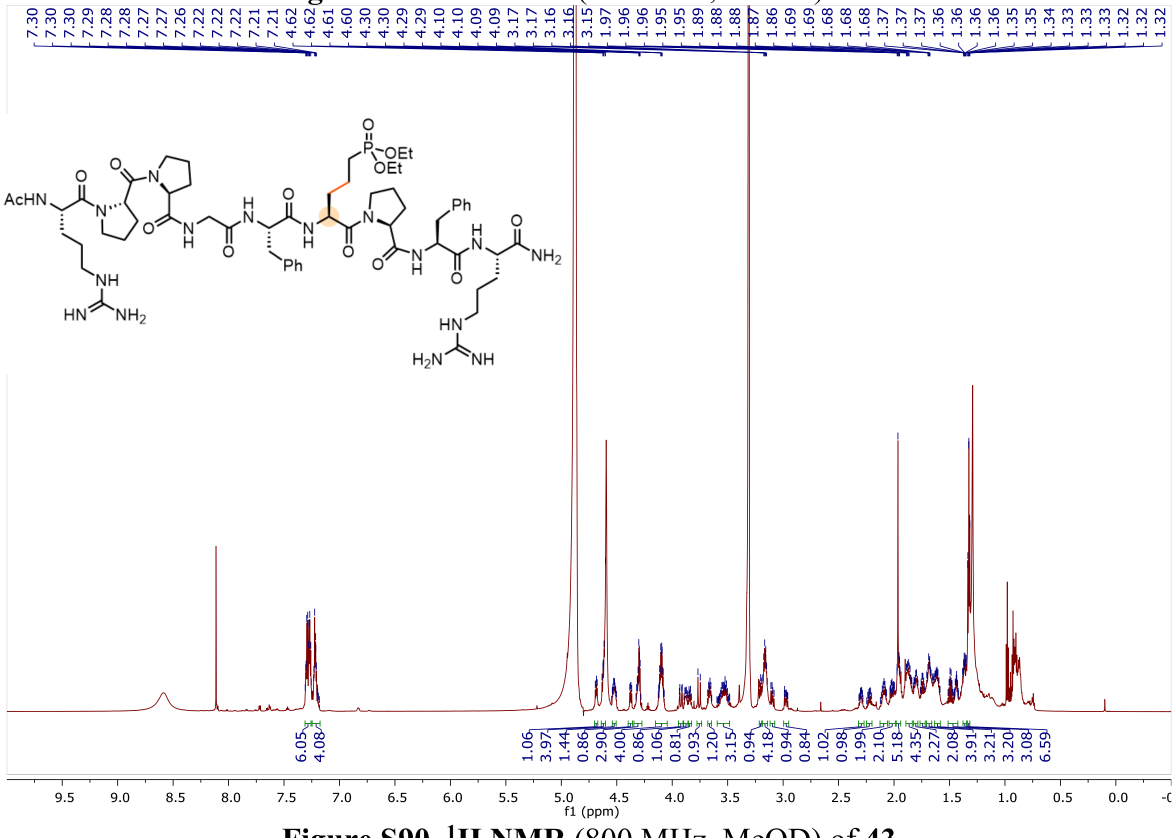

Figure S90. ^{1}H NMR (800 MHz, MeOD) of **43**

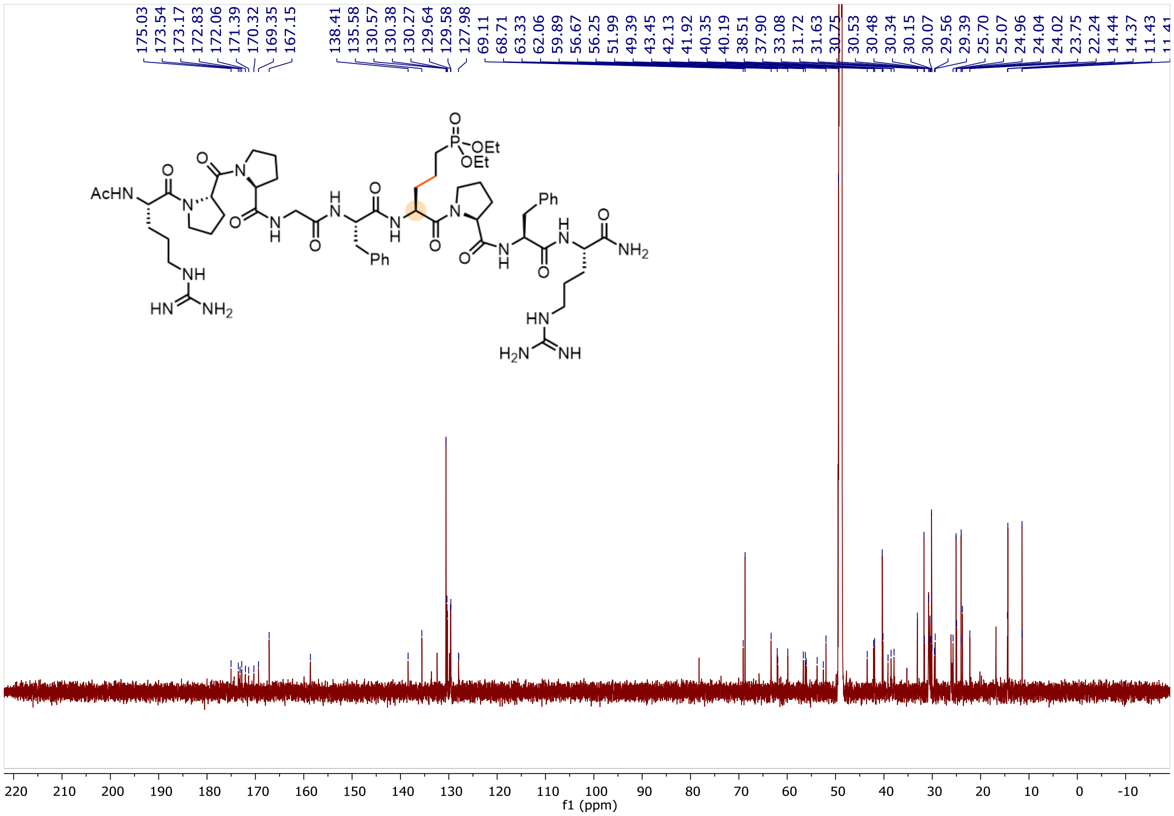

Figure S91. ^{13}**C NMR** (201 MHz, MeOD) of **43**

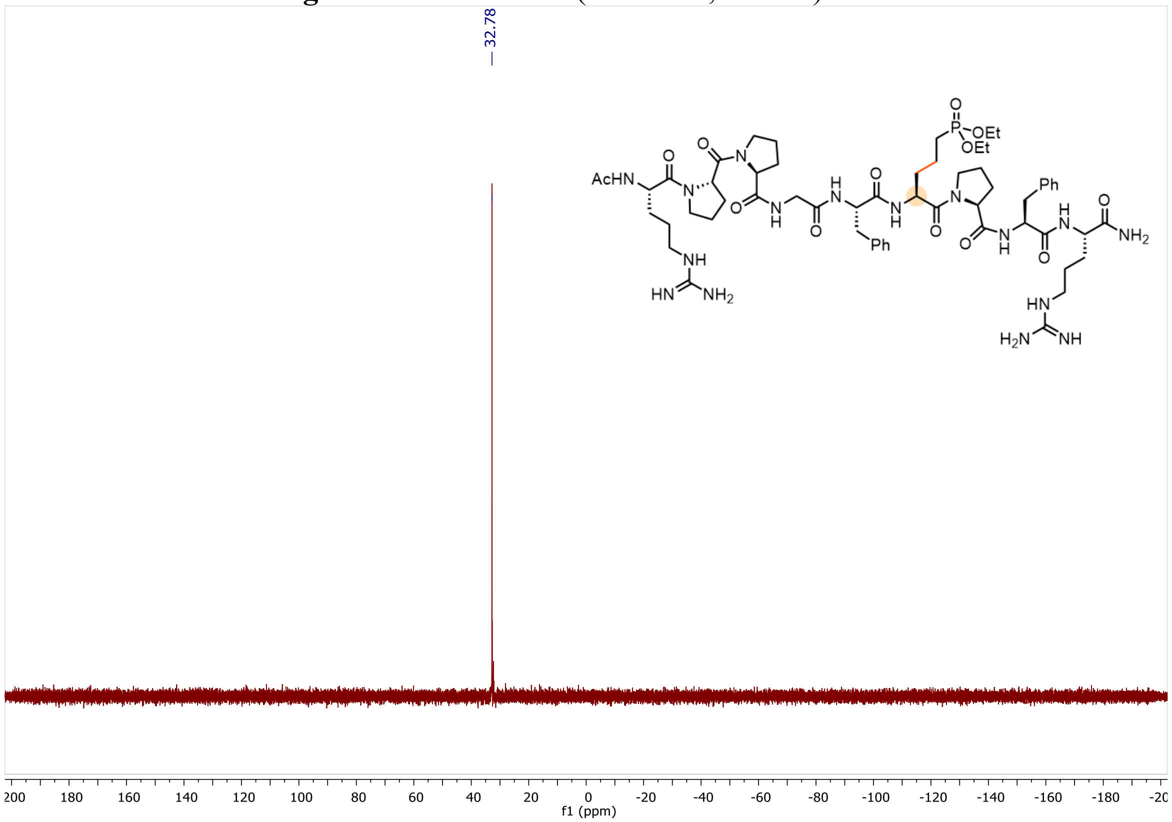

Figure S92. ^{31}**P NMR** (202 MHz, MeOD) of **43**

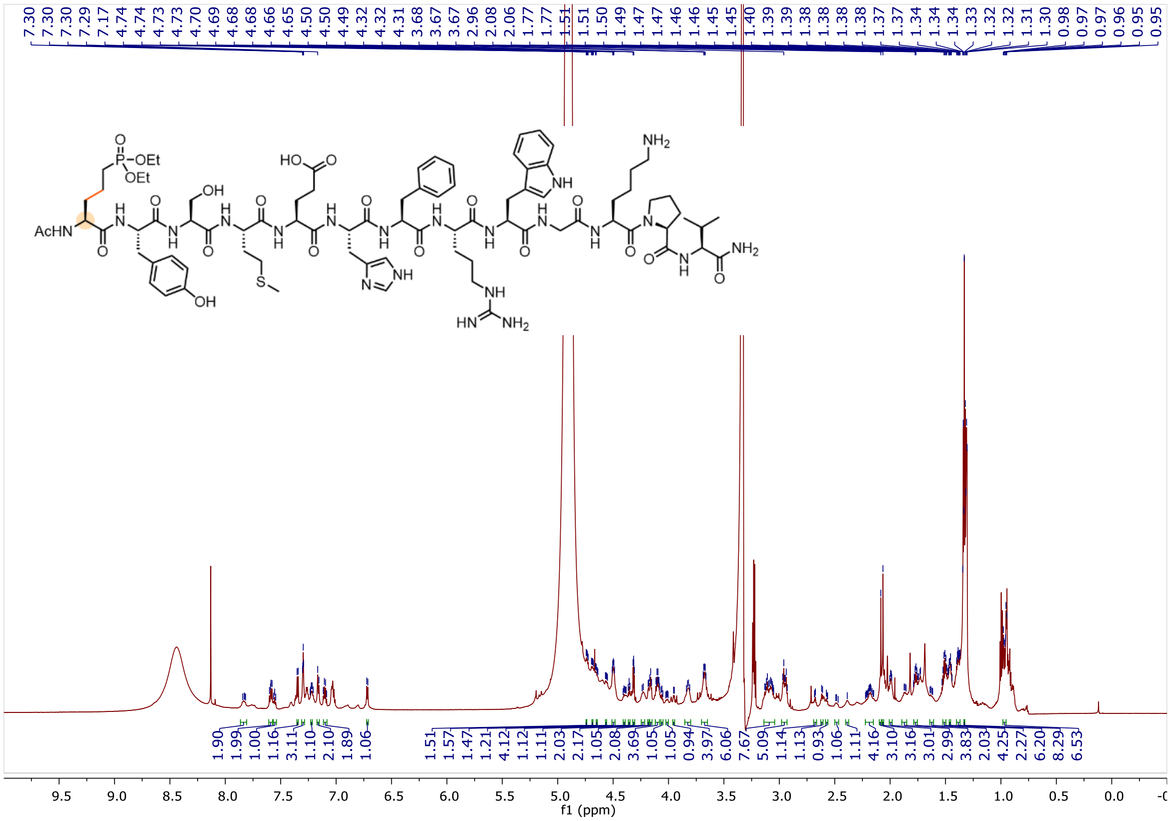

Figure S93. ^{1}H NMR (800 MHz, MeOD) of **44**

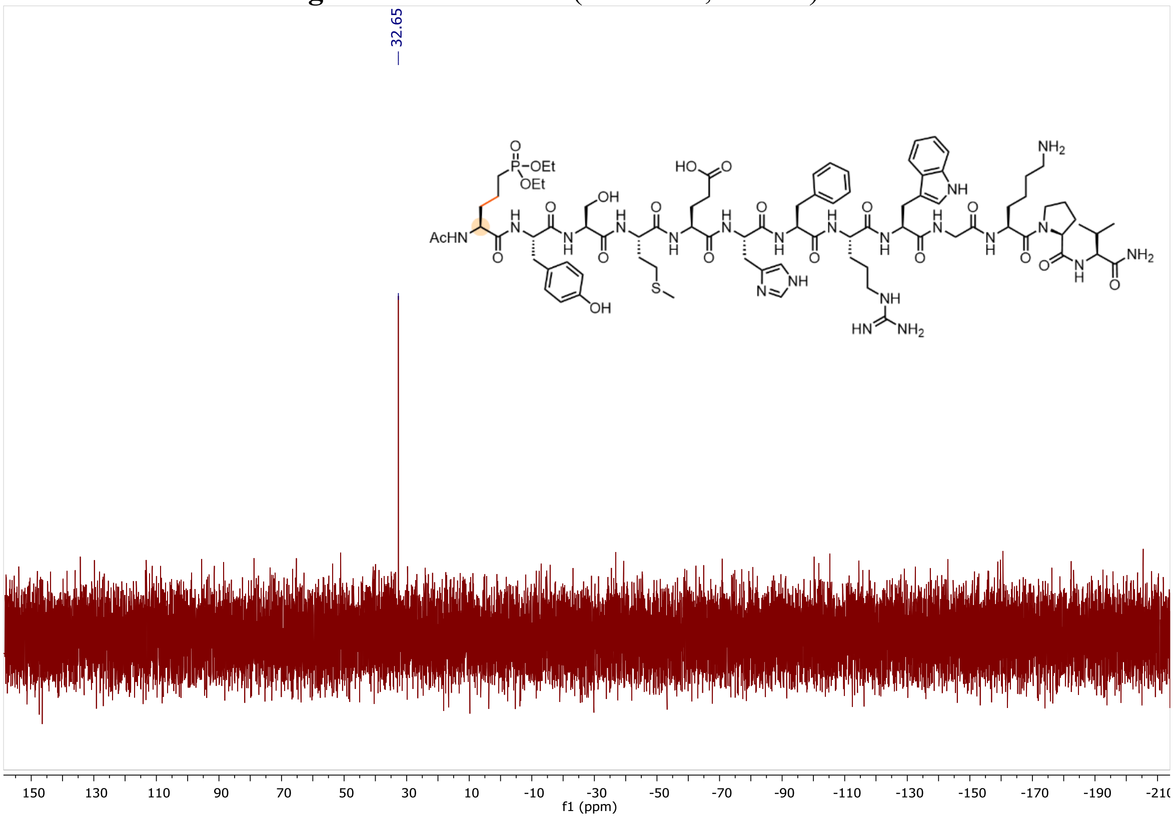

Figure S94. ^{31}P NMR (202 MHz, MeOD) of **44**

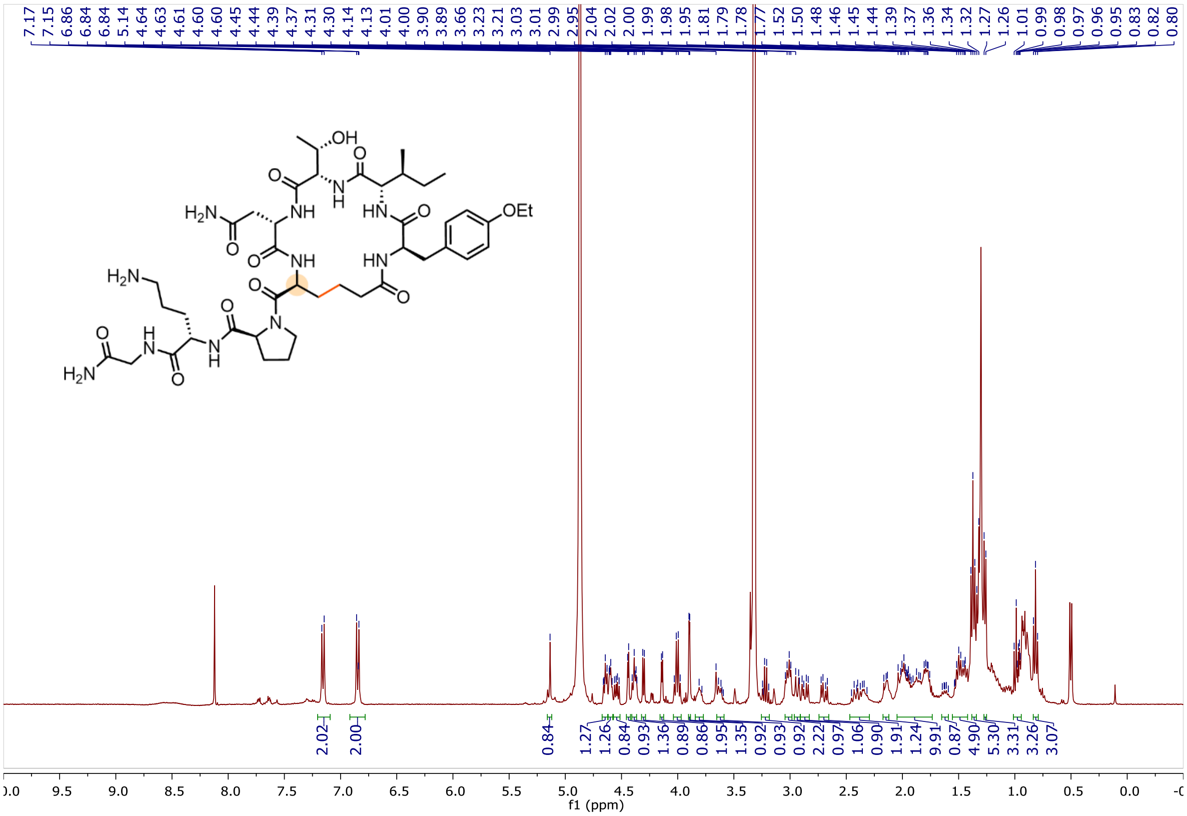

Figure S95. ^{1}H NMR (400 MHz, MeOD) of **45**

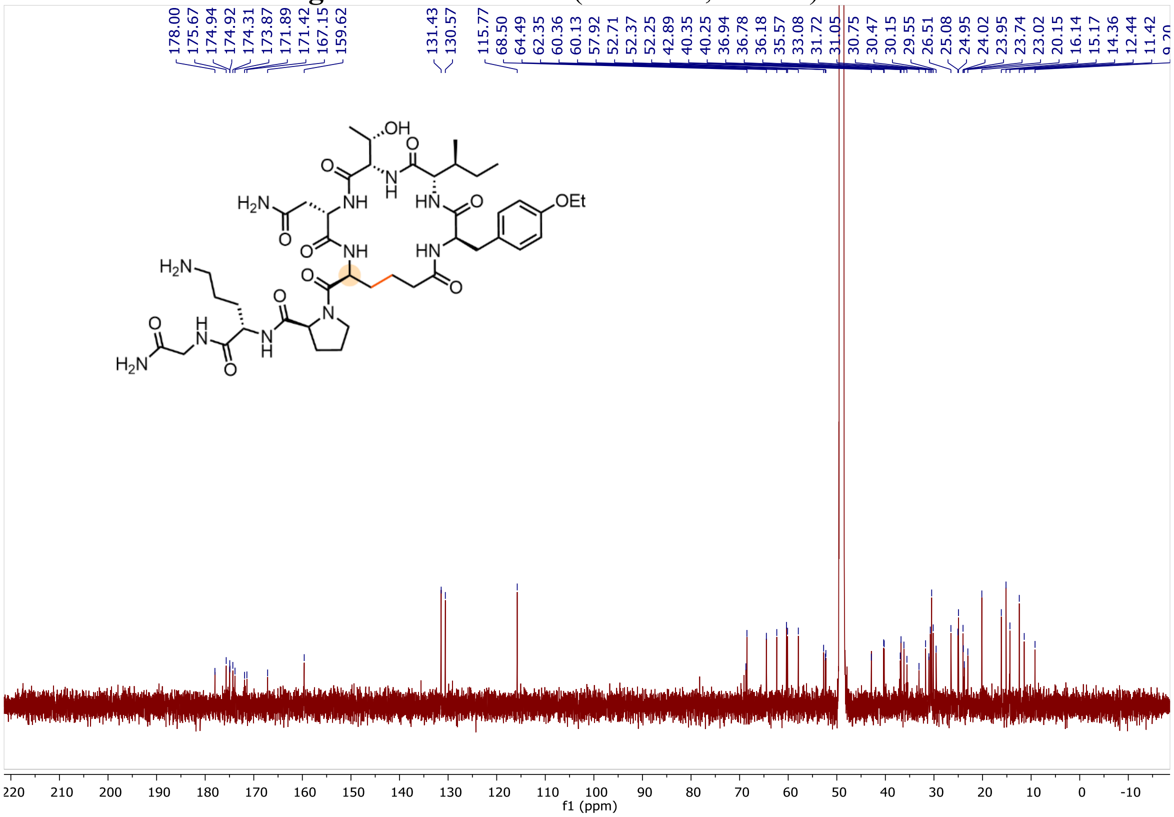

Figure S96. ^{13}C NMR (101 MHz, MeOD) of **45**

13. Reference

1. Gardner, S.; Kawamoto, T.; Curran, D. P., Synthesis of 1,3-Dialkylimidazol-2-ylidene Boranes from 1,3-Dialkylimidazolium Iodides and Sodium Borohydride. *J. Org. Chem.* **2015,** *80* (19), 9794-9797.
2. Vishnoi, P.; Sen, S.; Patwari, G. N.; Murugavel, R., Charge transfer aided selective sensing and capture of picric acid by triphenylbenzenes. *New J. Chem.* **2015,** *39* (2), 886-892.
3. Willand-Charnley, R.; Puffer, B. W.; Dussault, P. H., Oxacycle Synthesis via Intramolecular Reaction of Carbanions and Peroxides. *J. Am. Chem. Soc.* **2014,** *136* (16), 5821-5823.
4. Dellinger, D. J.; Sheehan, D. M.; Christensen, N. K.; Lindberg, J. G.; Caruthers, M. H., Solid-Phase Chemical Synthesis of Phosphonoacetate and Thiophosphonoacetate Oligodeoxynucleotides. *J. Am. Chem. Soc.* **2003,** *125* (4), 940-950.
5. Sayama, M.; Uwamizu, A.; Ikubo, M.; Chen, L.; Yan, G.; Otani, Y.; Inoue, A.; Aoki, J.; Ohwada, T., Switching Lysophosphatidylserine G Protein-Coupled Receptor Agonists to Antagonists by Acylation of the Hydrophilic Serine Amine. *J. Med. Chem.* **2021,** *64* (14), 10059-10101.
6. Jordan, P. A.; Miller, S. J., An Approach to the Site-Selective Deoxygenation of Hydroxy Groups Based on Catalytic Phosphoramidite Transfer. *Angew. Chem. Int. Ed.* **2012,** *51* (12), 2907-2911.
7. Shinkawa, Y.; Furutani, T.; Ikeda, T.; Yamawaki, M.; Morita, T.; Yoshimi, Y., Decarboxylative Side-Chain Functionalization of Aspartic/Glutamic Acids Using Two-Molecule Photoredox Catalysts. *J. Org. Chem.* **2022,** *87* (17), 11816-11825.
8. Ohshima, T.; Gnanadesikan, V.; Shibuguchi, T.; Fukuta, Y.; Nemoto, T.; Shibasaki, M., Enantioselective Syntheses of Aeruginosin 298-A and Its Analogues Using a Catalytic Asymmetric Phase-Transfer Reaction and Epoxidation. *J. Am. Chem. Soc.* **2003,** *125* (37), 11206-11207.
9. Caputo, D. F. J.; Arroniz, C.; Dürr, A. B.; Mousseau, J. J.; Stepan, A. F.; Mansfield, S. J.; Anderson, E. A., Synthesis and applications of highly functionalized 1-halo-3-substituted bicyclo[1.1.1]pentanes. *Chem. Sci.* **2018,** *9* (23), 5295-5300.
10. Elbaum, M. B.; Elkhalifa, M. A.; Molander, G. A.; Chenoweth, D. M., Solid-Phase Photochemical Peptide Homologation Cyclization. *Org. Lett.* **2022,** *24* (28), 5176-5180.
11. Li, Y.; Ye, Z.; Lin, Y.-M.; Liu, Y.; Zhang, Y.; Gong, L., Organophotocatalytic selective deuterodehalogenation of aryl or alkyl chlorides. *Nat. Commun.* **2021,** *12* (1), 2894.
12. Korsager, S.; Taaning, R. H.; Lindhardt, A. T.; Skrydstrup, T., Reductive Carbonylation of Aryl Halides Employing a Two-Chamber Reactor: A Protocol for the Synthesis of Aryl Aldehydes Including 13C- and D-Isotope Labeling. *J. Org. Chem.* **2013,** *78* (12), 6112-6120.
13. Shin, J.-A.; Kim, J.; Lee, H.; Ha, S.; Lee, H.-Y., $Cu(OTf)_2$-Promoted 1,4-Addition of Alkyl Bromides to Dehydroalanine. *J. Org. Chem.* **2019,** *84* (7), 4558-4565.
